# Factors associated with mortality in patients with atrial fibrillation: an umbrella review of systematic reviews and meta-analyses

**DOI:** 10.3389/fcvm.2026.1877770

**Published:** 2026-10-13

**Authors:** Jin Luo, Yunpu Niu, Ming Ni, Jingyu Zhang, Zhifu Guo

**Affiliations:** Department of Cardiology, Changhai Hospital, Naval Medical University, Shanghai, China

**Keywords:** arrhythmia, atrial fibrillation, mortality, risk factor, umbrella

## Abstract

**Background:**

This study synthesized published meta-analyses to assess the quality, bias, and strength of evidence regarding mortality risk factors in patients with atrial fibrillation (AF).

**Methods:**

Web of Science, Embase, PubMed, and Cochrane database were used to search the literature up to May 2025. Eligible studies were systematic reviews or meta-analyses investigating AF-related mortality risk factors. AMSTAR tool was used to assess methodological quality and GRADE was used to evaluate evidence certainty.

**Results:**

A total of 216 risk factors were identified. Among them, 86 showed significant associations with mortality, while 130 did not. Based on GRADE ratings, 11 were high-quality significant factors, and 9 were of moderate-quality. Factors linked to increased mortality included certain medications (digoxin, dronedarone, sotalol; warfarin/VKA vs. NOAC was associated with higher mortality), comorbidities (cancer, COPD, diabetes, anemia, frailty, LVH, peripheral artery disease), demographic characteristics (older age, smoking, BMI <18.5, Latin American ethnicity), and AF subtype (persistent or non-paroxysmal). In contrast, reduced mortality was associated with NOAC or DOAC use, higher BMI (25–30 or ≥30), surgical interventions (catheter ablation, LAAC/LAAO), other medications (SGLT2 inhibitors, statins), and early rhythm control.

**Conclusion:**

This review highlights key factors associated with mortality in AF. Increased risk was observed with warfarin/VKA use (vs. NOAC), certain antiarrhythmic drugs, comorbidities, low BMI, older age, smoking, and non-paroxysmal AF. In contrast, NOAC use, surgical interventions, high BMI, statin and SGLT2 inhibitor therapy, and early rhythm-control strategies were linked to reduced mortality risk.

**Systematic Review Registration:** https://www.crd.york.ac.uk/PROSPERO/view/CRD42024533372, PROSPERO CRD42024533372

## 1 Introduction

Atrial fibrillation (AF) is the most common arrhythmia globally, affecting millions and markedly elevating the risk of cardiovascular disease and death. Characterized by an irregular and often rapid heartbeat, AF can cause severe complications, like heart failure or stroke (1). Management strategies for AF generally fall into three main categories: medication-based treatments, electrophysiological procedures, and lifestyle adjustments. These strategies are all crucial in controlling the condition, reducing the risk of complications, and enhancing patients' life quality. As AF becomes more common, particularly among older adults, it is essential to thoroughly understand its risk classification and clinical impact to enhance treatment outcomes for patients.

Epidemiological data on AF demonstrate a strong association with older age, male gender, and various coexisting conditions including heart failure, high blood pressure and diabetes. These factors collectively highlight the critical need for tailored and effective treatment approaches in affected individuals (2). Several factors associated with mortality risk in individuals with AF have been identified, notably including the use of anticoagulants, antiarrhythmic agents, body mass index (BMI), and several additional clinical variables. In conclusion, although the number of meta-analyses examining potential mortality-related risk factors in AF has increased in recent years, inconsistent study designs, differences in exposure evaluation, and contradictory findings continue to hinder drawing firm conclusions.

Although meta-analyses on AF mortality risk factors show overlapping findings, inconsistent comparators, variable definitions, and mixed quality, this umbrella review systematically synthesises evidence, appraises methodological quality and certainty, and maps risk factors, clarifying robust vs. uncertain associations. By integrating this evidence, it clarifies mortality risk in AF, guides clinical decision-making, and identifies factors crucial for individualized treatment and improved outcomes in this high-risk group.

## 2 Methods and analysis

### 2.1 Design and registration

Literature searching, data extraction, and analysis were performed to examine mortality-related risk factors in AF patients according to the PRISMA guidelines (3). The methodological approach was guided by the principles specified in the Joanna Briggs Institute Manual for Evidence Synthesis of Umbrella Reviews (4), along with recommendations from the Cochrane Handbook for Systematic Reviews (5). Furthermore, this study was prospectively registered in PROSPERO (Registration number: CRD42024533372).

### 2.2 Eligibility criteria

We included meta-analyses from any country or setting that examined mortality risk factors in individuals diagnosed with AF. Factors are broadly conceptualized herein as any variables associated with mortality in patients with atrial fibrillation, including demographic characteristics, comorbidities, pharmacological treatments, surgical interventions, and lifestyle factors. This scope includes prognostic factors (e.g., age, comorbidities) as well as treatment effects (e.g., anticoagulants, ablation procedures). Since all are derived from meta-analyses of observational studies or randomised controlled trials, we applied a unified assessment framework (AMSTAR and GRADE) for quality evaluation, while interpreting the direction of associations and comparative contexts separately.

Our study elaborated on the selection rule in the Methods section as a pre-established hierarchical process. First, we preferentially selected the meta-analysis that best matches the PICO framework, then among those with comparable scope, preferentially selected the most recently published one (within a 2-year gap). Furthermore, if a single meta-analysis presented data on several risk factors, information for each was extracted separately. If several meta-analyses examined the same risk factor and were published more than two years apart, preference was given to the most recent study. Among multiple meta-analyses conducted within the same time frame, the one that included the largest number of prospective cohort studies was selected. In cases where the number of cohort studies was equal, preference was given to the meta-analysis with the higher AMSTAR score (6–8). Additionally, if the meta-analysis did not conduct a dose-response assessment, but other studies provided such results, both will be included for data extraction. Studies published in languages other than English, as well as animal or cell-based studies, were excluded from this review.

### 2.3 Population

This study focuses on systematic reviews accompanied by meta-analyses that investigate mortality-related risk factors in AF. The original studies included within these reviews should specifically examine factors linked to the risk of death in people with AF.

### 2.4 Exposure

Meta-analyses were considered eligible if they evaluated at least one mortality-related risk factor in individuals with AF, including but not limited to environmental exposures, lifestyle behaviors, comorbidities, therapeutic variables, demographic factors, social influences, and psychophysiological elements. To qualify, these studies had to report associations quantified through effect estimates—such as odds ratios (ORs), relative risks (RRs), or hazard ratios (HRs)—along with corresponding 95% confidence intervals (CIs).

### 2.5 Study designs

Only meta-analyses that investigated mortality risk factors in individuals with AF—regardless of gender, ethnicity, or geographic region—were considered for inclusion. Eligible reviews had to clearly center on AF-related mortality determinants and offer comprehensive details of their meta-analytic approach, including search strategies, selection criteria, quality evaluation methods, data synthesis techniques, and interpretation of findings. The primary studies included within these reviews comprised cohort, case-control, and cross-sectional studies.

### 2.6 Information sources

PubMed, Web of Science, Embase, and the Cochrane Database of Systematic Reviews were used for literature searching, including all publications up to May 2025 (most recent update). The search targeted meta-analyses based on interventional or observational research. To ensure completeness, the reference lists were also manually screened.

### 2.7 Search strategy

A combination of Medical Subject Headings (MeSH), keywords, and free-text terms associated with mortality in AF was used to search the databases, following the literature search standards outlined by the Scottish Intercollegiate Guidelines Network (SIGN): ((((“Atrial Fibrillation”[Mesh]) OR (((((((((Atrial Fibrillations) OR (Auricular Fibrillation)) OR (Auricular Fibrillations)) OR (Persistent Atrial Fibrillation)) OR (Persistent Atrial Fibrillations)) OR (Familial Atrial Fibrillation)) OR (Familial Atrial Fibrillations)) OR (Paroxysmal Atrial Fibrillation)) OR (Paroxysmal Atrial Fibrillations))) AND ((“Mortality”[Mesh]) OR ((((((((((((((((((((((((Mortalities) OR (Mortality Rate)) OR (Mortality Rates)) OR (Death Rate)) OR (Death Rates)) OR (Case Fatality Rate)) OR (Case Fatality Rates)) OR (CFR Case Fatality Rate)) OR (Crude Death Rate)) OR (Crude Death Rates)) OR (Crude Mortality Rate)) OR (Crude Mortality Rates)) OR (Mortality Declines)) OR (Mortality Decline)) OR (Mortality Determinants)) OR (Mortality Determinant)) OR (Differential Mortality)) OR (Differential Mortalities)) OR (Age-Specific Death Rate)) OR (Age-Specific Death Rates)) OR (Age Specific Death Rate)) OR (Excess Mortality)) OR (Excess Mortalities)) OR (Death)))) AND (systematic review OR meta-analysis)) AND (risk OR incidence OR predict) (9) (see Supplementary Table S1).

### 2.8 Study selection

Two researchers used EndNote X9 to screen records and remove duplicates, then they independently performed title and abstract screening. After that, full texts were retrieved and assessed. Our study have also supplemented the description of inter-reviewer agreement and the method for resolving disagreements through adjudication by by another investigator (see Figure 1).

**Figure 1:**
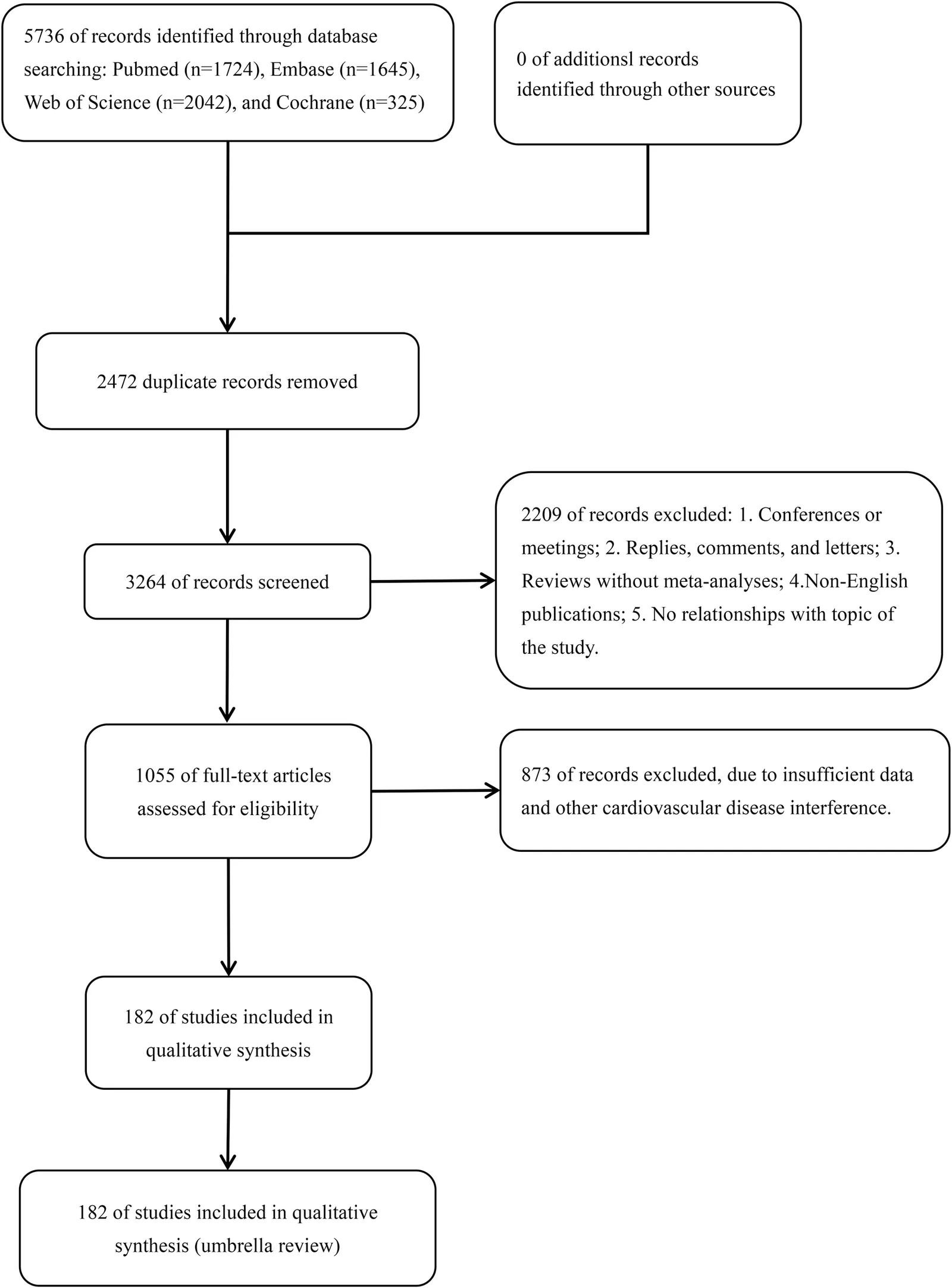
Flowchart of the systematic search and selection process.

### 2.9 Assessment of methodological quality

Two researchers independently assessed the methodological rigor using the AMSTAR tool, a validated and widely accepted instrument for evaluating the quality of meta-analyses (10, 11). In addition, the strength of evidence was examined using the GRADE, which categorizes evidence quality into four levels: high, moderate, low, or very low, to aid in drawing well-supported conclusions (12, 13). Our study have detailed the assessment process, specifying the starting level of evidence (high for RCTs, low for observational studies), downgrading factors (risk of bias, inconsistency, indirectness, imprecision, and publication bias), and upgrading factors (large effect size, dose-response gradient, and residual confounding that would attenuate the effect).

### 2.10 Data extraction

Essential data were independently extracted by two reviewers from each included meta-analysis, covering the first author's name, publication year, specific risk factors evaluated, characteristics of the study population, number of studies included, total number of cases and sample size, study design type (e.g., RCTs, cohort, case-control, cross-sectional), follow-up period, primary outcomes, and effect estimates—such as RRs, ORs, or HRs—with corresponding 95% CIs. For each risk factor, our study extracted the type of effect measure (ORs, RRs, or HRs) reported in the original meta-analysis. If the same factor was reported using different types of effect measures across different meta-analyses, we presented them separately without direct pooling. Quantitative synthesis was performed only when all available studies used the same type of effect measure. Information was also gathered on the statistical approach (random- or fixed-effects model), heterogeneity indices (Cochran's *Q* and *I*^2^ statistics), and methods for evaluating publication bias (e.g., funnel plots, Egger's and Begg's tests). For analyses reporting dose-response or subgroup results, we additionally extracted the *p*-value for nonlinearity and subgroup-specific effect sizes.

### 2.11 Data summary

RRs, ORs, and HRs, along with their corresponding 95% CIs, were recalculated using either a fixed-effects or random-effects model based on the degree of heterogeneity. The extent of heterogeneity was determined using the *I*^2^ statistic and Cochran's *Q* test. For meta-analyses or systematic reviews comprising more than 10 studies, potential small-study effects were examined using Egger's test or Begg's test (14–16). Sensitivity analyses were conducted, when sufficient data permitted, for risk factors rated as high or moderate-quality, to evaluate the effect of each study on the pooled results. Dose–response analyses related to AF mortality, when available, were also extracted. In cases where the latest meta-analysis did not incorporate clinical trials that were part of earlier reviews, data from those omitted studies were combined and reanalyzed. Heterogeneity was deemed statistically significant when *P* < 0.10, whereas other statistical tests applied a threshold of *P* < 0.05. Review Manager version 5.4 (Cochrane, Oxford, UK) was used to synthesize data, while Stata version 15.1 was employed to perform sensitivity analyses and assess small-study effects.

## 3 Major outcomes

### 3.1 Characteristics of meta-analyses

Figure 1 presents the literature screening and selection process. A total of 5,736 unique articles were retrieved. Based on the inclusion criteria, 182 meta-analyses were ultimately included. A total of 216 distinct risk factors were identified—of which 86 demonstrated statistically significant associations with mortality, while the remaining 130 did not (Supplementary Tables S2, S6). Among the significant associations, 32 were harmful and 54 were protective. Based on GRADE assessments, most studies were rated as low or very low quality. However, 30 risk factors were supported by moderate-quality evidence, and 11 were backed by high-quality evidence. Notably, most of the high-certainty risk factors associated with AF-related mortality involved the use of anticoagulant or antiplatelet agents.

### 3.2 Anticoagulants or antiplatelets

For the associations supported by the high-quality evidence, DOAC use was significantly associated with lower all-cause mortality compared with warfarin in AF patients with a BMI between 25 and 30 kg/m^2^ (HR: 0.84, 95% CI: 0.76–0.92) (17). Similarly, NOAC use was associated with lower mortality than warfarin in patients with time in therapeutic range (TTR) below 65% (RR: 0.85, 95% CI: 0.76–0.93) and when combined with aspirin (HR: 0.85, 95% CI: 0.76–0.93) (18, 19). Furthermore, a meta-analysis of 12 RCTs demonstrated that NOAC, excluding ximelagatran, were associated with a reduction in all-cause mortality vs. controls (OR: 0.89, 95% CI: 0.84–0.95), and another pooled analysis of six RCTs found that oral factor Xa inhibitors were associated with lower mortality compared with vitamin K antagonists (VKAs) (OR: 0.88, 95% CI: 0.83–0.94) (20, 21) (Figure 2).

**Figure 2:**
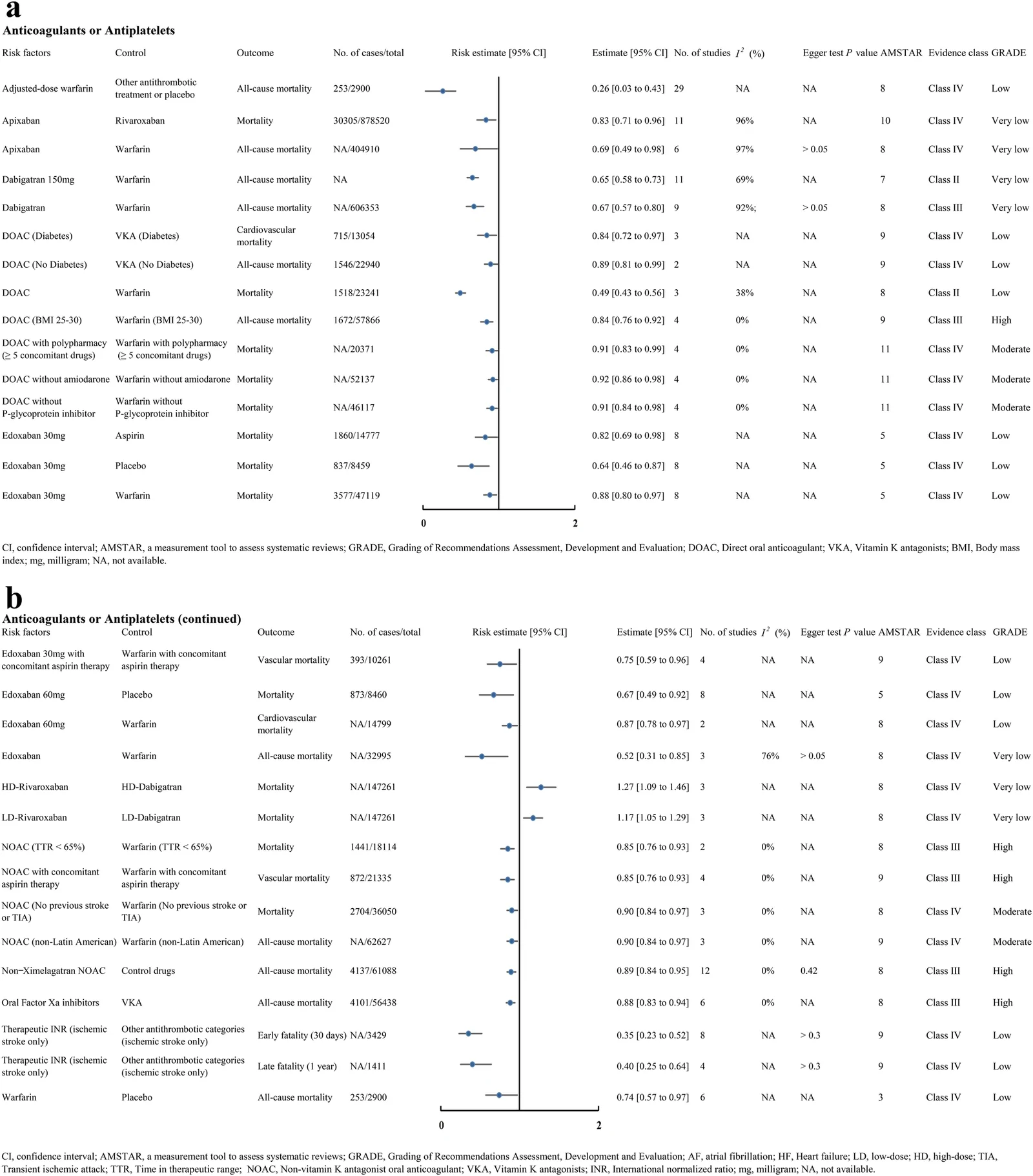
Forest plots of significant-associated risk factors of mortality in anticoagulants or antiplatelets for AF.

moderate-quality evidence further supported the association between DOAC use and lower mortality in specific subgroups: patients taking ≥5 concomitant medications (RR: 0.91, 95% CI: 0.83–0.99), those not on amiodarone (RR: 0.92, 95% CI: 0.86–0.98), and those not using P-glycoprotein inhibitors (RR: 0.91, 95% CI: 0.84–0.98) all showed significant associations with mortality reduction vs. warfarin (22). NOAC were also associated with lower mortality compared with warfarin in AF patients without prior stroke or transient ischaemic attack (RR :0.90, 95% CI: 0.84–0.97) and in non-Latino populations (RR: 0.90, 95% CI: 0.84–0.97) (18, 23) (Figure 2).

For the remaining comparisons, the available evidence was generally of low or very low certainty. Adjusted-dose warfarin was associated with lower all-cause mortality vs. placebo or other antithrombotic therapies (OR: 0.26, 95% CI: 0.03–0.43) (24). Edoxaban at both 30 mg and 60 mg doses was associated with lower mortality vs. aspirin, placebo, or warfarin, with RRs ranging from 0.52 to 0.88 across various comparisons (19, 25–27). Apixaban was associated with lower mortality than rivaroxaban (RR: 0.83, 95% CI: 0.71–0.96) and warfarin (HR: 0.69, 95% CI: 0.49–0.98) (25, 28); dabigatran 150 mg was associated with lower mortality than warfarin (HRs: 0.65 and 0.67) (25, 29);whereas rivaroxaban (high or low dose) was associated with higher mortality than dabigatran (HRs: 1.17–1.27) (30). A pooled estimate indicated that overall DOAC use was associated with reduced mortality (OR: 0.49, 95% CI: 0.43–0.56) (31). Achieving therapeutic INR was associated with lower early (≤30 days) and late (≤1 year) mortality (OR: 0.35 and 0.40, respectively) (32). Additionally, DOAC were associated with lower mortality in both diabetic and non-diabetic patients vs. VKAs (RRs 0.84 and 0.89, respectively), though evidence quality was low (33) (Figure 2).

Collectively, high and moderate-quality evidence consistently supports the association between DOAC/NOAC use and lower mortality over warfarin or VKAs, particularly in specific patient subgroups, whereas many other associations rely on low certainty data and warrant cautious interpretation.

### 3.3 Antiarrhythmics

moderate-quality evidence from a meta-analysis of five cohort studies showed that sotalol was associated with an increased risk of all-cause mortality compared with placebo or no therapy (RR: 2.23, 95% CI: 1.03–4.81), and a similar association was observed in patients with paroxysmal AF (RR: 2.51, 95% CI: 1.06–5.98) (34). A meta-analysis of 13 RCTs also reported a similar association (OR: 2.23, 95% CI: 1.10–4.50; low quality) (34) (Figure 3).

**Figure 3:**
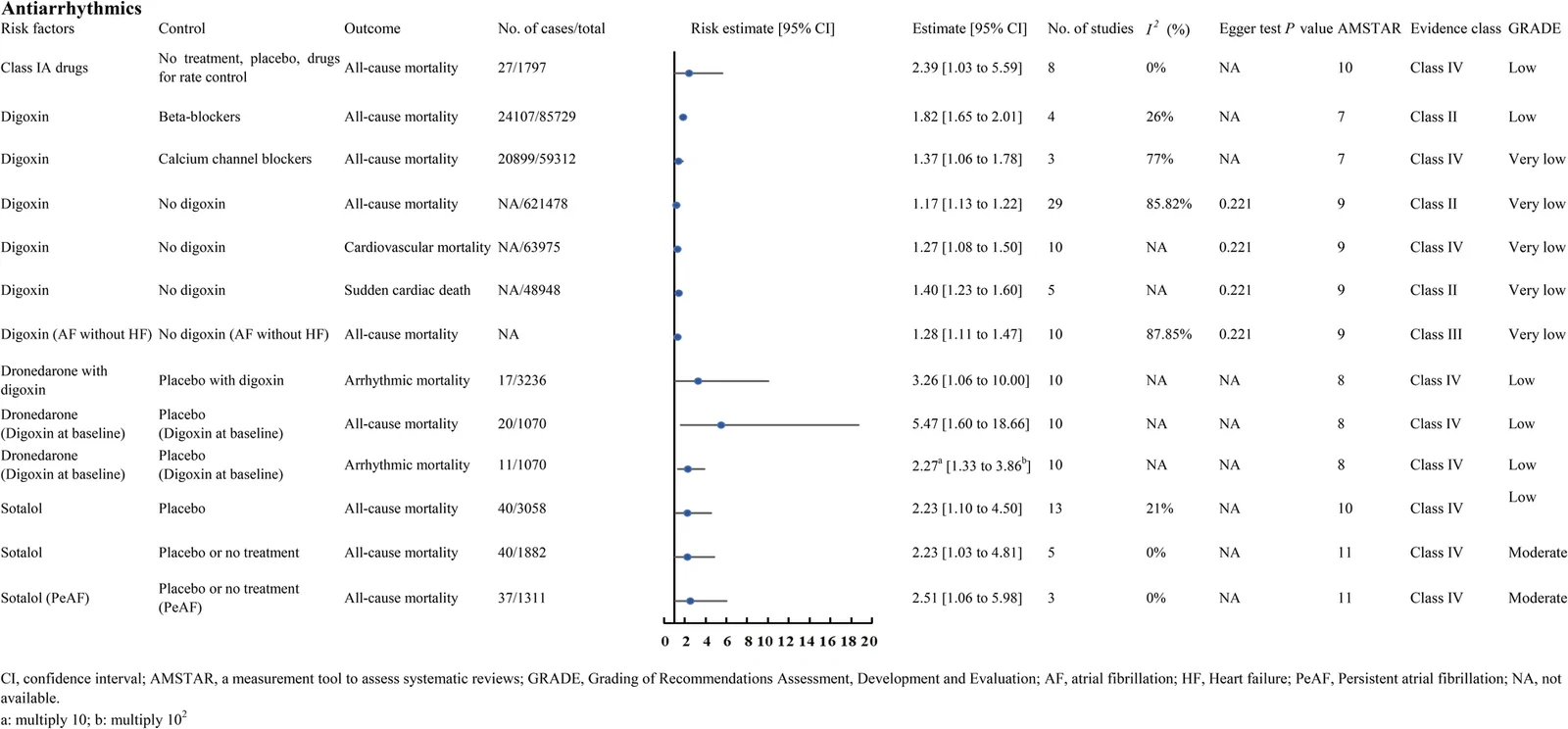
Forest plots of significant-associated risk factors of mortality in antiarrhythmics for AF.

Low-to-very-low quality evidence indicated that class IA agents were associated with higher mortality vs. no therapy, placebo, or rate control (OR: 2.39, 95% CI: 1.03–5.59) (34). Digoxin was associated with increased mortality compared with beta-blockers (RR: 1.82, 95% CI: 1.65–2.01) and calcium channel blockers (RR: 1.37, 95% CI: 1.06–1.78) (35); its use was also linked to higher all-cause mortality (HR: 1.17, 95% CI: 1.13–1.22), cardiovascular death (HR: 1.40, 95% CI: 1.23–1.60), and sudden cardiac death (HR: 1.82, 95% CI: 1.65–2.01), with similar findings in patients without heart failure (HR: 1.28, 95% CI: 1.11–1.47) (36). Dronedarone was associated with increased arrhythmic mortality vs. placebo plus digoxin (HR: 3.26, 95% CI: 1.06–10.00) and with higher all-cause and arrhythmic mortality vs. baseline digoxin users (HR: 5.47 and 22.7, respectively) (37) (Figure 3). Overall, sotalol was the only agent with moderate-quality evidence, while most other associations were supported by low-certainty data.

### 3.4 Comorbid disease

Several comorbidities were associated with increased all-cause mortality in AF patients, including diabetes (RR: 1.37, 95% CI: 1.23–1.53), anaemia (HR: 1.78, 95% CI: 1.44–2.20), cancer (OR: 2.17, 95% CI: 1.83–2.56), COPD (OR: 2.22, 95% CI: 1.93–2.55), frailty (RR: 2.77, 95% CI: 1.68–4.57), LVH (RR: 1.60, 95% CI: 1.42–1.79), and peripheral artery disease (HR: 1.42, 95% CI: 1.25–1.62), with most estimates based on low- or very-low-certainty evidence (38–44). Additionally, diabetes (RR: 1.46, 95% CI: 1.34–1.58) and COPD (RR: 1.84, 95% CI: 1.39–2.43) were associated with cardiovascular mortality, while cancer was associated with non-cardiovascular mortality (RR: 6.30, 95% CI: 2.32–17.33), all with very-low certainty (38, 40, 41) (Figure 4).

**Figure 4:**
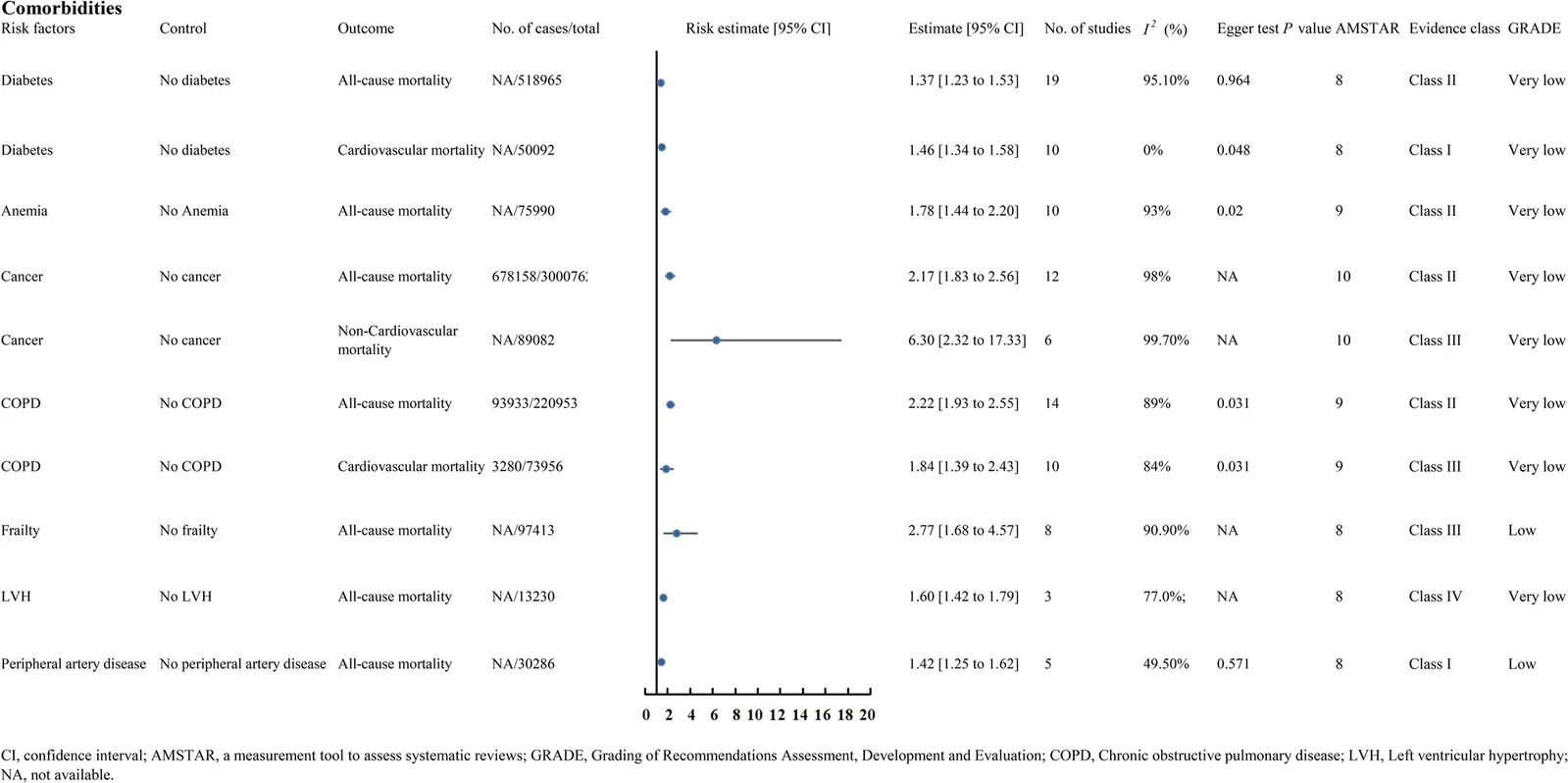
Forest plots of significant-associated risk factors of mortality in comorbid disease for AF.

### 3.5 Demographic characteristics

This section examines the relationships between demographic characteristics and mortality in patients with AF, focusing on BMI, ethnicity, smoking, and age. Among these, the association with Latin American ethnicity stands out as the most consistently supported by high certainty evidence: multiple meta-analyses showed that Latin American patients had higher all-cause mortality than non-Latin American patients, regardless of whether they received warfarin (RR: 1.45, 95% CI: 1.20–1.76), NOAC (RR: 1.46, 95% CI: 1.27–1.68), or either (RR: 1.46, 95% CI: 1.30–1.63) (23). The higher mortality observed in Latino patients may reflect confounding factors such as access to healthcare resources, quality of anticoagulation control, and comorbidity burden, rather than ethnicity itself being an independent risk factor. In contrast, the remaining associations were supported by low-to-very-low certainty data. Higher BMI was associated with lower all-cause mortality compared with low BMI (RR: 0.86, 95% CI: 0.80–0.92), as well as with reduced cardiovascular mortality (RR: 0.82, 95% CI: 0.71–0.95) (45). Both overweight (RR: 0.78, 95% CI: 0.62–0.96) and obesity (RR: 0.61, 95% CI: 0.52–0.71) showed protective associations vs. normal BMI, whereas underweight was associated with substantially higher mortality (RR: 3.57, 95% CI: 2.50–5.11) (46, 47). Smoking was associated with increased all-cause (RR: 1.82, 1.33–2.49) and cardiovascular (RR: 1.54, 95% CI: 1.31–1.81) mortality (48), and older age (≥75 years) with higher all-cause mortality compared with younger groups (RR: 2.61, 95% CI: 1.38–4.93) (49) (Figure 5).

**Figure 5:**
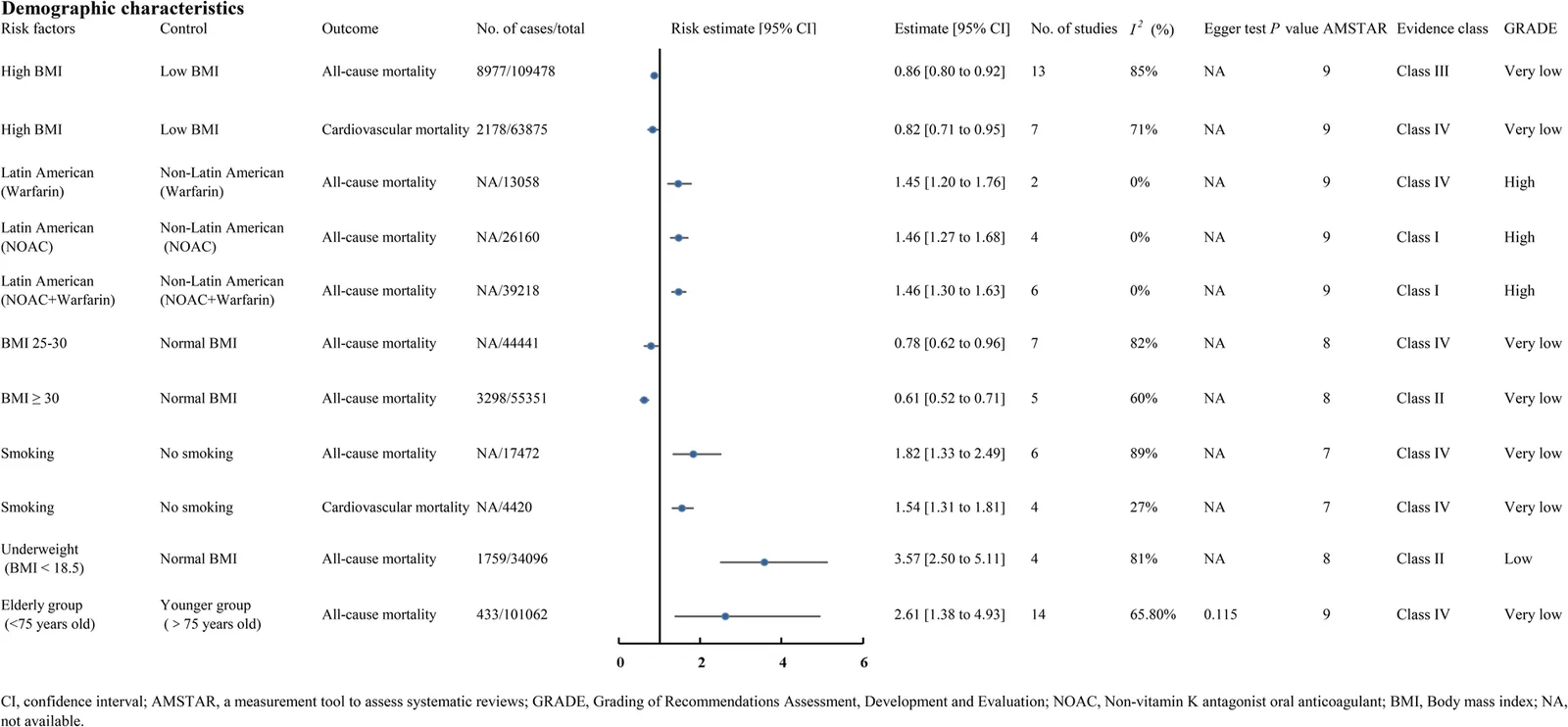
Forest plots of significant-associated risk factors of mortality in demographic characteristics for AF.

### 3.6 Surgery

The most robust evidence indicated that ablation was associated with lower all-cause mortality compared with antiarrhythmic drug therapy (RR: 0.46, 95% CI: 0.23–0.93), and this benefit was also observed in patients with heart failure (RR: 0.52, 95% CI: 0.35–0.76) (50). Moderate certainty data further supported this association, showing reduced mortality with ablation vs. medical therapy regardless of heart failure status (RR: 0.69, 95% CI: 0.54–0.88) (50). For other comparisons, the evidence was of low-to-very-low certainty: ablation was associated with lower cardiovascular (HR: 0.35, 95% CI: 0.25–0.50) and all-cause (HR: 0.64, 95% CI: 0.45–0.93) mortality vs. drug therapy, and similar associations were observed vs. non-ablation (HR: 0.60, 95% CI: 0.51–0.71) and in patients with reduced LVEF (RR: 0.56, 95% CI: 0.39–0.79) (50–52) (Figure 6A).

**Figure 6:**
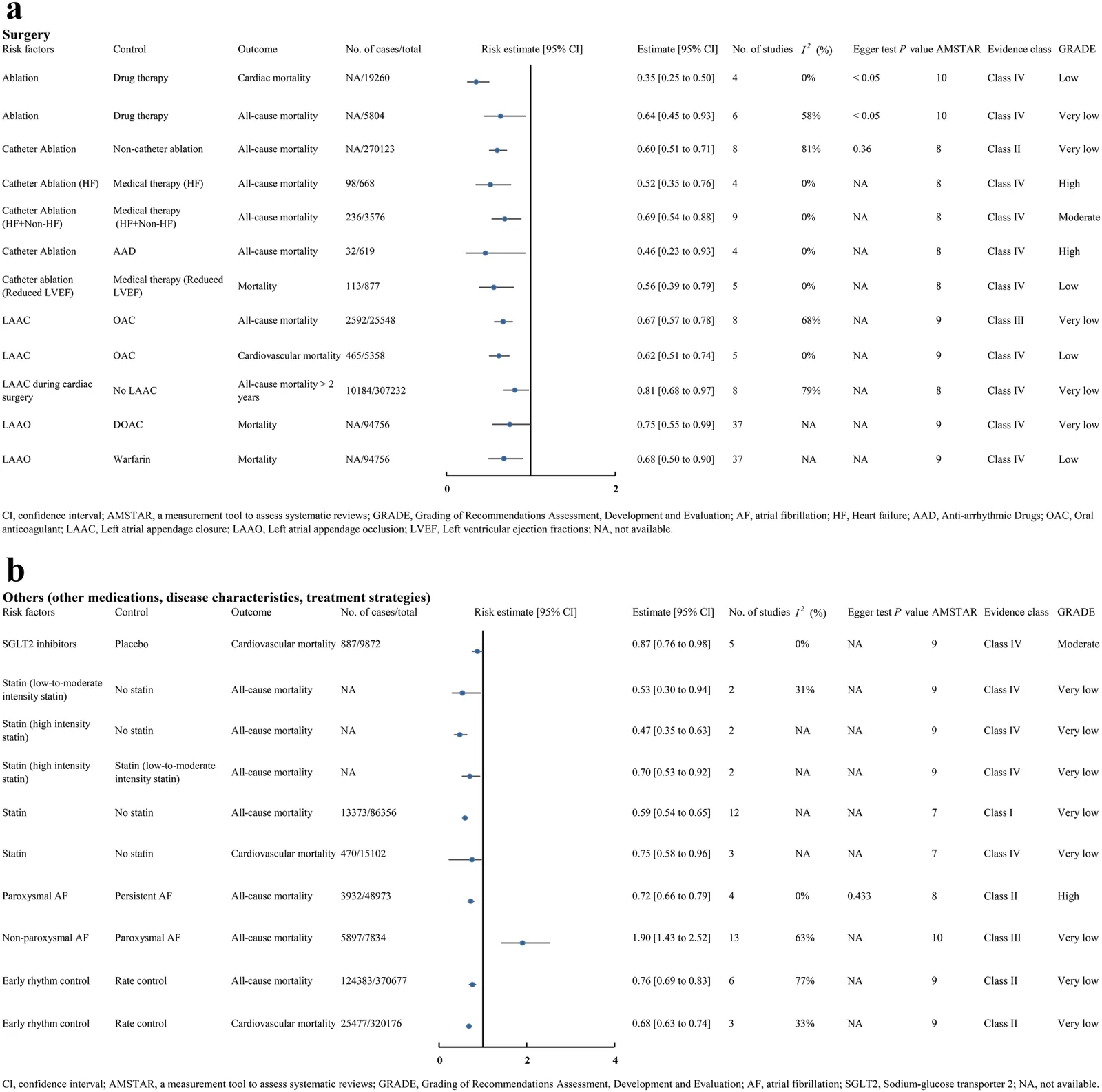
Forest plots of significant-associated risk factors of mortality. **(a)** Surgery for AF; **(b)** others for AF.

Regarding left atrial appendage closure or occlusion (LAAC/LAAO), the evidence was uniformly of low-to-very-low certainty. LAAC was associated with lower all-cause mortality compared with oral anticoagulants (RR: 0.67, 95% CI: 0.57–0.78) and lower cardiovascular mortality (HR: 0.62, 95% CI: 0.51–0.74) (53). Similar protective associations were seen vs. warfarin (OR: 0.68, 95% CI: 0.50–0.90) and vs. DOAC (OR: 0.75, 95% CI: 0.55–0.99) (54). Additionally, LAAC performed during cardiac surgery was associated with reduced all-cause mortality at >2 years compared with no LAAC (RR: 0.81, 95% CI: 0.68–0.97) (55) (Figure 6A).

### 3.7 Other outcomes

Several pharmacological agents were also examined in relation to mortality in AF patients. Moderate certainty evidence from a meta-analysis of five RCTs indicated that SGLT2 inhibitors were associated with lower cardiovascular mortality compared with placebo (RR: 0.87, 95% CI: 0.76–0.98) (56). For statins, the evidence was generally of very low certainty, yet consistently showed associations with reduced mortality. Statin use was linked to lower all-cause (HR: 0.59, 95% CI: 0.54–0.65) and cardiovascular (HR: 0.75, 95% CI: 0.58–0.96) mortality vs. no statin use (57). High-intensity statins were associated with lower all-cause mortality compared with low-to-moderate intensity (HR: 0.70, 95% CI: 0.53–0.92) or no statin use (HR: 0.47, 95% CI: 0.35–0.63), and even low-to-moderate intensity showed a similar benefit vs. no statin use (HR: 0.53, 95% CI: 0.30–0.94) (58) (Figure 6B).

Regarding disease characteristics and management strategies, the most robust evidence (high certainty) showed that paroxysmal AF was associated with lower all-cause mortality than persistent AF (RR: 0.72, 95% CI: 0.66–0.79) (59). In contrast, non-paroxysmal AF was associated with higher mortality compared with paroxysmal AF (OR: 1.90, 95% CI: 1.43–2.52), though this was based on very-low-certainty evidence (60). For treatment strategy, very-low-certainty evidence indicated that early rhythm control was associated with reduced all-cause (RR: 0.76, 95% CI: 0.69–0.83) and cardiovascular (RR: 0.68, 95% CI: 0.63–0.74) mortality compared with rate control (61) (Figure 6B).

### 3.8 Non-significant risk factors

A total of 130 risk factors with no significant association were extracted in this study. These risk factors followed the same classification as above, including anticoagulants or antiplatelets, antiarrhythmics, surgical interventions, comorbid diseases, demographic characteristics, and other outcomes. There is currently no definite evidence that the above factors are strongly linked to mortality in AF patients (Supplementary Table S2).

### 3.9 Heterogeneity

We reexamined heterogeneity for 73.1% of all identified risk factors. Among these, approximately 40.3% of the risk factors showed significant heterogeneity, defined as *P* < 0.1 or *I*^2^ > 50%. The observed heterogeneity was primarily attributed to differences in study setting, geographic region, ethnicity, sex, age, study quality, design, sample size, follow-up duration, and adjustments for confounding variables. Additionally, for 26.9% of the risk factors, heterogeneity could not be reassessed because the original studies did not provide heterogeneity assessment results.

### 3.10 Assessment of risk of bias

We performed Egger's test for 19.4% of the risk factors in our reanalysis, which detected evidence of publication bias in 11 risk factors, including ablation vs. drug therapy (all-cause mortality and cardiovascular mortality); diabetes vs. no diabetes (cardiovascular mortality); anemia vs. no anemia (all-cause mortality); COPD vs. no COPD (all-cause and cardiovascular mortality); beta-blocker use with COPD vs. no beta-blocker use with COPD (all-cause and cardiovascular mortality); beta-blocker use without COPD vs. no beta-blocker use without COPD (all-cause and cardiovascular mortality); and RAAS blocker use vs. no RAAS blocker use (cardiovascular mortality).

For the outcomes that could not be reanalyzed, publication bias was identified in 22.2% of the risk factors based on statistical tests or funnel plot assessments. The remaining outcomes either demonstrated no significant evidence of publication bias or lacked any reported evaluation of it.

### 3.11 AMSTAR and GRADE

The median AMSTAR score of all risk factors was 7 (range 3–11) (Supplementary Table S3). In terms of GRADE, DOAC use with BMI 25–30 vs. warfarin use with BMI 25–30, NOAC use with TTR <65% vs. warfarin use with TTR <65%, NOAC with concomitant aspirin therapy vs. warfarin with concomitant aspirin therapy, non-ximelagatran NOAC vs. control drugs, oral factor Xa inhibitors vs. VKAs, catheter ablation vs. AAD, catheter ablation with HF vs. medical therapy with HF, Latin American with warfarin use vs. non-Latin American with warfarin use, Latin American with NOAC use vs. non-Latin American with NOAC, Latin American with warfarin and NOAC use vs. non-Latin American with warfarin and NOAC use, and paroxysmal AF vs. persistent AF were graded as high-quality evidence given the RCT study design. Besides, DOAC with polypharmacy vs. warfarin with polypharmacy, DOAC without amiodarone vs. warfarin without amiodarone, DOAC without P-glycoprotein inhibitor vs. warfarin without P-glycoprotein inhibitor, NOAC (no previous stroke or TIA) vs. warfarin (no previous stroke or TIA), NOAC (non-Latin American) vs. warfarin (no previous stroke or TIA), sotalol vs. placebo or no treatment, sotalol (PeAF) vs. placebo or no treatment (PeAF), catheter ablation (HF and non-HF) vs. medical therapy (HF and non-HF), and SGLT2 inhibitors vs. placebo were classified as moderate-quality evidence among the significant risk factors. The remaining risk factors were assessed as very low- to low-quality evidence due to observational study design, risk of bias, inconsistency, or imprecision. Detailed GRADE evaluation results are shown in Supplementary Table S4. Sensitivity analyses conducted for outcomes rated as high-quality confirmed the stability of the associations, with no changes in direction or statistical significance.

## 4 Discussion

### 4.1 Principal findings and possible explanations

AF is the most common sustained arrhythmia globally, with rising incidence linked to aging and increasing prevalence of hypertension, diabetes, and obesity. AF is strongly associated with elevated mortality, driven by risk factors such as advanced age, heart failure, diabetes, chronic kidney disease, and prior stroke (62–64). Risk stratification tools like CHA_2_DS_2_-VASc help guide anticoagulation decisions (63, 65), and lifestyle factors also contribute to disease progression (64). This study aims to explore the multifaceted relationship between AF and mortality, focusing on the clinical significance of various risk factors and recent advancements in treatment and prevention strategies.

A total of 216 unique risk factors were found in this umbrella review, comprising 86 with statistically significant associations and 130 without. Among these, 32 harmful and 54 beneficial associations were found to be statistically significant. According to the GRADE evidence evaluation criteria, most results were classified as low or very low quality evidence. Among the significant risk factors, 11 were rated as high-quality evidence and 9 were classified as moderate-quality evidence. The vast majority of mortality-related risk factors in AF were associated with anticoagulant use.

### 4.2 Anticoagulants are an important cause of decreased mortality in AF patients

NOAC directly inhibit thrombin or factor Xa, whereas warfarin acts indirectly on vitamin K-dependent clotting factors and requires routine INR monitoring (66). However, most mortality benefits observed in this umbrella review were supported by low- to very-low-certainty evidence, with only a few comparisons reaching high certainty. Clinically, these findings support a preference for NOAC over warfarin in most AF patients, particularly those at higher thromboembolic or bleeding risk (28, 67). Nevertheless, residual confounding is likely, as NOAC users may differ systematically from warfarin users in healthcare access, adherence, and baseline risk profiles. The apparent superiority of apixaban or dabigatran over rivaroxaban requires confirmation in head-to-head trials (28). Individualised prescribing remains essential, taking into account renal function, comorbidities, and concomitant medications (68). Studies have already integrated the timing of atrial fibrillation onset with machine learning to predict stroke, bleeding, and all-cause mortality during anticoagulant therapy (69). Future research should include well-designed randomised comparisons between individual NOAC and studies specifically targeting elderly, frail, and multi-morbid populations to clarify whether observed associations reflect true treatment effects or residual confounding.

### 4.3 Some kinds of antiarrhythmics increase the mortality to some extent in AF patients

This study found that digoxin was associated with higher all-cause, cardiovascular, and sudden cardiac mortality. Mechanistically, digoxin inhibits sodium–potassium ATPase, increasing intracellular calcium and myocardial contractility, while also enhancing vagal tone to slow atrioventricular conduction (70). However, these benefits are offset by proarrhythmic effects, particularly with electrolyte disturbances. Dronedarone and sotalol were also associated with increased mortality, partly driven by interactions with digoxin and inherent proarrhythmic risks (34, 37). The evidence for these associations was predominantly low-to-very-low quality, with residual confounding highly likely—patients receiving digoxin or class III agents often have more advanced disease and comorbidities, making it difficult to isolate drug-specific effects. Clinically, these findings do not preclude use but emphasise caution: digoxin should be used at low doses with close monitoring, and dronedarone should be avoided when concomitant digoxin therapy is indicated. It remains to be determined, through well-controlled prospective studies, whether these associations reflect drug toxicity, disease severity, or both.

### 4.4 Different kinds of comorbidity could increase the risk of death in AF patients

Comorbidities such as diabetes, COPD, peripheral artery disease, anaemia, LVH, cancer, and frailty are each associated with increased mortality in AF patients, though mechanistic pathways vary, and previous studies on risk models have shown that AF risk stratification is associated with these risk factors (71). Diabetes may promote atrial fibrosis via oxidative stress and inflammation (72); COPD contributes through systemic inflammation and pulmonary hypertension (73, 74); peripheral artery disease reflects high atherosclerotic burden (75); anaemia and LVH cause haemodynamic strain (76); while cancer and frailty add hypercoagulability, cardiotoxicity, and treatment non-adherence (77, 78). Despite these plausible mechanisms, most evidence is low-to-very-low quality, derived from observational studies with substantial residual confounding—multimorbid patients tend to be older and frailer, making independent effects difficult to isolate. Clinically, these findings underscore the need for holistic management, optimising glycaemic control, respiratory function, and cardiovascular risk factors, while cautiously balancing anticoagulation benefits against bleeding risks, especially in anaemic or frail individuals. Anticoagulation decisions should be individualised, considering both thrombotic and bleeding risks, which are amplified in these comorbid groups. To guide tailored interventions in multimorbid populations, prospective studies with rigorous confounder adjustment and standardised definitions are essential.

### 4.5 Demographic characteristics had an impact on mortality in patients with AF

This study found that high BMI was associated with lower mortality, whereas low BMI, smoking, advanced age, and Latino ethnicity were associated with higher mortality. The obesity paradox—better outcomes in obese AF patients—may be explained by greater metabolic reserve, altered drug pharmacokinetics, and potential haemodynamic advantages (79). However, this paradoxical association is likely confounded by disease severity, as unintentional weight loss from advanced illness can bias estimates. Additionally, smoking promotes endothelial dysfunction, oxidative stress, and atherosclerosis, contributing to arrhythmogenesis (80); ageing drives left atrial fibrosis, diastolic dysfunction, and electrical remodelling (81); and Latino patients face higher mortality, potentially due to disparities in healthcare access, anticoagulation quality, and socioeconomic factors (23, 82).

Critically, most evidence underlying these associations was low-to-very-low quality, with residual confounding highly probable—particularly for BMI, where comorbidities and frailty influence weight. The high-certainty finding for Latino ethnicity highlights healthcare inequities rather than biological differences (23). Clinically, smoking cessation should be prioritised, and anticoagulation management in elderly and Latino populations requires individualised approaches addressing adherence and follow-up. Addressing these uncertainties requires prospective studies with rigorous confounder adjustment, investigation of social determinants of health, and stratified analyses by disease severity to clarify the nature of these associations.

### 4.6 Cardiac intervention surgery can reduce the risk of death in AF patients

Catheter ablation was associated with reduced all-cause and cardiovascular mortality. Ablation delivers thermal energy to create lesions that eliminate arrhythmogenic foci. Evidence quality varied: high-certainty in specific subgroups (e.g., heart failure), but low-to-very-low in others (83). Clinically, ablation is preferred for symptomatic or drug-intolerant patients. However, residual confounding exists, as ablation candidates are often younger with fewer comorbidities. Future trials should compare ablation with contemporary medical therapy in unselected populations.

Left atrial appendage occlusion (LAAO) also showed associations with lower mortality. LAAO prevents thromboembolism by excluding the appendage, the main source of clots in nonvalvular AF (84). All supporting evidence was low-to-very-low quality, derived from observational studies with significant selection bias—LAAO patients typically have high bleeding risk or contraindications to anticoagulation. This procedure offers an alternative for such patients, but its mortality benefit should not be generalised. Head-to-head trials vs. DOAC are urgently needed to confirm efficacy and safety.

### 4.7 Other risk factors affect mortality in patients with AF

This study found that SGLT2 inhibitors, statins, paroxysmal AF (vs. persistent/non-paroxysmal), and early rhythm control were each associated with lower mortality. SGLT2 inhibitors may confer cardioprotection through metabolic modulation and reduced atrial cardiomyocyte excitability; statins exert pleiotropic anti-inflammatory and lipid-lowering effects (85); and early rhythm control improves outcomes by preventing adverse remodelling (86).

The evidence quality varied: SGLT2 inhibitors reached moderate certainty (87), AF type reached high certainty for paroxysmal vs. persistent (88), while statins and early rhythm control were supported by low-to-very-low certainty data (85, 86). Residual confounding is a major concern—patients receiving these therapies often have better healthcare access and fewer comorbidities. Clinically, these findings support considering SGLT2 inhibitors and statins as adjunctive therapies, and favour early rhythm control in suitable candidates. However, causality cannot be inferred. Future trials should assess whether these associations reflect true treatment effects or selection biases.

## 5 Limitations and strengths

Several limitations should be acknowledged. First, overlap of primary studies across different meta-analyses may have led to double-counting of the same data, potentially inflating the precision of pooled estimates. Second, reliance on existing meta-analyses rather than original studies may have missed some primary research and inherited errors or biases from the original analyses. Third, the methodological quality of the included meta-analyses varied considerably, despite our use of AMSTAR, which may affect the reliability of certain findings. Fourth, definitions of mortality outcomes were not uniform across meta-analyses, complicating direct comparisons between studies. Fifth, heterogeneity in comparators, for example, different anticoagulant regimens or control groups, may have influenced the interpretation of treatment effects. Sixth, because most included studies were observational, causality cannot be inferred from the reported associations. Seventh, limiting our search to English language databases may have introduced language bias, potentially excluding relevant studies published in other languages. Despite these limitations, we performed systematic searches, independent screening, and data extraction, recalculated effect sizes using random or fixed effects models, and assessed heterogeneity and publication bias. We also evaluated methodological quality and certainty of evidence using AMSTAR and GRADE, respectively, to strengthen confidence in our conclusions.

## 6 Conclusion

This umbrella review identified 216 risk factors, with 86 showing significant associations and 130 non-significant. Among the significant ones, 32 were harmful and 54 protective. However, most evidence was graded as low or very low quality by GRADE; only 11 factors had high-quality evidence and 9 had moderate-quality evidence. Consequently, all findings should be interpreted as associations rather than causal relationships. Treatment effects must be understood within specific comparator contexts. The protective effect of high BMI (the obesity paradox) and the increased risk associated with Latino ethnicity require cautious interpretation, as they may be confounded by residual factors or healthcare disparities. With these caveats, increased mortality was associated with warfarin/VKA (vs. NOAC), digoxin, dronedarone, sotalol, comorbidities, low BMI, older age, smoking, Latino ethnicity, and persistent AF, while NOAC, surgery, high BMI, SGLT2 inhibitors, statins, and early rate control were linked to reduced all-cause mortality. These findings highlight the need for higher-quality comparative studies and careful consideration of confounding and comparative contexts.

## Data Availability

The raw data supporting the conclusions of this article will be made available by the authors, without undue reservation.

## References

[1] Lip GY Fauchier L Freedman SB Van Gelder I Natale A Gianni C. Atrial fibrillation. Nat Rev Dis Prim. (2016) 2:16016. 10.1038/nrdp.2016.16 27159789

[2] Zhang J Johnsen SP Guo Y Lip GYH. Epidemiology of atrial fibrillation: geographic/ecological risk factors, age, sex, genetics. Card Electrophysiol Clin. (2021) 13(1):1–23. 10.1016/j.ccep.2020.10.010 33516388

[3] Page MJ Moher D Bossuyt PM Boutron I Hoffmann TC Mulrow CD. PRISMA 2020 explanation and elaboration: updated guidance and exemplars for reporting systematic reviews. Br Med J. (2021) 372:n160. 10.1136/bmj.n160 PMC8005925 33781993

[4] Aromataris E Stern C Lockwood C Barker TH Klugar M Jadotte Y. JBI series paper 2: tailored evidence synthesis approaches are required to answer diverse questions: a pragmatic evidence synthesis toolkit from JBI. J Clin Epidemiol. (2022) 150:196–202. 10.1016/j.jclinepi.2022.04.006 35429608

[5] Cumpston M Li T Page MJ Chandler J Welch VA Higgins JP. Updated guidance for trusted systematic reviews: a new edition of the cochrane handbook for systematic reviews of interventions. Cochrane Database Syst Rev. (2019) 10(10):Ed000142. 10.1002/14651858.ED000142 31643080 PMC10284251

[6] Huang Y Chen Z Chen B Li J Yuan X Li J. Dietary sugar consumption and health: umbrella review. Br Med J. (2023) 381:e071609. 10.1136/bmj-2022-071609 PMC10074550 37019448

[7] Singh M Wambua S Lee SI Okoth K Wang Z Fazla F. Autoimmune diseases and adverse pregnancy outcomes: an umbrella review. Lancet. (2023) 402(Suppl 1):S84. 10.1016/S0140-6736(23)02128-1 37997130

[8] Ferguson T Olds T Curtis R Blake H Crozier AJ Dankiw K. Effectiveness of wearable activity trackers to increase physical activity and improve health: a systematic review of systematic reviews and meta-analyses. Lancet Digit Health. (2022) 4(8):e615–26. 10.1016/S2589-7500(22)00111-X 35868813

[9] General Practice and the Community: Research on health service, quality improvements and training. Selected abstracts from the EGPRN Meeting in Vigo, Spain, 17–20 October 2019 Abstracts. Eur J Gen Pract. (2020) 26(1):42–50. 10.1080/13814788.2020.1719994

[10] Abubakar H Osman M Akintoye E Subahi A Osman K Abidov A. Intra-thoracic impedance and the onset of atrial and ventricular tachyarrhythmias: a meta-analysis. Int J Cardiol. (2018) 258:144–50. 10.1016/j.ijcard.2017.12.092 29544921

[11] Aguilar MI Hart R Pearce LA. Oral anticoagulants versus antiplatelet therapy for preventing stroke in patients with non-valvular atrial fibrillation and no history of stroke or transient ischemic attacks. Cochrane Database Syst Rev. (2007) 3:Cd006186. 10.1002/14651858.CD006186 PMC13591242 17636831

[12] Guyatt G Hultcrantz M Agoritsas T Iorio A Vandvik PO Montori VM. Why core GRADE is needed: introduction to a new series in the BMJ. Br Med J. (2025) 389:e081902. 10.1136/bmj-2024-081902 40233981

[13] Hultcrantz M Schünemann HJ Mustafa RA Rind DM Murad MH Mayer M. GRADE certainty ratings: thresholds rather than categories of contextualization (GRADE guidance 41). Ann Intern Med. (2025) 178(8):1183–6. 10.7326/ANNALS-25-00548 40587852

[14] Li X Meng XR Timofeeva M Tzoulaki I Tsilidis KK Ioannidis PA. Serum uric acid levels and multiple health outcomes: umbrella review of evidence from observational studies, randomised controlled trials, and Mendelian randomisation studies. Br Med J. (2017) 357:j2376. 10.1136/bmj.j2376 PMC5461476 28592419

[15] Chatterjee NA Giulianini F Geelhoed B Lunetta KL Misialek JR Niemeijer MN. Genetic obesity and the risk of atrial fibrillation causal estimates from Mendelian randomization. Circulation. (2017) 135(8):741–54. 10.1161/CIRCULATIONAHA.116.024921 27974350 PMC5322057

[16] Egger M Davey Smith G Schneider M Minder C. Bias in meta-analysis detected by a simple, graphical test. Br Med J. (1997) 315(7109):629–34. 10.1136/bmj.315.7109.629 PMC2127453 9310563

[17] Patel SM Braunwald E Steffel J Boriani G Palazzolo MG Antman EM. Efficacy and safety of non-vitamin-K antagonist oral anticoagulants versus warfarin across the spectrum of body mass index and body weight: an individual patient data meta-analysis of 4 randomized clinical trials of patients with atrial fibrillation. Circulation. (2024) 149(12):932–43. 10.1161/CIRCULATIONAHA.123.066279 38264923 PMC12560831

[18] Gómez-Outes A Terleira-Fernández AI Calvo-Rojas G Suárez-Gea ML Vargas-Castrillón E. Dabigatran, rivaroxaban, or apixaban versus warfarin in patients with nonvalvular atrial fibrillation: a systematic review and meta-analysis of subgroups. Thrombosis. (2013) 2013:640723–18. 10.1155/2013/640723 PMC3885278 24455237

[19] Bennaghmouch N De Veer A Bode K Mahmoodi BK Dewilde WJM Lip GYH. Efficacy and safety of the use of non-vitamin K antagonist oral anticoagulants in patients with nonvalvular atrial fibrillation and concomitant aspirin therapy: a meta-analysis of randomized trials. Circulation. (2018) 137(11):1117–29. 10.1161/CIRCULATIONAHA.117.028513 29101289

[20] Liu GJ Wang YF Chen PY Chang W Tu ML Chang LY. The efficacy and safety of novel oral anticoagulants for the preventive treatment in atrial fibrillation patients: a systematic review and meta-analysis. Drug Deliv. (2014) 21(6):436–52. 10.3109/10717544.2013.873500 24400656

[21] Garg J Chaudhary R Krishnamoorthy P Palaniswamy C Shah N Bozorgnia B. Safety and efficacy of oral factor-Xa inhibitors versus vitamin K antagonist in patients with non-valvular atrial fibrillation: meta-analysis of phase II and III randomized controlled trials. Int J Cardiol. (2016) 218:235–9. 10.1016/j.ijcard.2016.05.059 27236121

[22] Kim IS Kim HJ Yu HT Kim TH Uhm JS Kim JY. Non-vitamin K antagonist oral anticoagulants with amiodarone, P-glycoprotein inhibitors, or polypharmacy in patients with atrial fibrillation: systematic review and meta-analysis. J Cardiol. (2019) 73(6):515–21. 10.1016/j.jjcc.2018.12.018 30770140

[23] Su ZY Zhang H He WF Ma JY Zeng JQ Jiang XH. Meta-analysis of the efficacy and safety of non-vitamin K antagonist oral anticoagulants with warfarin in Latin American patients with atrial fibrillation. Medicine (Baltimore). (2020) 99(18):e19542. 10.1097/MD.0000000000019542 32358343 PMC7440306

[24] Hart RG Pearce LA Aguilar MI. Meta-analysis: antithrombotic therapy to prevent stroke in patients who have nonvalvular atrial fibrillation. Ann Intern Med. (2007) 146(12):857–67. 10.7326/0003-4819-146-12-200706190-00007 17577005

[25] Waranugraha Y Rizal A Syaban MFR Faratisha IFD Erwan NE Yunita KC. Direct comparison of non-vitamin K antagonist oral anticoagulant versus warfarin for stroke prevention in non-valvular atrial fibrillation: a systematic review and meta-analysis of real-world evidences. Egypt Heart J. (2021) 73(1):70. 10.1186/s43044-021-00194-1 34379219 PMC8357866

[26] Blann AD Skjøth F Rasmussen LH Larsen TB Lip GY. Edoxaban versus placebo, aspirin, or aspirin plus clopidogrel for stroke prevention in atrial fibrillation. An indirect comparison analysis. Thromb Haemost. (2015) 114(2):403–9. 10.1160/TH15-05-0383 26062437

[27] Aronow WS Shamliyan TA. Comparative clinical outcomes of edoxaban in adults with nonvalvular atrial fibrillation. Am J Ther. (2020) 27(3):e270–85. 10.1097/MJT.0000000000000848 30277905

[28] Buckley BJR Lane DA Calvert P Zhang JQ Gent D Mullins CD. Effectiveness and safety of apixaban in over 3.9 million people with atrial fibrillation: a systematic review and meta-analysis. J Clin Med. (2022) 11(13):3788. 10.3390/jcm11133788 35807073 PMC9267894

[29] Escobar C Barrios V Lip GYH Amin AN Auladell-Rispau A Santero M. Effectiveness and safety of dabigatran compared to vitamin K antagonists in non-Asian patients with atrial fibrillation: a systematic review and meta-analysis. Clin Drug Investig. (2021) 41(11):941–53. 10.1007/s40261-021-01091-w 34643934

[30] Bai Y Deng H Shantsila A Lip GY. Rivaroxaban versus dabigatran or warfarin in real-world studies of stroke prevention in atrial fibrillation: systematic review and meta-analysis. Stroke. (2017) 48(4):970–6. 10.1161/STROKEAHA.116.016275 28213573

[31] Xu ZQ Xu ZH Zhang N. Comprehensive systematic review and meta-analysis on anticoagulants and aspirin for stroke prevention in non-valvular atrial fibrillation patients. Eur Rev Med Pharmacol Sci. (2023) 27(22):11002–12. 10.37766/inplasy2023.9.0089 38039031

[32] Hannon N Arsava EM Audebert HJ Ay H Crowe M Chróinín DN. Antithrombotic treatment at onset of stroke with atrial fibrillation, functional outcome, and fatality: a systematic review and meta-analysis. Int J Stroke. (2015) 10(6):808–14. 10.1111/ijs.12473 25808552

[33] Abdool M Kunutsor SK Khunti K Seidu S. Does the presence of diabetes mellitus confer an increased risk of stroke in patients with atrial fibrillation on direct oral anticoagulants? A systematic review and meta-analysis. Diabetes Metab Syndr. (2020) 14(6):1725–33. 10.1016/j.dsx.2020.08.038 32916556

[34] Valembois L Audureau E Takeda A Jarzebowski W Belmin J Lafuente-Lafuente C. Antiarrhythmics for maintaining sinus rhythm after cardioversion of atrial fibrillation. Cochrane Database Syst Rev. (2019) 2019(9):CD005049. 10.1002/14651858.CD005049.pub5 PMC6738133 31483500

[35] Trongtorsak A Kewcharoen J Saowapa S Polpichai N Thangjui S Navaravong L. Comparison of mortality rates among rate control agents in patients with atrial fibrillation: a systematic review and meta-analysis. J Cardiovasc Med. (2022) 23(1):e39–41. 10.2459/JCM.0000000000001283 34860198

[36] Wang XX Luo Y Xu D Zhao K. Effect of digoxin therapy on mortality in patients with atrial fibrillation: an updated meta-analysis. Front Cardiovasc Med. (2021) 8:731135. 10.3389/fcvm.2021.731135 34660731 PMC8517124

[37] Boriani G Blomström-Lundqvist C Hohnloser SH Bergfeldt L Botto GL Capucci A. Safety and efficacy of dronedarone from clinical trials to real-world evidence: implications for its use in atrial fibrillation. Europace. (2019) 21(12):1764–75. 10.1093/europace/euz193 31324921

[38] Xu J Sun YM Gong DD Fan Y. Impact of preexisting diabetes mellitus on cardiovascular and all-cause mortality in patients with atrial fibrillation: a meta-analysis. Front Endocrinol (Lausanne). (2022) 13:921159. 10.3389/fendo.2022.921159 35979438 PMC9376236

[39] Tu SJ Hanna-Rivero N Elliott AD Clarke N Huang S Pitman BM. Associations of anemia with stroke, bleeding, and mortality in atrial fibrillation: a systematic review and meta-analyses. J Cardiovasc Electrophysiol. (2021) 32(3):686–94. 10.1111/jce.14898 33476452

[40] Balomenakis C Papazoglou AS Vlachopoulou D Kartas A Moysidis DV Vouloagkas I. Risk of arterial thromboembolism, bleeding and mortality in atrial fibrillation patients with comorbid cancer: a systematic review and meta-analysis. Hellenic J Cardiol. (2023) 74:65–73. 10.1016/j.hjc.2023.06.005 37414144

[41] Romiti GF Corica B Pipitone E Vitolo M Raparelli V Basili S. Prevalence, management and impact of chronic obstructive pulmonary disease in atrial fibrillation: a systematic review and meta-analysis of 4,200,000 patients. Eur Heart J. (2021) 42(35):3541–54. 10.1093/eurheartj/ehab453 34333599

[42] He L He R Huang JB Zou C Fan Y. Impact of frailty on all-cause mortality and major bleeding in patients with atrial fibrillation: a meta-analysis. Ageing Res Rev. (2022) 73:101527. 10.1016/j.arr.2021.101527 34839042

[43] Xiang H Xue Y Chen Z Yu Y Peng Y Wang J. The association between left ventricular hypertrophy and the occurrence and prognosis of atrial fibrillation: a meta-analysis. Front Cardiovasc Med. (2021) 8:639993. 10.3389/fcvm.2021.639993 34395549 PMC8362884

[44] Zhu JF Tan XW Zhou JZ. Peripheral artery disease and clinical outcomes in patients with atrial fibrillation: a systematic review and meta-analysis. Clin Cardiol. (2021) 44(8):1050–7. 10.1002/clc.23678 34170015 PMC8364730

[45] Liu X Guo L Xiao K Zhu W Liu M Wan R. The obesity paradox for outcomes in atrial fibrillation: evidence from an exposure-effect analysis of prospective studies. Obes Rev. (2020) 21(3):e12970. 10.1111/obr.12970 31849187

[46] Zhu WG Wan R Liu FW Hu JZ Huang L Li JX. Relation of body mass index with adverse outcomes among patients with atrial fibrillation: a meta-analysis and systematic review. J Am Heart Assoc. (2016) 5(9):e004006. 10.1161/JAHA.116.004006 27613773 PMC5079045

[47] Grymonprez M Capiau A De Backer TL Steurbaut S Boussery K Lahousse L. The impact of underweight and obesity on outcomes in anticoagulated patients with atrial fibrillation: a systematic review and meta-analysis on the obesity paradox. Clin Cardiol. (2021) 44(5):599–608. 10.1002/clc.23593 33769583 PMC8119828

[48] Zhu W Guo L Hong K. Relationship between smoking and adverse outcomes in patients with atrial fibrillation: a meta-analysis and systematic review. Int J Cardiol. (2016) 222:289–94. 10.1016/j.ijcard.2016.07.220 27500755

[49] Li F Zhang L Wu LD Zhang ZY Liu HH Zhang ZY. Do elderly patients with atrial fibrillation have comparable ablation outcomes compared to younger ones? Evidence from pooled clinical studies. J Clin Med. (2022) 11(15):4468. 10.3390/jcm11154468 35956085 PMC9369410

[50] Asad ZU Yousif A Khan MS Al-Khatib SM Stavrakis S. Catheter ablation versus medical therapy for atrial fibrillation A systematic review and meta-analysis of randomized controlled trials. Circ Arrhythm Electrophysiol. (2019) 12(9):e007414. 10.1161/CIRCEP.119.007414 31431051

[51] Zahoor MM Ullah SE Kidiavai HM Eltieb SAH Devi A Asif MA. Efficacy of ablation therapy on clinical outcomes in patients with atrial fibrillation: a systematic review and meta-analysis. Ann Med Surg. (2023) 85(9):4491–500. 10.1097/MS9.0000000000000985 PMC10473299 37663739

[52] Shaik TA Haseeb M Faisal S Obeidat K Salam O Karedath J. Impact of catheter ablation on long-term outcomes in patients with atrial fibrillation: a meta-analysis. Cureus J Med Sci. (2022) 14(9):e29202. 10.7759/cureus.29202 PMC9569152 36259007

[53] Franchin L Piroli F Demola P Mantovani F Iannaccone M Manfredi R. Efficacy and safety of left atrial appendage closure compared with oral anticoagulation in atrial fibrillation: a meta-analysis of randomized controlled trials and propensity-matched studies. Front Cardiovasc Med. (2023) 10:1212161. 10.3389/fcvm.2023.1212161 37829693 PMC10565038

[54] Abdelfattah OM Sayed A Munir M Almotawally S Wilson K Gad MM. Meta-analysis comparing left atrial appendage occlusion, direct oral anticoagulants, and warfarin for nonvalvular atrial fibrillation. Am J Cardiol. (2023) 186:117–25. 10.1016/j.amjcard.2022.08.012

[55] Prasad RM Saleh Y Al-Abcha A Abdelkarim O Abdelfattah OM Abdelnabi M. Left atrial appendage closure during cardiac surgery for atrial fibrillation: a meta-analysis. Cardiovasc Revasc Med. (2022) 40:26–36. 10.1016/j.carrev.2021.11.002 34801420

[56] Zheng SY Lai YW Jiang C He L Zhao ZX Li WJ. Effect of SGLT2 inhibitors on cardiovascular events in patients with atrial fibrillation: a systematic review and meta-analysis of randomized controlled trials. Pace-Pacing Clin Electrophysiol. (2024) 47(1):58–65. 10.1111/pace.14880 38010824

[57] Pastori D Baratta F Di Rocco A Farcomeni A Del Ben M Angelico F. Statin use and mortality in atrial fibrillation: a systematic review and meta-analysis of 100,287 patients. Pharmacol Res. (2021) 165:105418. 10.1016/j.phrs.2021.105418 33450384

[58] Eun MY Jung JM Choi KH Seo WK. Statin effects in atrial fibrillation-related stroke: a systematic review and meta-analysis. Front Neurol. (2020) 11:589684. 10.3389/fneur.2020.589684 33162931 PMC7581731

[59] Zhang WF Xiong YW Yu LL Xiong AZ Bao HH Cheng XS. Meta-analysis of stroke and bleeding risk in patients with various atrial fibrillation patterns receiving oral anticoagulants. Am J Cardiol. (2019) 123(6):922–8. 10.1016/j.amjcard.2018.11.055 30691678

[60] Mentel A Quinn TJ Cameron AC Lees KR Abdul-Rahim AH. The impact of atrial fibrillation type on the risks of thromboembolic recurrence, mortality and major haemorrhage in patients with previous stroke: a systematic review and meta-analysis of observational studies. Eur Stroke J. (2020) 5(2):155–68. 10.1177/2396987319896674 32637649 PMC7313372

[61] Han SJ Jia RK Cen ZF Guo R Zhao SY Bai YX. Early rhythm control vs. Rate control in atrial fibrillation: a systematic review and meta-analysis. Front Cardiovasc Med. (2023) 10:978637. 10.3389/fcvm.2023.978637 36815025 PMC9939510

[62] Desai R Hanna B Singh S Omar A Deshmukh A Kumar G. Trends and outcomes in sepsis hospitalizations with and without atrial fibrillation: a nationwide inpatient analysis. Crit Care Med. (2019) 47(8):e630–8. 10.1097/CCM.0000000000003806 31094740

[63] Marijon E Le Heuzey JY Connolly S Yang S Pogue J Brueckmann M. Causes of death and influencing factors in patients with atrial fibrillation: a competing-risk analysis from the randomized evaluation of long-term anticoagulant therapy study. Circulation. (2013) 128(20):2192–201. 10.1161/CIRCULATIONAHA.112.000491 24016454

[64] Lai Y Yoshimura H Zakkak N Marijon E Chahal A Lip GYH. Causes of death in patients with atrial fibrillation in the UK: a nationwide electronic health record study. Eur Heart J Open. (2024) 5(1):oeae103. 10.1093/ehjopen/oeae103 39790490 PMC11711847

[65] Pregaldini F Çelik M Mosbahi S Barmettler S Praz F Reineke D. Perioperative and mid-term outcomes of mitral valve surgery with and without concomitant surgical ablation for atrial fibrillation: a retrospective analysis. Interdiscip Cardiovasc Thorac Surg. (2024) 39(2):ivae144. 10.1093/icvts/ivae144 39083003 PMC11315649

[66] Calabrò P Gragnano F Cesaro A Marsico F Pariggiano I Patti G. Non-vitamin K antagonist oral anticoagulants in patients with atrial fibrillation and atrial thrombosis: an appraisal of current evidence. Arch Cardiovasc Dis. (2020) 113(10):642–51. 10.1016/j.acvd.2020.03.019 32712202

[67] Cohen AT Sah J Dhamane AD Hines DM Lee T Rosenblatt L. Effectiveness and safety of apixaban vs warfarin in patients with venous thromboembolism with risk factors for bleeding or for recurrences. Adv Ther. (2023) 40(4):1705–35. 10.1007/s12325-023-02440-1 36811795 PMC10070226

[68] Kwon S Lee SR Choi EK Lee E Jung JH Han KD. Non-vitamin K antagonist oral anticoagulants in patients with atrial fibrillation and prior gastrointestinal bleeding. Stroke. (2021) 52(2):511–20. 10.1161/STROKEAHA.120.030761 33412904

[69] Joddrell M El-Bouri W Harrison SL Huisman MV Lip GYH Zheng Y. Machine learning for outcome prediction in patients with non-valvular atrial fibrillation from the GLORIA-AF registry. Sci Rep. (2024) 14(1):27088. 10.1038/s41598-024-78120-z 39511367 PMC11544011

[70] Miró Ò Martín Mojarro E Lopez-Ayala P Llorens P Gil V Alquézar-Arbé A. Association of intravenous digoxin use in acute heart failure with rapid atrial fibrillation and short-term mortality according to patient age, renal function, and serum potassium. Eur J Emerg Med. (2024) 31(5):347–55. 10.1097/MEJ.0000000000001153 38985840

[71] Yoshimura H Schmidt AF Lip GYH Providencia R. Predicting common cardiovascular and non-cardiovascular outcomes after new-onset atrial fibrillation: an analysis from a UK nationwide primary-secondary care linkage dataset. Eur Heart J Open. (2026) 6(3):oeag103. 10.1093/ehjopen/oeag103 42327685 PMC13282438

[72] Lee Y Cha SJ Park JH Shin JH Lim YH Park HC. Association between insulin resistance and risk of atrial fibrillation in non-diabetics. Eur J Prev Cardiol. (2020) 27(18):1934–41. 10.1177/2047487320908706 32122201

[73] Ye J Yao P Shi X Yu X. A systematic literature review and meta-analysis on the impact of COPD on atrial fibrillation patient outcome. Heart Lung. (2022) 51:67–74. 10.1016/j.hrtlng.2021.09.001 34740082

[74] Warming PE Garcia R Hansen CJ Simons SO Torp-Pedersen C Linz D. Atrial fibrillation and chronic obstructive pulmonary disease: diagnostic sequence and mortality risk, European heart journal. Qual Care Clin Outcomes. (2023) 9(2):128–34. 10.1093/ehjqcco/qcac059 36069895

[75] Vitalis A Nirantharakumar K Thayakaran R Vohra RK Kay M Shantsila A. The impact of atrial fibrillation on outcomes of peripheral arterial disease: analysis of routinely collected primary care data. Am J Med. (2022) 135(4):488–92. 10.1016/j.amjmed.2021.10.021 34793748

[76] Hijazi Z Verdecchia P Oldgren J Andersson U Reboldi G Pasquale GD. Cardiac biomarkers and left ventricular hypertrophy in relation to outcomes in patients with atrial fibrillation: experiences from the RE—LY trial. J Am Heart Assoc. (2019) 8(2):e010107. 10.1161/JAHA.118.010107 30651032 PMC6497355

[77] Hajjar LA Fonseca SMR Machado TIV. Atrial fibrillation and cancer. Front Cardiovasc Med. (2021) 8:590768. 10.3389/fcvm.2021.590768 34336939 PMC8319502

[78] Nakamaru R Nishimura S Kumamaru H Shoji S Nakatani E Yamamoto H. Trajectories of frailty and clinical outcomes in older adults with atrial fibrillation: insights from the Shizuoka Kokuho database, Circ Cardiovasc Qual Outcomes. (2024) 17(8):e010642. 10.1161/CIRCOUTCOMES.123.010642 39167767

[79] Lima Filho AIN Do Rego Barros MC De Barros Guimarães AA Celestino Sobral Filho D. Obesity paradox in atrial fibrillation and its relation with the new oral anticoagulants. Curr Cardiol Rev. (2022) 18(5):8–10. 10.2174/1573403X18666220324111343 35331095 PMC9896420

[80] Higashi Y. Smoking cessation and vascular endothelial function. Hypertens Res. (2023) 46(12):2670–8. 10.1038/s41440-023-01455-z 37828134 PMC10695829

[81] He JK Jiang XX Dai SY Xiao H Zhu QQ Jie Y. *β*-hydroxybutyrate and citrate synthase as potential diagnostic biomarkers in aging-related atrial fibrillation. J Cardiovasc Transl Res. (2025) 18(1):133–45. 10.1007/s12265-024-10569-9 39499445 PMC11885335

[82] Essien UR Kim N Hausmann LRM Mor MK Good CB Magnani JW. Disparities in anticoagulant therapy initiation for incident atrial fibrillation by race/ethnicity among patients in the veterans health administration system. JAMA Netw. Open. (2021) 4(7):e2114234. 10.1001/jamanetworkopen.2021.14234 34319358 PMC8319757

[83] Messori A Bartoli L Ferracane E Trippoli S. Medical therapy, radiofrequency ablation or cryoballoon ablation as first-line treatment for paroxysmal atrial fibrillation: interpreting efficacy through restricted mean survival time and network meta-analysis. Rev Cardiovasc Med. (2021) 22(3):557–61. 10.31083/j.rcm2203067 34565059

[84] Whitlock RP Belley-Côté EP. Concomitant surgical left atrial appendage occlusion: a review. Curr Cardiol Rep. (2022) 24(7):823–8. 10.1007/s11886-022-01705-5 35488947

[85] Huang TC Yap LH Chen CY Lin HW Lin SH Li YH. Long-term statin use is associated with reduced rates of adverse events in patients with newly diagnosed atrial fibrillation. J Am Heart Assoc. (2024) 13(24):e035827. 10.1161/JAHA.124.035827 39673286 PMC11935556

[86] Kim D Yang PS You SC Sung JH Jang E Yu HT. Treatment timing and the effects of rhythm control strategy in patients with atrial fibrillation: nationwide cohort study. Br Med J. (2021) 373:n991. 10.1136/bmj.n991 PMC8111568 33975876

[87] Paasche A Wiedmann F Kraft M Seibertz F Herlt V Blochberger PL. Acute antiarrhythmic effects of SGLT2 inhibitors-dapagliflozin lowers the excitability of atrial cardiomyocytes. Basic Res Cardiol. (2024) 119(1):93–112. 10.1007/s00395-023-01022-0 38170280 PMC10837223

[88] De With RR Marcos EG Dudink E Spronk HM Crijns H Rienstra M. Atrial fibrillation progression risk factors and associated cardiovascular outcome in well-phenotyped patients: data from the AF-RISK study. Europace. (2020) 22(3):352–60. 10.1093/europace/euz339 31865391

