# Supplementary material for: Factors associated with mortality in patients with atrial fibrillation: an umbrella review of systematic reviews and meta-analyses
Source: Front Cardiovasc Med. 2026 Oct 13;13:1877770. doi: 10.3389/fcvm.2026.1877770 (PMC13710006; doi:10.3389/fcvm.2026.1877770)
Supplement: Supplementary file 1 [file Table1.docx]

TableS1 Literature search strategy

Pubmed-1724 (May 2025)

(((("Atrial Fibrillation"[Mesh]) OR (((((((((Atrial Fibrillations) OR (Auricular Fibrillation)) OR (Auricular Fibrillations)) OR (Persistent Atrial Fibrillation)) OR (Persistent Atrial Fibrillations)) OR (Familial Atrial Fibrillation)) OR (Familial Atrial Fibrillations)) OR (Paroxysmal Atrial Fibrillation)) OR (Paroxysmal Atrial Fibrillations))) AND (("Mortality"[Mesh]) OR ((((((((((((((((((((((((Mortalities) OR (Mortality Rate)) OR (Mortality Rates)) OR (Death Rate)) OR (Death Rates)) OR (Case Fatality Rate)) OR (Case Fatality Rates)) OR (CFR Case Fatality Rate)) OR (Crude Death Rate)) OR (Crude Death Rates)) OR (Crude Mortality Rate)) OR (Crude Mortality Rates)) OR (Mortality Declines)) OR (Mortality Decline)) OR (Mortality Determinants)) OR (Mortality Determinant)) OR (Differential Mortality)) OR (Differential Mortalities)) OR (Age-Specific Death Rate)) OR (Age-Specific Death Rates)) OR (Age Specific Death Rate)) OR (Excess Mortality)) OR (Excess Mortalities)) OR (Death)))) AND (systematic review OR meta-analysis)) AND (risk OR incidence OR predict)

Embase-1645 (May 2025)

((Atrial Fibrillation or (Atrial Fibrillations or Auricular Fibrillation or Auricular Fibrillations or Persistent Atrial Fibrillation or Persistent Atrial Fibrillations or Familial Atrial Fibrillation or Familial Atrial Fibrillations or Paroxysmal Atrial Fibrillation or Paroxysmal Atrial Fibrillations)) and (Mortality or (Mortalities or Mortality Rate or Mortality Rates or Death Rate or Death Rates or Case Fatality Rate or Case Fatality Rates or CFR Case Fatality Rate or Crude Death Rate or Crude Death Rates or Crude Mortality Rate or Crude Mortality Rates or Mortality Declines or Mortality Decline or Mortality Determinants or Mortality Determinant or Differential Mortality or Differential Mortalities or Age-Specific Death Rate or Age-Specific Death Rates or Age Specific Death Rate or Excess Mortality or Excess Mortalities or Death))and (systematicreview or meta-analysis) and (risk or incidence or predict)).ab.

Cochrane-325 (May 2025)

((Atrial Fibrillation or (Atrial Fibrillations or Auricular Fibrillation or Auricular Fibrillations or Persistent Atrial Fibrillation or Persistent Atrial Fibrillations or Familial Atrial Fibrillation or Familial Atrial Fibrillations or Paroxysmal Atrial Fibrillation or Paroxysmal Atrial Fibrillations)) and (Mortality or (Mortalities or Mortality Rate or Mortality Rates or Death Rate or Death Rates or Case Fatality Rate or Case Fatality Rates or CFR Case Fatality Rate or Crude Death Rate or Crude Death Rates or Crude Mortality Rate or Crude Mortality Rates or Mortality Declines or Mortality Decline or Mortality Determinants or Mortality Determinant or Differential Mortality or Differential Mortalities or Age-Specific Death Rate or Age-Specific Death Rates or Age Specific Death Rate or Excess Mortality or Excess Mortalities or Death)) and (systematic review or meta-analysis) and (risk or incidence or predict).af.

WOS-2042 (May 2025)

((((Atrial Fibrillation) OR (((((((((Atrial Fibrillations) OR (Auricular Fibrillation)) OR (Auricular Fibrillations)) OR (Persistent Atrial Fibrillation)) OR (Persistent Atrial Fibrillations)) OR (Familial Atrial Fibrillation)) OR (Familial Atrial Fibrillations)) OR (Paroxysmal Atrial Fibrillation)) OR (Paroxysmal Atrial Fibrillations))) AND ((Mortality) OR ((((((((((((((((((((((((Mortalities) OR (Mortality Rate)) OR (Mortality Rates)) OR (Death Rate)) OR (Death Rates)) OR (Case Fatality Rate)) OR (Case Fatality Rates)) OR (CFR Case Fatality Rate)) OR (Crude Death Rate)) OR (Crude Death Rates)) OR (Crude Mortality Rate)) OR (Crude Mortality Rates)) OR (Mortality Declines)) OR (Mortality Decline)) OR (Mortality Determinants)) OR (Mortality Determinant)) OR (Differential Mortality)) OR (Differential Mortalities)) OR (Age-Specific Death Rate)) OR (Age-Specific Death Rates)) OR (Age Specific Death Rate)) OR (Excess Mortality)) OR (Excess Mortalities)) OR (Death)))) AND (systematic review OR meta-analysis)) AND (risk OR incidence OR predict) (Topic)
