# Supplementary material for: Factors associated with mortality in patients with atrial fibrillation: an umbrella review of systematic reviews and meta-analyses
Source: Front Cardiovasc Med. 2026 Oct 13;13:1877770. doi: 10.3389/fcvm.2026.1877770 (PMC13710006; doi:10.3389/fcvm.2026.1877770)
Supplement: Supplementary file 2 [file Table2.docx]

Table S2. Effects of risk factors on mortality of patients with atrial fibrillation.

| Risk factor | Control | Outcomes | Total eligible MA | Included MA | Sample size | MA metric | Estimates [95% CI] |
| --- | --- | --- | --- | --- | --- | --- | --- |
|  |  |  |  |  | case/total |  |  |
| **Significant associations** |  |  |  |  |  |  |  |
| **Anticoagulants or Antiplatelets** |  |  |  |  |  |  |  |
| Adjusted-dose warfarin | Other antithrombotic treatment or placebo | All-cause mortality | 1 | Hart 2007 | 253/2900 | RR | 0.26 [0.03 to 0.43] |
| Apixaban | Rivaroxaban | Mortality | 6 | Buckley 2022 | 30305/878520 | RR | 0.83 [0.71 to 0.96] |
| Apixaban | Warfarin | All-cause mortality | 9 | Waranugraha 2021 | NA/404910 | HR | 0.69 [0.49 to 0.98] |
| Dabigatran 150mg | Warfarin | All-cause mortality | 5 | Escobar 2021 | NA | HR | 0.65 [0.58 to 0.73] |
| Dabigatran | Warfarin | All-cause mortality | 6 | Waranugraha 2021 | NA/606353 | HR | 0.67 [0.57 to 0.80] |
| DOAC (Diabetes) | VKA (Diabetes) | Cardiovascular mortality | 1 | Abdool 2020 | 715/13054 | RR | 0.84 [0.72 to 0.97] |
| DOAC (No Diabetes) | VKA (No Diabetes) | All-cause mortality | 1 | Abdool 2020 | 1546/22940 | RR | 0.89 [0.81 to 0.99] |
| DOAC | Warfarin | Mortality | 38 | Xu 2023 | 1518/23241 | OR | 0.49 [0.43 to 0.56] |
| DOAC (BMI 25-30) | Warfarin (BMI 25-30) | All-cause mortality | 2 | Patel 2024 | 1672/57866 | HR | 0.84 [0.76 to 0.92] |
| DOAC with polypharmacy | Warfarin with polypharmacy | Mortality | 1 | Kim 2019 | NA/20371 | RR | 0.91 [0.83 to 0.99] |
| (≥ 5 concomitant drugs) | (≥ 5 concomitant drugs) |  |  |  |  |  |  |
| DOAC without amiodarone | Warfarin without amiodarone | Mortality | 1 | Kim 2019 | NA/52137 | RR | 0.92 [0.86 to 0.98] |
| DOAC without P-glycoprotein inhibitor | Warfarin without P-glycoprotein inhibitor | Mortality | 1 | Kim 2019 | NA/46117 | RR | 0.91 [0.84 to 0.98] |
| Edoxaban 30mg | Aspirin | Mortality | 1 | Blann 2015 | 1860/14777 | RR | 0.82 [0.69 to 0.98] |
| Edoxaban 30mg | Placebo | Mortality | 1 | Blann 2015 | 837/8459 | RR | 0.64 [0.46 to 0.87] |
| Edoxaban 30mg | Warfarin | Mortality | 1 | Blann 2015 | 3577/47119 | RR | 0.88 [0.80 to 0.97] |
| Edoxaban 30mg with concomitant aspirin therapy | Warfarin with concomitant aspirin therapy | Vascular mortality | 1 | Bennaghmouch 2018 | 393/10261 | HR | 0.75 [0.59 to 0.96] |
| Edoxaban 60mg | Placebo | Mortality | 1 | Blann 2015 | 873/8460 | RR | 0.67 [0.49 to 0.92] |
| Edoxaban 60mg | Warfarin | Cardiovascular mortality | 4 | Aronow 2020 | NA/14799 | RR | 0.87 [0.78 to 0.97] |
| Edoxaban | Warfarin | All-cause mortality | 3 | Waranugraha 2021 | NA/32995 | HR | 0.52 [0.31 to 0.85] |
| HD-Rivaroxaban | HD-Dabigatran | Mortality | 1 | Bai 2017 | NA/147261 | HR | 1.27 [1.09 to 1.46] |
| LD-Rivaroxaban | LD-Dabigatran | Mortality | 1 | Bai 2017 | NA/147261 | HR | 1.17 [1.05 to 1.29] |
| NOAC (TTR < 65%) | Warfarin (TTR < 65%) | Mortality | 1 | Gómez-Outes 2013 | 1441/18114 | RR | 0.85 [0.76 to 0.93] |
| NOAC with concomitant aspirin therapy | Warfarin with concomitant aspirin therapy | Vascular mortality | 1 | Bennaghmouch 2018 | 872/21335 | HR | 0.85 [0.76 to 0.93] |
| NOAC (No previous stroke or TIA) | Warfarin (No previous stroke or TIA) | Mortality | 1 | Gómez-Outes 2013 | 2704/36050 | RR | 0.90 [0.84 to 0.97] |
| NOAC (non-Latin American) | Warfarin (non-Latin American) | All-cause mortality | 1 | Su 2020 | NA/62627 | RR | 0.90 [0.84 to 0.97] |
| Non−Ximelagatran NOAC | Control drugs | All-cause mortality | 1 | Liu 2014 | 4137/61088 | OR | 0.89 [0.84 to 0.95] |
| Oral Factor Xa inhibitors | VKA | All-cause mortality | 3 | Garg 2016 | 4101/56438 | OR | 0.88 [0.83 to 0.94] |
| Therapeutic INR (ischemic stroke only) | Other antithrombotic categories (ischemic stroke only) | Early fatality (30 days) | 1 | Hannon 2015 | NA/3429 | OR | 0.35 [0.23 to 0.52] |
| Therapeutic INR (ischemic stroke only) | Other antithrombotic categories (ischemic stroke only) | Late fatality (1 year) | 1 | Hannon 2015 | NA/1411 | OR | 0.40 [0.25 to 0.64] |
| Warfarin | Placebo | All-cause mortality | 2 | Guimarães 2017 | 253/2900 | OR | 0.74 [0.57 to 0.97] |
| **Antiarrhythmics** |  |  |  |  |  |  |  |
| Class IA drugs | No treatment, placebo, drugs for rate control | All-cause mortality | 2 | Lafuente-Lafuente 2015 | 27/1797 | OR | 2.39 [1.03 to 5.59] |
| Digoxin | Beta-blockers | All-cause mortality | 1 | Trongtorsak 2021 | 24107/85729 | RR | 1.82 [1.65 to 2.01] |
| Digoxin | Calcium channel blockers | All-cause mortality | 1 | Trongtorsak 2021 | 20899/59312 | RR | 1.37 [1.06 to 1.78] |
| Digoxin | No digoxin | All-cause mortality | 8 | Wang 2021 | NA/621478 | HR | 1.17 [1.13 to 1.22] |
| Digoxin | No digoxin | Cardiovascular mortality | 8 | Wang 2021 | NA/63975 | HR | 1.27 [1.08 to 1.50] |
| Digoxin | No digoxin | Sudden cardiac death | 8 | Wang 2021 | NA/48948 | HR | 1.40 [1.23 to 1.60] |
| Digoxin | No digoxin | All-cause mortality | 1 | Wang 2021 | NA | HR | 1.28 [1.11 to 1.47] |
| (AF without HF) | (AF without HF) |  |  |  |  |  |  |
| Dronedarone with digoxin | Placebo with digoxin | Arrhythmic mortality | 6 | Boriani 2019 | 17/3236 | HR | 3.26 [1.06 to 10.00] |
| Dronedarone | Placebo | All-cause mortality | 1 | Boriani 2019 | 20/1070 | HR | 5.47 [1.60 to 18.66] |
| (Digoxin at baseline) | (Digoxin at baseline) |  |  |  |  |  |  |
| Dronedarone | Placebo | Arrhythmic mortality | 1 | Boriani 2019 | 11/1070 | HR | 22.7 [1.33 to 386.17] |
| (Digoxin at baseline) | (Digoxin at baseline) |  |  |  |  |  |  |
| Sotalol | Placebo | All-cause mortality | 4 | Lafuente-Lafuente 2015 | 40/3058 | OR | 2.23 [1.10 to 4.50] |
| Sotalol | Placebo or no treatment | All-cause mortality | 4 | Valembois 2019 | 40/1882 | RR | 2.23 [1.03 to 4.81] |
| Sotalol (PeAF) | Placebo or no treatment (PeAF) | All-cause mortality | 3 | Valembois 2019 | 37/1311 | RR | 2.51 [1.06 to 5.98] |
| **Comorbidities** |  |  |  |  |  |  |  |
| Diabetes | No diabetes | All-cause mortality | 2 | Xu 2022 | NA/518965 | RR | 1.37 [1.23 to 1.53] |
| Diabetes | No diabetes | Cardiovascular mortality | 2 | Xu 2022 | NA/50092 | RR | 1.46 [1.34 to 1.58] |
| Anemia | No Anemia | All-cause mortality | 1 | Tu 2020 | NA/75990 | HR | 1.78 [1.44 to 2.20] |
| Cancer | No cancer | All-cause mortality | 2 | Balomenakis 2023 | 678158/3000762 | OR | 2.17 [1.83 to 2.56] |
| Cancer | No cancer | Non-Cardiovascular mortality | 2 | Balomenakis 2023 | NA/89082 | OR | 6.30 [2.32 to 17.33] |
| COPD | No COPD | All-cause mortality | 2 | Romiti 2021 | 93933/220953 | OR | 2.22 [1.93 to 2.55] |
| COPD | No COPD | Cardiovascular mortality | 2 | Romiti 2021 | 3280/73956 | OR | 1.84 [1.39 to 2.43] |
| Frailty | No frailty | All-cause mortality | 2 | He 2022 | NA/97413 | RR | 2.77 [1.68 to 4.57] |
| LVH | No LVH | All-cause mortality | 1 | Xiang 2021 | NA/13230 | RR | 1.60 [1.42 to 1.79] |
| Peripheral artery disease | No peripheral artery disease | All-cause mortality | 1 | Zhu 2021 | NA/30286 | HR | 1.42 [1.25 to 1.62] |
| **Demographic characteristics** |  |  |  |  |  |  |  |
| High BMI | Low BMI | All-cause mortality | 1 | Liu 2020 | 8977/109478 | RR | 0.86 [0.80 to 0.92] |
| High BMI | Low BMI | Cardiovascular mortality | 1 | Liu 2020 | 2178/63875 | RR | 0.82 [0.71 to 0.95] |
| Latin American | Non-Latin American (Warfarin) | All-cause mortality | 1 | Su 2020 | NA/13058 | RR | 1.45 [1.20 to 1.76] |
| (Warfarin) |  |  |  |  |  |  |  |
| Latin American | Non-Latin American (NOAC) | All-cause mortality | 1 | Su 2020 | NA/26160 | RR | 1.46 [1.27 to 1.68] |
| (NOAC) |  |  |  |  |  |  |  |
| Latin American (NOAC+Warfarin) | Non-Latin American (NOAC+Warfarin) | All-cause mortality | 1 | Su 2020 | NA/39218 | RR | 1.46 [1.30 to 1.63] |
| Overweight (BMI 25-30) | Normal BMI | All-cause mortality | 1 | Zhu 2016 | NA/44441 | RR | 0.78 [0.62 to 0.96] |
| Obesity (BMI ≥ 30) | Normal BMI | All-cause mortality | 2 | Grymonprez 2021 | 3298/55351 | RR | 0.61 [0.52 to 0.71] |
| Smoking | No smoking | All-cause mortality | 1 | Zhu 2016 | NA/17472 | RR | 1.82 [1.33 to 2.49] |
| Smoking | No smoking | Cardiovascular mortality | 1 | Zhu 2016 | NA/4420 | RR | 1.54 [1.31 to 1.81] |
| Underweight (BMI < 18.5) | Normal BMI | All-cause mortality | 2 | Grymonprez 2021 | 1759/34096 | RR | 3.57 [2.50 to 5.11] |
| Elderly group | Younger group | All-cause mortality | 1 | Li 2022 | 433/101062 | RR | 2.61 [1.38 to 4.93] |
| (＞75 years old) | (< 75 years old) |  |  |  |  |  |  |
| **Surgery** |  |  |  |  |  |  |  |
| Ablation | Drug therapy | Cardiovascular mortality | 15 | Zahoor 2023 | NA/19260 | HR | 0.35 [0.25 to 0.50] |
| Ablation | Drug therapy | All-cause mortality | 15 | Zahoor 2023 | NA/5804 | HR | 0.64 [0.45 to 0.93] |
| Catheter Ablation | Non-catheter ablation | All-cause mortality | 1 | Shaik 2022 | NA/270123 | HR | 0.60 [0.51 to 0.71] |
| Catheter Ablation (HF) | Medical therapy (HF) | All-cause mortality | 2 | Asad 2019 | 98/668 | RR | 0.52 [0.35 to 0.76] |
| Catheter Ablation (HF+Non-HF) | Medical therapy (HF+Non-HF) | All-cause mortality | 2 | Asad 2019 | 236/3576 | RR | 0.69 [0.54 to 0.88] |
| Catheter Ablation | AAD | All-cause mortality | 2 | Asad 2019 | 32/619 | RR | 0.46 [0.23 to 0.93] |
| Catheter ablation | Medical therapy (Reduced LVEF) | Mortality | 1 | Ravi 2022 | 113/877 | RR | 0.56 [0.39 to 0.79] |
| (Reduced LVEF) |  |  |  |  |  |  |  |
| LAAC | OAC | All-cause mortality | 4 | Franchin 2023 | 2592/25548 | RR | 0.67 [0.57 to 0.78] |
| LAAC | OAC | Cardiovascular mortality | 4 | Franchin 2023 | 465/5358 | RR | 0.62 [0.51 to 0.74] |
| LAAC during cardiac surgery | No LAAC | All-cause mortality > 2 years | 6 | Prasad 2022 | 10184/307232 | RR | 0.81 [0.68 to 0.97] |
| LAAO | DOAC | Mortality | 8 | Abdelfattah 2023 | NA/94756 | OR | 0.75 [0.55 to 0.99] |
| LAAO | Warfarin | Mortality | 4 | Abdelfattah 2023 | NA/94756 | OR | 0.68 [0.50 to 0.90] |
| **Others (other medications, disease characteristics, treatment strategies)** |  |  |  |  |  |  |  |
| SGLT2 inhibitors | Placebo | Cardiovascular mortality | 2 | Zheng 2024 | 887/9872 | RR | 0.87 [0.76 to 0.98] |
| Statin (low-to-moderate intensity statin) | No statin | All-cause mortality | 1 | Eun 2020 | NA | HR | 0.53 [0.30 to 0.94] |
| Statin (high intensity statin) | No statin | All-cause mortality | 1 | Eun 2020 | NA | HR | 0.47 [0.35 to 0.63] |
| Statin (high intensity statin) | Statin (low-to-moderate intensity statin) | All-cause mortality | 1 | Eun 2020 | NA | HR | 0.70 [0.53 to 0.92] |
| Statin | No statin | All-cause mortality | 2 | Pastori 2021 | 13373/86356 | HR | 0.59 [0.54 to 0.65] |
| Statin | No statin | Cardiovascular mortality | 2 | Pastori 2021 | 470/15102 | HR | 0.75 [0.58 to 0.96] |
| Paroxysmal AF | Persistent AF | All-cause mortality | 1 | Zhang 2019 | 3932/48973 | RR | 0.72 [0.66 to 0.79] |
| Non-paroxysmal AF | Paroxysmal AF | All-cause mortality | 1 | Mentel 2020 | 5897/7834 | OR | 1.90 [1.43 to 2.52] |
| Early rhythm control | Rate control | All-cause mortality | 1 | Han 2023 | 124383/370677 | RR | 0.76 [0.69 to 0.83] |
| Early rhythm control | Rate control | Cardiovascular mortality | 1 | Han 2023 | 25477/320176 | RR | 0.68 [0.63 to 0.74] |
| **Non-significant associations** |  |  |  |  |  |  |  |
| **Anticoagulants or Antiplatelets** |  |  |  |  |  |  |  |
| Adjusted-dose warfarin | Fixed low-dose warfarin | Mortality | 1 | Lip 2006 | 91/1416 | RR | 1.11 [0.81 to 1.52] |
| Apixaban | Dabigatran | Mortality | 4 | Buckley 2022 | 7583/235247 | RR | 1.00 [0.82 to 1.22] |
| Apixaban | Dabigatran 110mg | All-cause mortality | 2 | Mitchell 2013 | NA | HR | 0.98 [0.83 to 1.17] |
| Apixaban | Dabigatran 150mg | All-cause mortality | 2 | Mitchell 2013 | NA | HR | 1.01 [0.86 to 1.20] |
| Apixaban with concomitant aspirin therapy | Warfarin with concomitant aspirin therapy | Vascular mortality | 1 | Bennaghmouch 2018 | NA | HR | 1.05 [0.75 to 1.47] |
| Apixaban–dronedarone combination | Warfarin–dronedarone combination | All-cause mortality | 1 | Boriani 2019 | NA | HR | 1.45 [0.42 to 5.03] |
| Aspirin or Clopidogrel | Warfarin | All-cause mortality | 1 | Melkonian 2017 | 1981/8394 | RR | 0.99 [0.82 to 1.21] |
| Dabigatran 110mg | Warfarin | All-cause mortality | 6 | Escobar 2021 | NA | HR | 0.86 [0.73 to 1.01] |
| Dabigatran 110mg with concomitant aspirin therapy | Warfarin with concomitant aspirin therapy | Vascular mortality | 1 | Bennaghmouch 2018 | NA | HR | 0.86 [0.68 to 1.09] |
| Dabigatran 150mg | Dabigatran 110mg | All-cause mortality | 1 | Mitchell 2013 | NA | HR | 0.97 [0.85 to 1.11] |
| Dabigatran 150mg with concomitant aspirin therapy | Warfarin with concomitant aspirin therapy | Vascular mortality | 1 | Bennaghmouch 2018 | NA | HR | 0.81 [0.63 to 1.03] |
| Dabigatran (unespecified dose but not 75mg ) | Warfarin | All-cause mortality | 1 | Escobar 2021 | NA | HR | 0.92 [0.81 to 1.05] |
| DOAC (BMI 18.5-25) | Warfarin (BMI 18.5-25) | All-cause mortality | 2 | Patel 2024 | 1375/57866 | HR | 0.95 [0.85 to 1.06] |
| DOAC (BMI 30-35) | Warfarin (BMI 30-35) | All-cause mortality | 1 | Patel 2024 | 973/57866 | HR | 0.88 [0.78 to 1.01] |
| DOAC (BMI 35-40) | Warfarin (BMI 35-40) | All-cause mortality | 1 | Patel 2024 | 377/57866 | HR | 1.08 [0.88 to 1.32] |
| DOAC (BMI > 30) | Warfarin (BMI > 30) | All-cause mortality | 1 | Malik 2020 | NA | OR | 0.91 [0.81 to 1.03] |
| DOAC (BMI ≥ 40) | Warfarin (BMI ≥ 40) | All-cause mortality | 1 | Patel 2024 | 201/57866 | HR | 1.33 [1.00 to 1.77] |
| DOAC (Diabetes) | VKA (No Diabetes) | All-cause mortality | 1 | Abdool 2020 | NA | RR | 0.87 [0.75 to 1.02] |
| DOAC (No Diabetes) | VKA (No Diabetes) | Cardiovascular mortality | 1 | Abdool 2020 | NA | RR | 0.93 [0.80 to 1.08] |
| DOAC with fewer concomitant drugs (< 5 concomitant drugs) | Warfarin with fewer concomitant drugs (< 5 concomitant drugs) | Mortality | 1 | Kim 2019 | NA | RR | 0.90 [0.78 to 1.04] |
| DOAC with amiodarone | Warfarin with amiodarone | Mortality | 1 | Kim 2019 | NA | RR | 0.90 [0.72 to 1.11] |
| DOAC with P-glycoprotein inhibitor | Warfarin with P-glycoprotein inhibitor | Mortality | 1 | Kim 2019 | NA | RR | 1.00 [0.84 to 1.19] |
| Edoxaban 30mg (Crcl 30-50) | Warfarin (Crcl 30-50) | Mortality | 1 | Wang 2023 | NA | HR | 0.65 [0.35 to 1.19] |
| Edoxaban 60mg (Crcl 50-95) | Warfarin (Crcl 50-95) | Mortality | 1 | Wang 2023 | NA | HR | 0.46 [0.16 to 1.33] |
| Edoxaban 60mg (Crcl > 95) | Warfarin (Crcl > 95) | Mortality | 1 | Wang 2023 | NA | HR | 1.12 [0.87 to 1.44] |
| Edoxaban 30mg | Edoxaban 60mg | All-cause mortality | 1 | Chen 2015 | 1517/15228 | RR | 0.95 [0.87 to 1.05] |
| Edoxaban 30mg | Aspirin and Clopidogrel | Mortality | 1 | Blann 2015 | NA | RR | 0.84 [0.70 to 1.01] |
| Edoxaban 60mg | Apixaban | All-cause mortality | 1 | Aronow 2020 | NA/94656 | OR | 0.84 [0.70 to 1.01] |
| Edoxaban 60mg | Aspirin | Mortality | 1 | Blann 2015 | NA | RR | 0.86 [0.73 to 1.03] |
| Edoxaban 60mg | Aspirin and Clopidogrel | Mortality | 1 | Blann 2015 | NA | RR | 0.88 [0.74 to 1.05] |
| Edoxaban 60mg | Dabigatran 150mg | All-cause mortality | 1 | Aronow 2020 | NA/94656 | OR | 1.03 [0.87 to 1.22] |
| Edoxaban 60mg | Warfarin | All-cause mortality | 4 | Chen 2015 | 1621/15218 | RR | 0.92 [0.84 to 1.01] |
| Edoxaban 60mg | Rivaroxaban | All-cause mortality | 1 | Aronow 2020 | NA/94656 | OR | 1.10 [0.88 to 1.37] |
| Edoxaban 60mg with concomitant aspirin therapy | Warfarin with concomitant aspirin therapy | Vascular mortality | 1 | Bennaghmouch 2018 | NA | HR | 0.79 [0.62 to 1.01] |
| NOAC (TTR > 65%) | Warfarin (TTR > 65%) | Mortality | 1 | Gómez-Outes 2013 | 1189/17996 | RR | 0.97 [0.87 to 1.09] |
| NOAC (Latin American) | Warfarin (Latin American) | All-cause mortality | 1 | Su 2020 | NA | RR | 0.89 [0.74 to 1.07] |
| NOAC (Previous stroke or TIA) | Warfarin (Previous stroke or TIA) | Mortality | 1 | Gómez-Outes 2013 | 1153/14527 | RR | 0.92 [0.82 to 1.03] |
| OAC | Aspirin | Mortality | 1 | Van Walraven 2002 | 404/4052 | HR | 0.93 [0.76 to 1.13] |
| OAC | Aspirin | Vascular mortality | 1 | Van Walraven 2002 | NA | HR | 0.95 [0.75 to 1.20] |
| OAC | Antiplatelet, placebo, or no antithrombotic treatment | All-cause mortality | 1 | Reddin 2021 | 1319/17905 | OR | 0.89 [0.78 to 1.00] |
| ProPeAFenone | Placebo or no treatment | All-cause mortality | 4 | Valembois 2019 | 2/212 | RR | 0.19 [0.02 to 1.68] |
| Rivaroxaban | Dabigatran | All-cause mortality | 5 | Menichelli 2021 | 4189/317282 | HR | 1.20 [0.99 to 1.50] |
| Rivaroxaban | Warfarin | All-cause mortality | 7 | Waranugraha 2021 | NA/438714 | HR | 0.91 [0.70 to 1.18] |
| Rivaroxaban new-users/ switchers | Warfarin | Mortality | 1 | Bai 2017 | NA | HR | 1.26 [0.80 to 1.72] |
| Rivaroxaban with concomitant aspirin therapy | Warfarin with concomitant aspirin therapy | Vascular mortality | 1 | Bennaghmouch 2018 | 555/11358 | HR | 0.91 [0.74 to 1.13] |
| Warfarin | Antiplatelet therapy | All-cause mortality | 1 | Shi 2015 | 643/10609 | OR | 0.92 [0.78 to 1.08] |
| Warfarin | Aspirin | All-cause mortality | 2 | Lip 2006 | 214/2688 | RR | 0.87 [0.67 to 1.13] |
| Ximelagatran | Control drugs | All-cause mortality | 1 | Liu 2014 | 431/7670 | OR | 0.95 [0.78 to 1.16] |
| **Antiarrhythmics** |  |  |  |  |  |  |  |
| Amiodarone | Class I antiarrhythmics | All-cause mortality | 3 | Lafuente-Lafuente 2015 | 44/643 | OR | 0.59 [0.31 to 1.11] |
| Amiodarone | Placebo | All-cause mortality | 3 | Lafuente-Lafuente 2015 | 17/718 | OR | 1.64 [0.59 to 4.56] |
| Amiodarone | Placebo or no treatment | All-cause mortality | 3 | Valembois 2019 | 17/444 | RR | 1.66 [0.55 to 4.99] |
| Amiodarone (PeAF) | Placebo or no treatment (PeAF) | All-cause mortality | 2 | Valembois 2019 | 17/444 | RR | 1.66 [0.55 to 4.99] |
| Amiodarone | Placebo or rate control drug | All-cause mortality | 1 | Doyle 2009 | 876/21438 | RR | 0.95 [0.81 to 1.11] |
| Amiodarone | Dronedarone | All-cause mortality at 12 months | 1 | Sullivan 2013 | NA | OR | 2.47 [0.47 to 12.87] |
| Amiodarone | Sotalol | All-cause mortality | 4 | Lafuente-Lafuente 2015 | 73/1168 | OR | 0.77 [0.47 to 1.25] |
| Azimilide | Placebo | All-cause mortality | 2 | Lafuente-Lafuente 2015 | 51/3114 | OR | 1.74 [0.99 to 3.06] |
| Azimilide | No treatment, placebo, drugs for rate control | All-cause mortality | 2 | Lafuente-Lafuente 2015 | 51/3114 | OR | 1.74 [0.99 to 3.06] |
| Beta-blockers | No antiarrhythmic treatment | All-cause mortality | 1 | Lafuente-Lafuente 2012 | 4/562 | OR | 2.75 [0.39 to 19.56] |
| Beta-blockers | Calcium channel blockers | All-cause mortality | 1 | Trongtorsak 2021 | 15150/65071 | RR | 0.76 [0.57 to 1.00] |
| Beta-blockers using (COPD) | No beta-blockers using (COPD) | All-cause mortality | 1 | Romiti 2021 | NA | OR | 1.53 [0.68 to 3.43] |
| Beta-blockers using (COPD) | No beta-blockers using (COPD) | Cardiovascular mortality | 1 | Romiti 2021 | NA | OR | 1.83 [0.76 to 4.43] |
| Beta-blockers using (No COPD) | No beta-blockers using (No COPD) | All-cause mortality | 1 | Romiti 2021 | NA | OR | 1.52 [0.64 to 3.60] |
| Beta-blockers using (No COPD) | No beta-blockers using (No COPD) | Cardiovascular mortality | 1 | Romiti 2021 | NA | OR | 1.74 [0.73 to 4.14] |
| Class IA drugs (PeAF) | No treatment, placebo, drugs for rate control (PeAF) | All-cause mortality | 2 | Lafuente-Lafuente 2015 | 25/998 | OR | 2.27 [0.94 to 5.49] |
| Class IB drugs | No treatment, placebo, drugs for rate control | All-cause mortality | 2 | Lafuente-Lafuente 2015 | 12/1321 | OR | 1.89 [0.59 to 6.03] |
| Class IC drugs | No treatment, placebo, drugs for rate control | All-cause mortality | 1 | Lafuente-Lafuente 2015 | 3/1671 | OR | 0.14 [0.01 to 1.88] |
| Class I drugs (PeAF) | No treatment, placebo, drugs for rate control (PeAF) | All-cause mortality | 2 | Lafuente-Lafuente 2015 | 26/1495 | OR | 2.29 [0.96 to 5.48] |
| Class I drugs | No treatment, placebo, drugs for rate control | All-cause mortality | 2 | Lafuente-Lafuente 2015 | 42/4789 | OR | 1.84 [0.95 to 3.57] |
| Class I other drugs (aprindine, bidisomide, pilsicainide) | No treatment, placebo, drugs for rate control | All-cause mortality | 2 | Lafuente-Lafuente 2015 | 13/1383 | OR | 1.94 [0.62 to 6.02] |
| Class II drugs (PeAF) | No treatment, placebo, drugs for rate control (PeAF) | All-cause mortality | 2 | Lafuente-Lafuente 2015 | 4/562 | OR | 2.75 [0.39 to 19.56] |
| Class III drugs | Placebo | All-cause mortality | 3 | Lafuente-Lafuente 2015 | 516/14144 | OR | 1.02 [0.85 to 1.23] |
| Class III drugs | Class I drugs | All-cause mortality | 3 | Lafuente-Lafuente 2015 | 81/2875 | OR | 0.79 [0.49 to 1.26] |
| Class III drugs (PeAF) | No treatment, placebo, drugs for rate control (PeAF) | All-cause mortality | 2 | Lafuente-Lafuente 2015 | 219/3563 | OR | 1.14 [0.83 to 1.57] |
| Digoxin | No digoxin | Non-Cardiovascular mortality | 8 | Wang 2021 | NA/49760 | HR | 1.08 [0.81 to 1.45] |
| Digoxin (AF with HF) | No digoxin (AF with HF) | All-cause mortality | 1 | Wang 2021 | NA/79824 | HR | 1.06 [0.99 to 1.14] |
| Disopyramide | Placebo or no treatment | All-cause mortality | 3 | Lafuente-Lafuente 2015 | 2/146 | OR | 7.56 [0.47 to 122.66] |
| Disopyramide | Other Class I drugs | All-cause mortality | 3 | Lafuente-Lafuente 2015 | 3/113 | OR | 0.46 [0.05 to 4.52] |
| Dronedarone | Placebo | All-cause mortality | 6 | Boriani 2019 | 38/3236 | HR | 1.94 [0.99 to 3.79] |
| Dronedarone | Placebo (No digoxin) | All-cause mortality | 1 | Boriani 2019 | 18/2166 | HR | 0.82 [0.32 to 2.08] |
| Dronedarone | Placebo (No digoxin) | Arrhythmic mortality | 1 | Boriani 2019 | 6/2166 | HR | 0.51 [0.09 to 2.76] |
| Dronedarone | Placebo or active control | All-cause mortality | 1 | Diemberger 2017 | 354/10588 | OR | 1.36 [0.79 to 2.33] |
| Dronedarone | Placebo or active control | Cardiac mortality | 1 | Diemberger 2017 | 229/10588 | OR | 1.51 [0.74 to 3.08] |
| Dronedarone | Placebo or no treatment | All-cause mortality | 4 | Valembois 2019 | 272/6071 | RR | 0.85 [0.67 to 1.07] |
| Dofetilide | Placebo | All-cause mortality | 2 | Lafuente-Lafuente 2015 | 167/1183 | OR | 0.98 [0.68 to 1.41] |
| Dofetilide | Placebo or no treatment | All-cause mortality | 5 | Valembois 2019 | 167/1183 | RR | 0.98 [0.76 to 1.27] |
| Dofetilide (PeAF) | Placebo or no treatment (PeAF) | All-cause mortality | 1 | Valembois 2019 | 167/1183 | RR | 0.98 [0.76 to 1.27] |
| Flecanide | ProPeAFenone | All-cause mortality | 4 | Lafuente-Lafuente 2015 | 1/297 | OR | 0.14 [0.00 to 6.96] |
| Metoprolol | Placebo or no treatment | All-cause mortality | 1 | Valembois 2019 | 4/562 | RR | 2.02 [0.37 to 11.05] |
| Metoprolol (PeAF) | Placebo or no treatment (PeAF) | All-cause mortality | 1 | Valembois 2019 | 4/562 | RR | 2.02 [0.37 to 11.05] |
| Ranolazine | Control AADs (amiodarone, dronedarone, placebo) | Mortality | 1 | Guerra 2016 | 200/7310 | OR | 0.99 [0.78 to 1.25] |
| Sotalol | Class I drugs other than quinidine | All-cause mortality | 3 | Lafuente-Lafuente 2015 | 32/494 | OR | 0.94 [0.44 to 1.99] |
| Sotalol | ProPeAFenone | All-cause mortality at 12 months | 1 | Sullivan 2013 | NA | OR | 5.2 [0.24 to 111.24] |
| Quinidine | Placebo or no treatment | All-cause mortality | 4 | Valembois 2019 | 25/1646 | RR | 2.01 [0.84 to 4.77] |
| Quinidine (PeAF) | Placebo or no treatment (PeAF) | All-cause mortality | 3 | Valembois 2019 | 23/865 | RR | 1.82 [0.73 to 4.53] |
| Quinidine | Other Class I drugs | All-cause mortality | 3 | Lafuente-Lafuente 2015 | 4/526 | OR | 1.04 [0.14 to 7.46] |
| Quinidine (higher dose) | Placebo or no treatment | All-cause mortality | 3 | Valembois 2019 | 12/412 | RR | 2.74 [0.85 to 8.83] |
| Quinidine (lower dose) | Placebo or no treatment | All-cause mortality | 3 | Valembois 2019 | 13/1234 | RR | 1.29 [0.34 to 4.92] |
| Quinidine | Sotalol | All-cause mortality | 3 | Lafuente-Lafuente 2015 | 30/1978 | OR | 0.71 [0.34 to 1.46] |
| **Comorbidities** |  |  |  |  |  |  |  |
| Cancer | No cancer | Cardiovascular mortality | 2 | Balomenakis 2023 | NA/97516 | OR | 0.96 [0.76 to 1.21] |
| **Demographic characteristics** |  |  |  |  |  |  |  |
| Overweight (BMI 25-30) | Normal BMI | Cardiovascular mortality | 1 | Zhu 2016 | NA/12135 | RR | 0.79 [0.58 to 1.08] |
| Men | Women | Cardiovascular mortality | 2 | Marzona 2018 | NA | HR | 0.89 [0.73 to 1.08] |
| Men | Women | All-cause mortality | 2 | Marzona 2018 | NA | HR | 0.86 [0.64 to 1.14] |
| **Surgery** |  |  |  |  |  |  |  |
| Ablation (RAF) | Medical therapy (RAF) | Mortality | 1 | Kheshti 2023 | 6/494 | RR | 0.53 [0.06 to 4.53] |
| Ablation (PeAF) | Medical therapy (PeAF) | Mortality | 1 | Kheshti 2023 | 54/1251 | RR | 0.69 [0.37 to 1.30] |
| Biatrial Maze | Left Atrial Maze | Early death | 1 | Zheng 2016 | 28/1454 | OR | 1.22 [0.57 to 2.60] |
| Biatrial Maze | Left Atrial Maze | Overall death | 1 | Zheng 2016 | 60/1454 | OR | 1.22 [0.70 to 2.14] |
| Biatrial ablation | Isolated left atrial ablation | Mortality within 30 days | 2 | Li 2018 | 30/1080 | OR | 1.02 [0.36 to 2.90] |
| Biatrial ablation | Isolated left atrial ablation | Late mortality | 2 | Li 2018 | 26/932 | OR | 2.31 [0.86 to 6.22] |
| Biventricular pacing mode ungergoing AVNA | Right ventricular pacing mode undergoing AVNA | All-cause mortality | 1 | Chatterjee 2012 | 61/607 | OR | 0.85 [0.40 to 1.82] |
| Biventricular pacing mode ungergoing AVNA | Right ventricular pacing mode undergoing AVNA | Cardiac mortality | 1 | Chatterjee 2012 | 23/454 | OR | 0.59 [0.25 to 1.39] |
| Catheter Ablation | Medical therapy | All-cause mortality | 1 | Asad 2019 | 2/102 | RR | 0.96 [0.10 to 8.95] |
|  | (rate control) |  |  |  |  |  |  |
| Catheter ablation | Medical therapy (Preserved LVEF) | Mortality | 1 | Ravi 2022 | 144/4306 | RR | 0.84 [0.61 to 1.15] |
| (Preserved LVEF) |  |  |  |  |  |  |  |
| Catheter ablation | Medical therapy | Mortality | 1 | Ravi 2022 | 14/1106 | RR | 0.63 [0.22 to 1.80] |
| (no prior AADs use) | (no prior AADs use) |  |  |  |  |  |  |
| Catheter Ablation (Non-HF) | Medical therapy (Non-HF) | All-cause mortality | 2 | Asad 2019 | 13/704 | RR | 0.67 [0.23 to 1.99] |
| LAAC during cardiac surgery | No LAAC | Postoperative mortality | 6 | Prasad 2022 | 6000/281752 | RR | 0.84 [0.56 to 1.28] |
| LAAO | No LAAO | All-cause mortality | 1 | Nso 2022 | 2958/38887 | OR | 0.83 [0.53 to 1.31] |
| Left atrial concomitant surgical ablation in cardiac surgery | Cardiac surgery alone | 30-day mortality | 1 | Wang 2018 | 20/798 | OR | 1.06 [0.43 to 2.60] |
| Left atrial concomitant surgical ablation in cardiac surgery | Cardiac surgery alone | Late mortality | 1 | Wang 2018 | 7/418 | OR | 1.05 [0.30 to 5.29] |
| Pharmacotherapy | AVNA | Mortality | 1 | Chatterjee 2012 | NA/314 | RR | 1.05 [0.29 to 3.85] |
| Surgical ablation in cardiac Surgery | No surgical ablation | All-cause mortality | 2 | McClure 2018 | 125/1869 | RR | 1.07 [0.75 to 1.52] |
| Surgical ablation in cardiac Surgery | No surgical ablation | All-cause ICU mortality during index hospitalization | 2 | McClure 2018 | NA/414 | RR | 2.44 [0.41 to 14.55] |
| Surgical ablation in cardiac Surgery | No surgical ablation | All-cause hospital mortality during index hospitalization | 2 | McClure 2018 | 31/1030 | RR | 1.12 [0.56 to 2.22] |
| **Others (other medications, disease characteristics, treatment strategies)** |  |  |  |  |  |  |  |
| RAAS blockers | No RAAS blockers | All-cause mortality | 1 | Chaugai 2016 | NA/13045 | OR | 0.95 [0.83 to 1.09] |
| RAAS blockers | No RAAS blockers | Cardiovascular mortality | 1 | Chaugai 2016 | NA/11603 | OR | 0.73 [0.51 to 1.05] |
| SGLT2 inhibitors | Placebo | All-cause mortality | 2 | Zheng 2024 | 1525/9872 | RR | 0.96 [0.87 to 1.05] |
| Asymptomatic AF | Symptomatic AF | All-cause mortality | 2 | Sgreccia 2021 | 5816/70326 | OR | 1.03 [0.81 to 1.32] |
| Asymptomatic AF | Symptomatic AF | Cardiovascular mortality | 2 | Sgreccia 2021 | 125/3132 | OR | 0.87 [0.54 to 1.39] |
| Exercise-based cardiac rehabilitation | Usual care | All-cause mortality | 2 | Smart 2018 | 99/883 | RR | 1.08 [0.77 to 1.53] |
| Rate control | Rhythm control | All-cause mortality | 6 | Depoorter 2020 | 13347/28526 | OR | 1.11 [0.78 to 1.59] |
| Rate control | Rhythm control | Cardiovascular mortality | 6 | Depoorter 2020 | 201/2292 | OR | 1.84 [0.95 to 3.57] |

PeAF, Persistent atrial fibrillation; AF, Atrial fibrillation; RAF, Refractory atrial fibrillation; HF, Heart failure; AAD, Anti-arrhythmic Drugs; DOAC, Direct oral anticoagulant; NOAC, Non-vitamin K antagonist oral anticoagulant; OAC, Oral anticoagulant; CrCl, Creatinine clearance rate; BMI, Body mass index; TTR,Time in therapeutic range; TIA, Transient ischemic attack; VKA, Vitamin K antagonists; INR, International normalized ratio; RAAS, Renin-angiotensin-aldosterone system; SGLT2, Sodium-glucose transporter 2; AVNA, Atrioventricular nodal ablation; LVEF, Left ventricular ejection fractions; LAAC, Left atrial appendage closure; LAAO, Left atrial appendage occlusion; COPD, Chronic obstructive pulmonary disease; LA, Left atrial; LVH, Left ventricular hypertrophy; LD, low-dose; HD, high-dose; AMSTAR, a measurement tool to assess systematic reviews; GRADE, Grading of Recommendations Assessment, Development and Evaluation; NA, not available; NS, not significant; HR, hazard ratio; OR, odds ratio; RR, risk ratio; MA, meta analysis; CI, confidence interval.

Table S2. Effects of risk factors on mortality of patients with atrial fibrillation.(continue)

| Risk factor | No. of studies | Effects model | *I*^2^; *Q* test *P* value | Egger test *P* value | AMSTAR | Evidence class | GRADE |
| --- | --- | --- | --- | --- | --- | --- | --- |
|  |  |  |  |  |  |  |  |
| **Significant associations** |  |  |  |  |  |  |  |
| **Anticoagulants or Antiplatelets** |  |  |  |  |  |  |  |
| Adjusted-dose warfarin | 29 | Random | NA | NA | 8 | Class IV | Low |
| Apixaban | 11 | Random | 96%; < 0.00001 | NA | 10 | Class IV | Very low |
| Apixaban | 6 | Random | 97%;< 0.00001 | > 0.05 | 8 | Class IV | Very low |
| Dabigatran 150mg | 11 | Random | 69%; 0.0004 | NA | 7 | Class II | Very low |
| Dabigatran | 9 | Random | 92%; 0.00001 | > 0.05 | 8 | Class III | Very low |
| DOAC (Diabetes) | 3 | Random | NA | NA | 9 | Class IV | Low |
| DOAC (No Diabetes) | 2 | Random | NA | NA | 9 | Class IV | Low |
| DOAC | 3 | Fixed | 38%; 0.20 | NA | 8 | Class II | Low |
| DOAC (BMI 25-30) | 4 | Random | 0%; 0.44 | NA | 9 | Class III | High |
| DOAC with polypharmacy | 4 | Random | 0%; 0.04 | NA | 11 | Class IV | Moderate |
| (≥ 5 concomitant drugs) |  |  |  |  |  |  |  |
| DOAC without amiodarone | 4 | Random | 0%; 0.007 | NA | 11 | Class IV | Moderate |
| DOAC without P-glycoprotein inhibitor | 4 | Random | 0%; 0.01 | NA | 11 | Class IV | Moderate |
| Edoxaban 30mg | 8 | Random | NA | NA | 5 | Class IV | Low |
| Edoxaban 30mg | 8 | Random | NA | NA | 5 | Class IV | Low |
| Edoxaban 30mg | 8 | Random | NA | NA | 5 | Class IV | Low |
| Edoxaban 30mg with concomitant aspirin therapy | 4 | Random | NA | NA | 9 | Class IV | Low |
| Edoxaban 60mg | 8 | Random | NA | NA | 5 | Class IV | Low |
| Edoxaban 60mg | 2 | Random | NA | NA | 8 | Class IV | Low |
| Edoxaban | 3 | Random | 76%; 0.02 | > 0.05 | 8 | Class IV | Very low |
| HD-Rivaroxaban | 3 | Random | NA | NA | 8 | Class IV | Very low |
| LD-Rivaroxaban | 3 | Random | NA | NA | 8 | Class IV | Very low |
| NOAC (TTR < 65%) | 2 | Random | 0%; 0.56 | NA | 8 | Class III | High |
| NOAC with concomitant aspirin therapy | 4 | Random | 0%; 0.635 | NA | 9 | Class III | High |
| NOAC (No previous stroke or TIA) | 3 | Random | 0%; 0.91 | NA | 8 | Class IV | Moderate |
| NOAC (non-Latin American) | 3 | Random | 0%; 0.88 | NA | 9 | Class IV | Moderate |
| Non−Ximelagatran NOAC | 12 | Fixed | 0%; 0.906 | 0.42 | 8 | Class III | High |
| Oral Factor Xa inhibitors | 6 | Random | 0%; 0.90 | NA | 8 | Class III | High |
| Therapeutic INR (ischemic stroke only) | 8 | Random | NA | > 0.3 | 9 | Class IV | Low |
| Therapeutic INR (ischemic stroke only) | 4 | Random | NA | > 0.3 | 9 | Class IV | Low |
| Warfarin | 6 | Random | NA | NA | 3 | Class IV | Low |
| **Antiarrhythmics** |  |  |  |  |  |  |  |
| Class IA drugs | 8 | Fixed | 0%; 0.80 | NA | 10 | Class IV | Low |
| Digoxin | 4 | Random | 26%; 0.26 | NA | 7 | Class II | Low |
| Digoxin | 3 | Random | 77%; 0.01 | NA | 7 | Class IV | Very low |
| Digoxin | 29 | Random | 85.82%; < 0.001 | 0.221 | 9 | Class II | Very low |
| Digoxin | 10 | Random | NA | 0.221 | 9 | Class IV | Very low |
| Digoxin | 5 | Random | NA | 0.221 | 9 | Class II | Very low |
| Digoxin | 10 | Random | 87.85%; < 0.001 | 0.221 | 9 | Class III | Very low |
| (AF without HF) |  |  |  |  |  |  |  |
| Dronedarone with digoxin | 10 | Random | NA | NA | 8 | Class IV | Low |
| Dronedarone | 10 | Random | NA | NA | 8 | Class IV | Low |
| (Digoxin at baseline) |  |  |  |  |  |  |  |
| Dronedarone | 10 | Random | NA | NA | 8 | Class IV | Low |
| (Digoxin at baseline) |  |  |  |  |  |  |  |
| Sotalol | 13 | Fixed | 21%; 0.28 | NA | 10 | Class IV | Low |
| Sotalol | 5 | Fixed | 0%; 0.49 | NA | 11 | Class IV | Moderate |
| Sotalol (PeAF) | 3 | Fixed | 0%; 0.54 | NA | 11 | Class IV | Moderate |
| **Comorbidities** |  |  |  |  |  |  |  |
| Diabetes | 19 | Random | 95.1%; < 0.001 | 0.964 | 8 | Class II | Very low |
| Diabetes | 10 | Fixed | 0%; 0.594 | 0.048 | 8 | Class I | Very low |
| Anemia | 10 | Random | 93%; <0.00001 | 0.02 | 9 | Class II | Very low |
| Cancer | 12 | Random | 98%; < 0.01 | NA | 10 | Class II | Very low |
| Cancer | 6 | Random | 99.7%; < 0.001 | NA | 10 | Class III | Very low |
| COPD | 14 | Random | 89%; < 0.01 | 0.031 | 9 | Class II | Very low |
| COPD | 10 | Random | 84%; < 0.01 | 0.031 | 9 | Class III | Very low |
| Frailty | 8 | Random | 90.9%; < 0.001 | NA | 8 | Class III | Low |
| LVH | 3 | Random | 77.0%; 0.013 | NA | 8 | Class IV | Very low |
| Peripheral artery disease | 5 | Fixed | 49.5%;0.094 | 0.571 | 8 | Class I | Low |
| **Demographic characteristics** |  |  |  |  |  |  |  |
| High BMI | 13 | Random | 85%; <0.00001 | NA | 9 | Class III | Very low |
| High BMI | 7 | Random | 71%; 0.002 | NA | 9 | Class IV | Very low |
| Latin American | 2 | Random | 0%; 0.65 | NA | 9 | Class IV | High |
| (Warfarin) |  |  |  |  |  |  |  |
| Latin American | 4 | Random | 0%; 0.74 | NA | 9 | Class I | High |
| (NOAC) |  |  |  |  |  |  |  |
| Latin American (NOAC+Warfarin) | 6 | Random | 0%; 0.92 | NA | 9 | Class I | High |
| Overweight (BMI 25-30) | 7 | Random | 82%; < 0.00001 | NA | 8 | Class IV | Very low |
| Obesity (BMI ≥ 30) | 5 | Random | 60%; 0.04 | NA | 8 | Class II | Very low |
| Smoking | 6 | Random | 89%; < 0.00001 | NA | 7 | Class IV | Very low |
| Smoking | 4 | Random | 27%; 0.25 | NA | 7 | Class IV | Very low |
| Underweight (BMI < 18.5) | 4 | Random | 81%; 0.001 | NA | 8 | Class II | Low |
| Elderly group | 14 | Random | 65.8%; 0.003 | 0.115 | 9 | Class IV | Very low |
| (＞75 years old) |  |  |  |  |  |  |  |
| **Surgery** |  |  |  |  |  |  |  |
| Ablation | 4 | Random | 0%; 0.72 | < 0.05 | 10 | Class IV | Low |
| Ablation | 6 | Random | 58%; 0.05 | < 0.05 | 10 | Class IV | Very low |
| Catheter Ablation | 8 | Random | 81%; 0.00001 | 0.36 | 8 | Class II | Very low |
| Catheter Ablation (HF) | 4 | Random | 0%; 0.69 | NA | 8 | Class IV | High |
| Catheter Ablation (HF+Non-HF) | 9 | Random | 0%; 0.64 | NA | 8 | Class IV | Moderate |
| Catheter Ablation | 4 | Random | 0%; 0.82 | NA | 8 | Class IV | High |
| Catheter ablation | 5 | Random | 0%; 0.63 | NA | 8 | Class IV | Low |
| (Reduced LVEF) |  |  |  |  |  |  |  |
| LAAC | 8 | Random | 68%; 0.003 | NA | 9 | Class III | Very low |
| LAAC | 5 | Random | 0%; 0.84 | NA | 9 | Class IV | Low |
| LAAC during cardiac surgery | 8 | Random | 79%; < 0.0001 | NA | 8 | Class IV | Very low |
| LAAO | 37 | Random | NA | NA | 9 | Class IV | Very low |
| LAAO | 37 | Random | NA | NA | 9 | Class IV | Low |
| **Others (other medications, disease characteristics, treatment strategies)** |  |  |  |  |  |  |  |
| SGLT2 inhibitors | 5 | Fixed | 0%; 0.58 | NA | 9 | Class IV | Moderate |
| Statin (low-to-moderate intensity statin) | 2 | Random | 31%; 0.23 | NA | 9 | Class IV | Very low |
| Statin (high intensity statin) | 2 | Random | NA | NA | 9 | Class IV | Very low |
| Statin (high intensity statin) | 2 | Random | NA | NA | 9 | Class IV | Very low |
| Statin | 12 | Random | NA | NA | 7 | Class I | Very low |
| Statin | 3 | Random | NA | NA | 7 | Class IV | Very low |
| Paroxysmal AF | 4 | Fixed | 0%; 0.77 | 0.433 | 8 | Class II | High |
| Non-paroxysmal AF | 13 | Random | 63%; < 0.001 | NA | 10 | Class III | Very low |
| Early rhythm control | 6 | Random | 77%; 0.0006 | NA | 9 | Class II | Very low |
| Early rhythm control | 3 | Random | 33%; 0.22 | NA | 9 | Class II | Very low |
| **Non-significant associations** |  |  |  |  |  |  |  |
| **Anticoagulants or Antiplatelets** |  |  |  |  |  |  |  |
| Adjusted-dose warfarin | 4 | Fixed | NA | NA | 9 | NS | Low |
| Apixaban | 9 | Random | 93%; < 0.00001 | NA | 10 | NS | Very low |
| Apixaban | 3 | Random | NA | NA | 8 | NS | Very low |
| Apixaban | 3 | Random | NA | NA | 8 | NS | Very low |
| Apixaban with concomitant aspirin therapy | 4 | Random | NA | NA | 9 | NS | Low |
| Apixaban–dronedarone combination | 10 | Random | NA | NA | 8 | NS | Very low |
| Aspirin or Clopidogrel | 7 | Random | 57%; 0.03 | NA | 10 | NS | Very low |
| Dabigatran 110mg | 7 | Random | 91%; 0.00001 | NA | 7 | NS | Very low |
| Dabigatran 110mg with concomitant aspirin therapy | 4 | Random | NA | NA | 9 | NS | Low |
| Dabigatran 150mg | 3 | Random | NA | NA | 8 | NS | Very low |
| Dabigatran 150mg with concomitant aspirin therapy | 4 | Random | NA | NA | 9 | NS | Low |
| Dabigatran (unespecified dose but not 75mg ) | 4 | Random | 74%; 0.01 | NA | 7 | NS | Very low |
| DOAC (BMI 18.5-25) | 4 | Random | 0%; 0.33 | NA | 9 | NS | Moderate |
| DOAC (BMI 30-35) | 4 | Random | 0%; < 0.06 | NA | 9 | NS | Moderate |
| DOAC (BMI 35-40) | 4 | Random | 0%; < 0.48 | NA | 9 | NS | Moderate |
| DOAC (BMI > 30) | 5 | Random | 9%; 0.36 | NA | 7 | NS | Low |
| DOAC (BMI ≥ 40) | 4 | Random | 0%; < 0.05 | NA | 9 | NS | Moderate |
| DOAC (Diabetes) | 2 | Random | NA | NA | 9 | NS | Low |
| DOAC (No Diabetes) | 3 | Random | NA | NA | 9 | NS | Low |
| DOAC with fewer concomitant drugs (< 5 concomitant drugs) | 4 | Random | 0%; 0.15 | NA | 11 | NS | Moderate |
| DOAC with amiodarone | 4 | Random | 33%; 0.32 | NA | 11 | NS | Moderate |
| DOAC with P-glycoprotein inhibitor | 4 | Random | 0%; 0.99 | NA | 11 | NS | Moderate |
| Edoxaban 30mg (Crcl 30-50) | 2 | Random | 70.2%; 0.067 | NA | 9 | NS | Very low |
| Edoxaban 60mg (Crcl 50-95) | 3 | Random | 74.0%; 0.021 | NA | 9 | NS | Very low |
| Edoxaban 60mg (Crcl > 95) | 2 | Random | 0%; 0.644 | NA | 9 | NS | Very low |
| Edoxaban 30mg | 4 | Fixed | 0%; 0.82 | 0.53 | 10 | NS | Moderate |
| Edoxaban 30mg | 8 | Random | NA | NA | 5 | NS | Low |
| Edoxaban 60mg | 23 | Random | NA | NA | 8 | NS | Low |
| Edoxaban 60mg | 8 | Random | NA | NA | 5 | NS | Low |
| Edoxaban 60mg | 8 | Random | NA | NA | 5 | NS | Low |
| Edoxaban 60mg | 23 | Random | NA | NA | 8 | NS | Low |
| Edoxaban 60mg | 4 | Fixed | 0%; 0.93 | 0.53 | 10 | NS | Moderate |
| Edoxaban 60mg | 23 | Random | NA | NA | 8 | NS | Low |
| Edoxaban 60mg with concomitant aspirin therapy | 4 | Random | NA | NA | 9 | NS | Low |
| NOAC (TTR > 65%) | 2 | Random | 0%; 0.44 | NA | 8 | NS | Moderate |
| NOAC (Latin American) | 3 | Random | 0%; 0.91 | NA | 9 | NS | Moderate |
| NOAC (Previous stroke or TIA) | 3 | Random | 0%; 0.61 | NA | 8 | NS | Moderate |
| OAC | 6 | Random | NA | NA | 3 | NS | Very low |
| OAC | 6 | Random | NA | NA | 3 | NS | Very low |
| OAC | 14 | Random | 2.9%; 0.74 | NA | 9 | NS | Low |
| ProPeAFenone | 2 | Fixed | 0%; 0.66 | NA | 11 | NS | Low |
| Rivaroxaban | 6 | Random | 68.6%; NA | NA | 9 | NS | Very low |
| Rivaroxaban | 7 | Random | 96%; <0.00001 | 0.47 | 8 | NS | Very low |
| Rivaroxaban new-users/ switchers | 2 | Random | NA | NA | 8 | NS | Very low |
| Rivaroxaban with concomitant aspirin therapy | 4 | Random | NA | NA | 9 | NS | Very low |
| Warfarin | 9 | Fixed | 0%; 0.97 | NA | 8 | NS | Moderate |
| Warfarin | 5 | Fixed | NA | NA | 9 | NS | Low |
| Ximelagatran | 4 | Fixed | 0%; 0.680 | 0.42 | 8 | NS | Moderate |
| **Antiarrhythmics** |  |  |  |  |  |  |  |
| Amiodarone | 5 | Fixed | 85%; 0.01 | NA | 10 | NS | Very low |
| Amiodarone | 5 | Fixed | 38%; 0.20 | NA | 10 | NS | Very low |
| Amiodarone | 2 | Fixed | 9.84%; 0.29 | NA | 11 | NS | Low |
| Amiodarone (PeAF) | 2 | Fixed | 9.84%; 0.29 | NA | 11 | NS | Low |
| Amiodarone | 12 | Fixed | 0%; 0.52 | NA | 8 | NS | Very low |
| Amiodarone | 35 | Fixed | NA | NA | 9 | NS | Very low |
| Amiodarone | 6 | Fixed | 59%; 0.09 | NA | 10 | NS | Very low |
| Azimilide | 5 | Fixed | 0%; 0.61 | NA | 10 | NS | Very low |
| Azimilide | 5 | Fixed | 0%; 0.61 | NA | 10 | NS | Very low |
| Beta-blockers | 2 | Random | 66%; 0.08 | NA | 10 | NS | Very low |
| Beta-blockers | 4 | Random | 71%; 0.02 | NA | 7 | NS | Very low |
| Beta-blockers using (COPD) | 9 | Random | 95%; NA | 0.031 | 9 | NS | Very low |
| Beta-blockers using (COPD) | 8 | Random | 93%; NA | 0.031 | 9 | NS | Very low |
| Beta-blockers using (No COPD) | 9 | Random | 99%; NA | 0.031 | 9 | NS | Very low |
| Beta-blockers using (No COPD) | 8 | Random | 98%; NA | 0.031 | 9 | NS | Very low |
| Class IA drugs (PeAF) | 6 | Fixed | 0%; 0.72 | NA | 10 | NS | Very low |
| Class IB drugs | 2 | Fixed | NA | NA | 10 | NS | Very low |
| Class IC drugs | 10 | Fixed | 8%; 0.34 | NA | 10 | NS | Very low |
| Class I drugs (PeAF) | 7 | Fixed | 0%; 0.82 | NA | 10 | NS | Very low |
| Class I drugs | 11 | Fixed | 0%; 0.50 | NA | 10 | NS | Very low |
| Class I other drugs (aprindine, bidisomide, pilsicainide) | 2 | Fixed | 0%; 0.84 | NA | 10 | NS | Very low |
| Class II drugs (PeAF) | 2 | Fixed | 66%; 0.08 | NA | 10 | NS | Very low |
| Class III drugs | 17 | Fixed | 27%; 0.15 | NA | 10 | NS | Very low |
| Class III drugs | 8 | Fixed | 42%; 0.11 | NA | 10 | NS | Very low |
| Class III drugs (PeAF) | 13 | Fixed | 23%; 0.25 | NA | 10 | NS | Very low |
| Digoxin | 6 | Random | NA | 0.221 | 9 | NS | Very low |
| Digoxin (AF with HF) | 14 | Random | 77.03%; < 0.001 | 0.221 | 9 | NS | Very low |
| Disopyramide | 2 | Fixed | NA | NA | 10 | NS | Very low |
| Disopyramide | 2 | Fixed | 60%; 0.12 | NA | 10 | NS | Very low |
| Dronedarone | 10 | Random | NA | NA | 8 | NS | Very low |
| Dronedarone | 10 | Random | NA | NA | 8 | NS | Very low |
| Dronedarone | 10 | Random | NA | NA | 8 | NS | Very low |
| Dronedarone | 11 | Random | 57.0%; 0.03 | NA | 8 | NS | Low |
| Dronedarone | 11 | Random | 64.4%; 0.01 | NA | 8 | NS | Low |
| Dronedarone | 3 | Fixed | 0%; 0.8 | NA | 11 | NS | Low |
| Dofetilide | 3 | Fixed | 0%; 0.78 | NA | 10 | NS | Very low |
| Dofetilide | 3 | Fixed | 0%; 0.92 | NA | 11 | NS | Low |
| Dofetilide (PeAF) | 3 | Fixed | 0%; 0.92 | NA | 11 | NS | Low |
| Flecanide | 2 | Fixed | NA | NA | 10 | NS | Very low |
| Metoprolol | 2 | Fixed | 46.67%; 0.17 | NA | 11 | NS | Very low |
| Metoprolol (PeAF) | 2 | Fixed | 46.67%; 0.17 | NA | 11 | NS | Very low |
| Ranolazine | 5 | Random | NA | NA | 9 | NS | Very low |
| Sotalol | 4 | Fixed | 57%; 0.13 | NA | 10 | NS | Very low |
| Sotalol | 39 | Random | NA | NA | 9 | NS | Very low |
| Quinidine | 6 | Fixed | 0%; 0.94 | NA | 11 | NS | Very low |
| Quinidine (PeAF) | 5 | Fixed | 0%; 0.92 | NA | 11 | NS | Low |
| Quinidine | 2 | Fixed | 75%; 0.04 | NA | 10 | NS | Very low |
| Quinidine (higher dose) | 4 | Fixed | 0%; 0.96 | NA | 11 | NS | Very low |
| Quinidine (lower dose) | 2 | Fixed | 0%; 0.63 | NA | 11 | NS | Low |
| Quinidine | 6 | Fixed | 0%; 0.67 | NA | 10 | NS | Low |
| **Comorbidities** |  |  |  |  |  |  |  |
| Cancer | 6 | Random | 85.3%; < 0.001 | NA | 10 | NS | Very low |
| **Demographic characteristics** |  |  |  |  |  |  |  |
| Overweight (BMI 25-30) | 5 | Random | 57%; 0.05 | NA | 8 | NS | Very low |
| Men | 7 | Random | 0%; NA | NA | 9 | NS | Very low |
| Men | 13 | Random | 91.98%; NA | NA | 9 | NS | Very low |
| **Surgery** |  |  |  |  |  |  |  |
| Ablation (RAF) | 3 | Random | 0%; 0.69 | > 0.05 | 8 | NS | Very low |
| Ablation (PeAF) | 5 | Random | 0%; 0.57 | > 0.05 | 8 | NS | Very low |
| Biatrial Maze | 9 | Random | 0%; 0.541 | 0.112 | 6 | NS | Very low |
| Biatrial Maze | 9 | Random | 25.7%; 0.233 | 0.112 | 6 | NS | Very low |
| Biatrial ablation | 7 | Random | 31%; 0.19 | > 0.05 | 8 | NS | Very low |
| Biatrial ablation | 4 | Random | 0%; 0.76 | > 0.05 | 8 | NS | Very low |
| Biventricular pacing mode ungergoing AVNA | 4 | Random | 31%; 0.23 | NA | 9 | NS | Low |
| Biventricular pacing mode ungergoing AVNA | 3 | Random | 0%; 0.62 | NA | 9 | NS | Low |
| Catheter Ablation | 2 | Random | 0%; 0.32 | NA | 8 | NS | Moderate |
|  |  |  |  |  |  |  |  |
| Catheter ablation | 13 | Random | 0%; 0.90 | NA | 8 | NS | Low |
| (Preserved LVEF) |  |  |  |  |  |  |  |
| Catheter ablation | 6 | Random | 0%; 0.47 | NA | 8 | NS | Low |
| (no prior AADs use) |  |  |  |  |  |  |  |
| Catheter Ablation (Non-HF) | 4 | Random | 0%; 0.81 | NA | 8 | NS | Moderate |
| LAAC during cardiac surgery | 14 | Random | 85%; < 0.00001 | NA | 8 | NS | Very low |
| LAAO | 11 | Random | 91%; < 0.00001 | NA | 8 | NS | Very low |
| Left atrial concomitant surgical ablation in cardiac surgery | 11 | Random | 0%; 0.80 | 0.52 | 8 | NS | Low |
| Left atrial concomitant surgical ablation in cardiac surgery | 7 | Random | 0%; 0.60 | NA | 8 | NS | Low |
| Pharmacotherapy | 5 | Random | NA | NA | 8 | NS | Very low |
| Surgical ablation in cardiac Surgery | 23 | Random | 0%; 0.88 | NA | 9 | NS | Moderate |
| Surgical ablation in cardiac Surgery | 7 | Random | 0%; 0.34 | NA | 9 | NS | Moderate |
| Surgical ablation in cardiac Surgery | 15 | Random | 0%; 0.88 | NA | 9 | NS | Moderate |
| **Others (other medications, disease characteristics, treatment strategies)** |  |  |  |  |  |  |  |
| RAAS blockers | 6 | Random | 11.4%; 0.341 | 0.153 | 8 | NS | Moderate |
| RAAS blockers | 5 | Random | 68.4%; 0.023 | 0.002 | 8 | NS | Very low |
| SGLT2 inhibitors | 5 | Fixed | 39%; 0.16 | NA | 9 | NS | Moderate |
| Asymptomatic AF | 6 | Random | 86%; < 0.00001 | 0.728 | 8 | NS | Very low |
| Asymptomatic AF | 4 | Random | 0%; 0.67 | 0.666 | 8 | NS | Low |
| Exercise-based cardiac rehabilitation | 8 | Fixed | 0%; 0.95 | NA | 8 | NS | Low |
| Rate control | 4 | Random | 79.6%; 0.002 | NA | 9 | NS | Very low |
| Rate control | 2 | Random | 0%; 0.762 | NA | 9 | NS | Very low |

PeAF, Persistent atrial fibrillation; AF, Atrial fibrillation; RAF, Refractory atrial fibrillation; HF, Heart failure; AAD, Anti-arrhythmic Drugs; DOAC, Direct oral anticoagulant; NOAC, Non-vitamin K antagonist oral anticoagulant; OAC, Oral anticoagulant; CrCl, Creatinine clearance rate; BMI, Body mass index; TTR,Time in therapeutic range; TIA, Transient ischemic attack; VKA, Vitamin K antagonists; INR, International normalized ratio; RAAS, Renin-angiotensin-aldosterone system; SGLT2, Sodium-glucose transporter 2; AVNA, Atrioventricular nodal ablation; LVEF, Left ventricular ejection fractions; LAAC, Left atrial appendage closure; LAAO, Left atrial appendage occlusion; COPD, Chronic obstructive pulmonary disease; LA, Left atrial; LVH, Left ventricular hypertrophy; LD, low-dose; HD, high-dose; AMSTAR, a measurement tool to assess systematic reviews; GRADE, Grading of Recommendations Assessment, Development and Evaluation; NA, not available; NS, not significant; HR, hazard ratio; OR, odds ratio; RR, risk ratio; MA, meta analysis; CI, confidence interval.
