# Supplementary material for: Factors associated with mortality in patients with atrial fibrillation: an umbrella review of systematic reviews and meta-analyses
Source: Front Cardiovasc Med. 2026 Oct 13;13:1877770. doi: 10.3389/fcvm.2026.1877770 (PMC13710006; doi:10.3389/fcvm.2026.1877770)
Supplement: Supplementary file 3 [file Table3.docx]

| Table S3. Assessments of AMSTAR scores. | | | | | | | | | | | | | | | | |
| --- | --- | --- | --- | --- | --- | --- | --- | --- | --- | --- | --- | --- | --- | --- | --- | --- |
| Risk factor | Control | Outcomes | Study | A priori design provided | Duplicate study selection & data extraction | At least two electronic databases searched | Status of  publication used as an inclusion criterion | List of  included and excluded studies provided | Characteristics of included  studies provided | Scientific quality of  included studies assessed | Scientific quality of the included studies used  appropriately to form  Conclusions | Appropriate methods to  combine studies | Publication bias assessed | Conflict of interest included | Total AMSTAR Score |  |
| **Anticoagulants or Antiplatelets** | | | | | | | | | | | | | | | |  |
| **DOAC (BMI 18.5-25)** | Warfarin (BMI 18.5-25) | All-cause mortality | Patel 2024 | 1 | 1 | 1 | 0 | 1 | 1 | 1 | 0 | 1 | 1 | 1 | 9 |  |
| **DOAC (BMI 25-30)** | Warfarin (BMI 25-30) | All-cause mortality | Patel 2024 | 1 | 1 | 1 | 0 | 1 | 1 | 1 | 0 | 1 | 1 | 1 | 9 |  |
| **DOAC (BMI 30-35)** | Warfarin (BMI 30-35) | All-cause mortality | Patel 2024 | 1 | 1 | 1 | 0 | 1 | 1 | 1 | 0 | 1 | 1 | 1 | 9 |  |
| **DOAC (BMI 35-40)** | Warfarin (BMI 35-40) | All-cause mortality | Patel 2024 | 1 | 1 | 1 | 0 | 1 | 1 | 1 | 0 | 1 | 1 | 1 | 9 |  |
| **DOAC (BMI > 30)** | Warfarin (BMI > 30) | All-cause mortality | Malik 2020 | 1 | 1 | 1 | 0 | 1 | 1 | 0 | 0 | 1 | 0 | 1 | 7 |  |
| **DOAC (BMI > 40)** | Warfarin (BMI > 40) | All-cause mortality | Patel 2024 | 1 | 1 | 1 | 0 | 1 | 1 | 1 | 0 | 1 | 1 | 1 | 9 |  |
| **NOAC (TTR < 65%)** | Warfarin (TTR < 65%) | Mortality | Gómez-Outes 2013 | 0 | 1 | 1 | 0 | 1 | 1 | 1 | 0 | 1 | 1 | 1 | 8 |  |
| **NOAC (TTR > 65%)** | Warfarin (TTR > 65%) | Mortality | Gómez-Outes 2013 | 0 | 1 | 1 | 0 | 1 | 1 | 1 | 0 | 1 | 1 | 1 | 8 |  |
| **DOAC (Diabetes)** | VKA (Diabetes) | All-cause mortality  Cardiovascular mortality | Abdool 2020 | 1 | 1 | 1 | 0 | 1 | 1 | 1 | 0 | 1 | 1 | 1 | 9 |  |
| **DOAC (No Diabetes)** | VKA (No Diabetes) | All-cause mortality  Cardiovascular mortality | Abdool 2020 | 1 | 1 | 1 | 0 | 1 | 1 | 1 | 0 | 1 | 1 | 1 | 9 |  |
| **DOAC with polypharmacy**  **(≥ 5 concomitant drugs)** | Warfarin with polypharmacy  (≥ 5 concomitant drugs) | Mortality | Kim 2019 | 1 | 1 | 1 | 1 | 1 | 1 | 1 | 1 | 1 | 1 | 1 | 11 |  |
| **DOAC with fewer concomitant drugs (< 5 concomitant drugs)** | Warfarin with fewer concomitant drugs (< 5 concomitant drugs) | Mortality | Kim 2019 | 1 | 1 | 1 | 1 | 1 | 1 | 1 | 1 | 1 | 1 | 1 | 11 |  |
| **DOAC with amiodarone** | Warfarin with amiodarone | Mortality | Kim 2019 | 1 | 1 | 1 | 1 | 1 | 1 | 1 | 1 | 1 | 1 | 1 | 11 |  |
| **DOAC without amiodarone** | Warfarin without amiodarone | Mortality | Kim 2019 | 1 | 1 | 1 | 1 | 1 | 1 | 1 | 1 | 1 | 1 | 1 | 11 |  |
| **DOAC with P-glycoprotein inhibitor** | Warfarin with P-glycoprotein inhibitor | Mortality | Kim 2019 | 1 | 1 | 1 | 1 | 1 | 1 | 1 | 1 | 1 | 1 | 1 | 11 |  |
| **DOAC without P-glycoprotein inhibitor** | Warfarin without P-glycoprotein inhibitor | Mortality | Kim 2019 | 1 | 1 | 1 | 1 | 1 | 1 | 1 | 1 | 1 | 1 | 1 | 11 |  |
| **NOAC (Previous stroke or TIA)** | Warfarin (Previous stroke or TIA) | Mortality | Gómez-Outes 2013 | 0 | 1 | 1 | 0 | 1 | 1 | 1 | 0 | 1 | 1 | 1 | 8 |  |
| **NOAC (No previous stroke or TIA)** | Warfarin (No previous stroke or TIA) | Mortality | Gómez-Outes 2013 | 0 | 1 | 1 | 0 | 1 | 1 | 1 | 0 | 1 | 1 | 1 | 8 |  |
| **DOAC** | Warfarin | Mortality | Xu 2023 | 0 | 1 | 1 | 0 | 1 | 1 | 1 | 0 | 1 | 1 | 1 | 8 |  |
| **OAC** | Aspirin | Mortality  Vascular mortality | Van Walraven 2002 | 0 | 0 | 0 | 0 | 0 | 1 | 0 | 0 | 1 | 0 | 1 | 3 |  |
| **OAC** | Antiplatelet, placebo, or no antithrombotic treatment | All-cause mortality | Reddin 2021 | 1 | 1 | 1 | 0 | 1 | 1 | 1 | 0 | 1 | 1 | 1 | 9 |  |
| **Oral Factor Xa inhibitors** | VKA | All-cause mortality | Garg 2016 | 0 | 1 | 1 | 0 | 1 | 1 | 1 | 0 | 1 | 1 | 1 | 8 |  |
| **Aspirin or Clopidogrel** | Warfarin | All-cause mortality | Melkonian 2017 | 1 | 1 | 1 | 1 | 1 | 1 | 1 | 0 | 1 | 1 | 1 | 10 |  |
| **Apixaban** | Dabigatran 110mg | All-cause mortality | Mitchell 2013 | 0 | 1 | 1 | 1 | 0 | 1 | 1 | 0 | 1 | 1 | 1 | 8 |  |
| **Apixaban** | Dabigatran 150mg | All-cause mortality | Mitchell 2013 | 0 | 1 | 1 | 1 | 0 | 1 | 1 | 0 | 1 | 1 | 1 | 8 |  |
| **Apixaban** | Dabigatran | Mortality | Buckley 2022 | 1 | 1 | 1 | 1 | 1 | 1 | 1 | 0 | 1 | 1 | 1 | 10 |  |
| **Apixaban** | Warfarin | All-cause mortality | Waranugraha 2021 | 0 | 1 | 1 | 0 | 1 | 1 | 1 | 0 | 1 | 1 | 1 | 8 |  |
| **Apixaban** | Rivaroxaban | Mortality | Buckley 2022 | 1 | 1 | 1 | 1 | 1 | 1 | 1 | 0 | 1 | 1 | 1 | 10 |  |
| **Apixaban with concomitant aspirin therapy** | Warfarin with concomitant aspirin therapy | Vascular mortality | Bennaghmouch 2018 | 0 | 1 | 1 | 1 | 1 | 1 | 1 | 0 | 1 | 1 | 1 | 9 |  |
| **Adjusted-dose warfarin** | Other antithrombotic treatment or placebo | All-cause mortality | Hart 2007 | 0 | 1 | 1 | 1 | 0 | 1 | 1 | 0 | 1 | 1 | 1 | 8 |  |
| **Dabigatran 150mg** | Dabigatran 110mg | All-cause mortality | Mitchell 2013 | 0 | 1 | 1 | 1 | 0 | 1 | 1 | 0 | 1 | 1 | 1 | 8 |  |
| **Dabigatran 110mg** | Warfarin | All-cause mortality | Escobar 2021 | 1 | 1 | 1 | 0 | 1 | 0 | 0 | 0 | 1 | 1 | 1 | 7 |  |
| **Dabigatran 110mg with concomitant aspirin therapy** | Warfarin with concomitant aspirin therapy | Vascular mortality | Bennaghmouch 2018 | 0 | 1 | 1 | 1 | 1 | 1 | 1 | 0 | 1 | 1 | 1 | 9 |  |
| **Dabigatran 150mg** | Warfarin | All-cause mortality | Escobar 2021 | 1 | 1 | 1 | 0 | 1 | 0 | 0 | 0 | 1 | 1 | 1 | 7 |  |
| **Dabigatran 150mg with concomitant aspirin therapy** | Warfarin with concomitant aspirin therapy | Vascular mortality | Bennaghmouch 2018 | 0 | 1 | 1 | 1 | 1 | 1 | 1 | 0 | 1 | 1 | 1 | 9 |  |
| **Dabigatran** | Warfarin | All-cause mortality | Waranugraha 2021 | 0 | 1 | 1 | 0 | 1 | 1 | 1 | 0 | 1 | 1 | 1 | 8 |  |
| **Dabigatran (unespecified dose but not 75mg )** | Warfarin | All-cause mortality | Escobar 2021 | 1 | 1 | 1 | 0 | 1 | 0 | 0 | 0 | 1 | 1 | 1 | 7 |  |
| **Edoxaban 30mg** | Aspirin | Mortality | Blann 2015 | 0 | 1 | 1 | 0 | 0 | 0 | 1 | 0 | 1 | 0 | 1 | 5 |  |
| **Edoxaban 30mg** | Aspirin and Clopidogrel | Mortality | Blann 2015 | 0 | 1 | 1 | 0 | 0 | 0 | 1 | 0 | 1 | 0 | 1 | 5 |  |
| **Edoxaban 30mg** | Edoxaban 60mg | All-cause mortality | Chen 2015 | 0 | 1 | 1 | 1 | 1 | 1 | 1 | 0 | 1 | 1 | 1 | 10 |  |
| **Edoxaban 30mg** | Placebo | Mortality | Blann 2015 | 0 | 1 | 1 | 0 | 0 | 0 | 1 | 0 | 1 | 0 | 1 | 5 |  |
| **Edoxaban 30mg** | Warfarin | Mortality | Blann 2015 | 0 | 1 | 1 | 0 | 0 | 0 | 1 | 0 | 1 | 0 | 1 | 5 |  |
| **Edoxaban 30mg with concomitant aspirin therapy** | Warfarin with concomitant aspirin therapy | Vascular mortality | Bennaghmouch 2018 | 0 | 1 | 1 | 1 | 1 | 1 | 1 | 0 | 1 | 1 | 1 | 9 |  |
| **Edoxaban 60mg** | Apixaban | All-cause mortality | Aronow 2020 | 0 | 1 | 1 | 1 | 0 | 0 | 1 | 1 | 1 | 1 | 1 | 8 |  |
| **Edoxaban 60mg** | Aspirin | Mortality | Blann 2015 | 0 | 1 | 1 | 0 | 0 | 0 | 1 | 0 | 1 | 0 | 1 | 5 |  |
| **Edoxaban 60mg** | Aspirin and Clopidogrel | Mortality | Blann 2015 | 0 | 1 | 1 | 0 | 0 | 0 | 1 | 0 | 1 | 0 | 1 | 5 |  |
| **Edoxaban 60mg** | Placebo | Mortality | Blann 2015 | 0 | 1 | 1 | 0 | 0 | 0 | 1 | 0 | 1 | 0 | 1 | 5 |  |
| **Edoxaban 60mg** | Dabigatran 150mg | All-cause mortality | Aronow 2020 | 0 | 1 | 1 | 1 | 0 | 0 | 1 | 1 | 1 | 1 | 1 | 8 |  |
| **Edoxaban 60mg** | Warfarin | All-cause mortality  Cardiovascular mortality  Noncardiovascular and nonmalignancy mortality | Aronow 2020 | 0 | 1 | 1 | 1 | 0 | 0 | 1 | 1 | 1 | 1 | 1 | 8 |  |
| **Edoxaban 60mg** | Rivaroxaban | All-cause mortality | Aronow 2020 | 0 | 1 | 1 | 1 | 0 | 0 | 1 | 1 | 1 | 1 | 1 | 8 |  |
| **Edoxaban 60mg** | Warfarin | All-cause mortality | Chen 2015 | 0 | 1 | 1 | 1 | 1 | 1 | 1 | 0 | 1 | 1 | 1 | 10 |  |
| **Edoxaban 60mg with concomitant aspirin therapy** | Warfarin with concomitant aspirin therapy | Vascular mortality | Bennaghmouch 2018 | 0 | 1 | 1 | 1 | 1 | 1 | 1 | 0 | 1 | 1 | 1 | 9 |  |
| **Edoxaban** | Warfarin | All-cause mortality | Waranugraha 2021 | 0 | 1 | 1 | 0 | 1 | 1 | 1 | 0 | 1 | 1 | 1 | 8 |  |
| **Edoxaban 30mg (Crcl 30-50)** | Warfarin (Crcl 30-50) | Mortality | Wang 2023 | 1 | 1 | 1 | 0 | 1 | 1 | 1 | 0 | 1 | 1 | 1 | 9 |  |
| **Edoxaban 60mg (Crcl 50-95)** | Warfarin (Crcl 50-95) | Mortality | Wang 2023 | 1 | 1 | 1 | 0 | 1 | 1 | 1 | 0 | 1 | 1 | 1 | 9 |  |
| **Edoxaban 60mg (Crcl > 95)** | Warfarin (Crcl > 95) | Mortality | Wang 2023 | 1 | 1 | 1 | 0 | 1 | 1 | 1 | 0 | 1 | 1 | 1 | 9 |  |
| **Edoxaban 60mg (Latin American)** | Warfarin (Latin American) | All-cause mortality | Su 2020 | 1 | 1 | 1 | 0 | 1 | 1 | 1 | 0 | 1 | 1 | 1 | 9 |  |
| **NOAC (Latin American)** | Warfarin (Latin American) | All-cause mortality | Su 2020 | 1 | 1 | 1 | 0 | 1 | 1 | 1 | 0 | 1 | 1 | 1 | 9 |  |
| **Rivaroxaban** | Dabigatran | All-cause mortality | Menichelli 2021 | 1 | 1 | 1 | 0 | 1 | 1 | 1 | 0 | 1 | 1 | 1 | 9 |  |
| **LD-Rivaroxaban** | LD-Dabigatran | Mortality | Bai 2017 | 0 | 1 | 1 | 0 | 1 | 1 | 1 | 0 | 1 | 1 | 1 | 8 |  |
| **HD-Rivaroxaban** | HD-Dabigatran | Mortality | Bai 2017 | 0 | 1 | 1 | 0 | 1 | 1 | 1 | 0 | 1 | 1 | 1 | 8 |  |
| **Rivaroxaban** | Warfarin | All-cause mortality | Waranugraha 2021 | 0 | 1 | 1 | 0 | 1 | 1 | 1 | 0 | 1 | 1 | 1 | 8 |  |
| **Rivaroxaban new-users/switchers** | Warfarin | Mortality | Bai 2017 | 0 | 1 | 1 | 0 | 1 | 1 | 1 | 0 | 1 | 1 | 1 | 8 |  |
| **Rivaroxaban with concomitant aspirin therapy** | Warfarin with concomitant aspirin therapy | Vascular mortality | Bennaghmouch 2018 | 0 | 1 | 1 | 1 | 1 | 1 | 1 | 0 | 1 | 1 | 1 | 9 |  |
| **NOAC with concomitant aspirin therapy** | Warfarin with concomitant aspirin therapy | Vascular mortality | Bennaghmouch 2018 | 0 | 1 | 1 | 1 | 1 | 1 | 1 | 0 | 1 | 1 | 1 | 9 |  |
| **Non−Ximelagatran NOAC** | Control drugs | All-cause mortality | Liu 2014 | 0 | 1 | 1 | 0 | 1 | 1 | 1 | 0 | 1 | 1 | 1 | 8 |  |
| **Warfarin** | Placebo | All-cause mortality | Guimarães 2017 | 0 | 0 | 0 | 0 | 0 | 1 | 0 | 0 | 1 | 0 | 1 | 3 |  |
| **Warfarin** | Antiplatelet therapy | All-cause mortality | Shi 2015 | 0 | 1 | 1 | 0 | 0 | 1 | 1 | 1 | 1 | 1 | 1 | 8 |  |
| **Warfarin** | Aspirin | All-cause mortality | Lip 2006 |  |  |  |  |  |  |  |  |  |  |  |  |  |
| **Adjusted-dose warfarin** | Fixed low-dose warfarin | Mortality | Lip 2006 | 0 | 1 | 1 | 1 | 1 | 1 | 1 | 0 | 1 | 1 | 1 | 9 |  |
| **Therapeutic INR (ischemic stroke only)** | Other antithrombotic categories (ischemic stroke only) | Early fatality (30 days)  Late fatality (1 year) | Hannon 2015 | 0 | 1 | 1 | 1 | 1 | 1 | 1 | 0 | 1 | 1 | 1 | 9 |  |
| **Ximelagatran** | Control drugs | All-cause mortality | Liu 2014 | 0 | 1 | 1 | 0 | 1 | 1 | 1 | 0 | 1 | 1 | 1 | 8 |  |
| **Antiarrhythmics** | | | | | | | | | | | | | | | |  |
| **Azimilide** | Placebo | All-cause mortality | Lafuente-Lafuente 2015 | 1 | 1 | 1 | 1 | 1 | 1 | 1 | 0 | 1 | 1 | 1 | 10 |  |
| **Azimilide** | No treatment, placebo, drugs for rate control | All-cause mortality | Lafuente-Lafuente 2015 | 1 | 1 | 1 | 1 | 1 | 1 | 1 | 0 | 1 | 1 | 1 | 10 |  |
| **Amiodarone** | Class I antiarrhythmics | All-cause mortality | Lafuente-Lafuente 2015 | 1 | 1 | 1 | 1 | 1 | 1 | 1 | 0 | 1 | 1 | 1 | 10 |  |
| **Amiodarone** | Placebo | All-cause mortality | Lafuente-Lafuente 2015 | 1 | 1 | 1 | 1 | 1 | 1 | 1 | 0 | 1 | 1 | 1 | 10 |  |
| **Amiodarone** | Placebo or no treatment | All-cause mortality | Valembois 2019 | 1 | 1 | 1 | 1 | 1 | 1 | 1 | 1 | 1 | 1 | 1 | 11 |  |
| **Amiodarone (PeAF)** | Placebo or no treatment (PeAF) | All-cause mortality | Valembois 2019 | 1 | 1 | 1 | 1 | 1 | 1 | 1 | 1 | 1 | 1 | 1 | 11 |  |
| **Amiodarone** | Placebo or rate control drug | All-cause mortality | Doyle 2009 | 0 | 1 | 1 | 0 | 1 | 1 | 1 | 0 | 1 | 1 | 1 | 8 |  |
| **Amiodarone** | Dronedarone | All-cause mortality at 12 months | Sullivan 2013 | 0 | 1 | 1 | 1 | 1 | 0 | 1 | 0 | 1 | 1 | 1 | 9 |  |
| **Amiodarone** | Sotalol | All-cause mortality | Lafuente-Lafuente 2015 | 1 | 1 | 1 | 1 | 1 | 1 | 1 | 0 | 1 | 1 | 1 | 10 |  |
| **Beta-blockers** | No antiarrhythmic treatment | All-cause mortality | Lafuente-Lafuente 2012 | 1 | 1 | 1 | 1 | 1 | 1 | 1 | 0 | 1 | 1 | 1 | 10 |  |
| **Beta-blockers** | Calcium channel blockers | All-cause mortality | Trongtorsak 2021 | 0 | 1 | 1 | 0 | 1 | 1 | 0 | 0 | 1 | 1 | 1 | 7 |  |
| **Beta-blockers using (COPD)** | No beta-blockers using (COPD) | All-cause mortality  Cardiovascular mortality | Romiti 2021 | 1 | 1 | 1 | 0 | 1 | 1 | 1 | 0 | 1 | 1 | 1 | 9 |  |
| **Beta-blockers using (No COPD)** | No beta-blockers using (No COPD) | All-cause mortality  Cardiovascular mortality | Romiti 2021 | 1 | 1 | 1 | 0 | 1 | 1 | 1 | 0 | 1 | 1 | 1 | 9 |  |
| **Class I drugs** | No treatment, placebo, drugs for rate control | All-cause mortality | Lafuente-Lafuente 2015 | 1 | 1 | 1 | 1 | 1 | 1 | 1 | 0 | 1 | 1 | 1 | 10 |  |
| **Class I other drugs (aprindine, bidisomide, pilsicainide)** | No treatment, placebo, drugs for rate control | All-cause mortality | Lafuente-Lafuente 2015 | 1 | 1 | 1 | 1 | 1 | 1 | 1 | 0 | 1 | 1 | 1 | 10 |  |
| **Class I drugs (PeAF)** | No treatment, placebo, drugs for rate control (PeAF) | All-cause mortality | Lafuente-Lafuente 2015 | 1 | 1 | 1 | 1 | 1 | 1 | 1 | 0 | 1 | 1 | 1 | 10 |  |
| **Class IA drugs** | No treatment, placebo, drugs for rate control | All-cause mortality | Lafuente-Lafuente 2015 | 1 | 1 | 1 | 1 | 1 | 1 | 1 | 0 | 1 | 1 | 1 | 10 |  |
| **Class IA drugs (PeAF)** | No treatment, placebo, drugs for rate control (PeAF) | All-cause mortality | Lafuente-Lafuente 2015 | 1 | 1 | 1 | 1 | 1 | 1 | 1 | 0 | 1 | 1 | 1 | 10 |  |
| **Class IB drugs** | No treatment, placebo, drugs for rate control | All-cause mortality | Lafuente-Lafuente 2015 | 1 | 1 | 1 | 1 | 1 | 1 | 1 | 0 | 1 | 1 | 1 | 10 |  |
| **Class IC drugs** | No treatment, placebo, drugs for rate control | All-cause mortality | Lafuente-Lafuente 2015 | 1 | 1 | 1 | 1 | 1 | 1 | 1 | 0 | 1 | 1 | 1 | 10 |  |
| **Class II drugs (PeAF)** | No treatment, placebo, drugs for rate control (PeAF) | All-cause mortality | Lafuente-Lafuente 2015 | 1 | 1 | 1 | 1 | 1 | 1 | 1 | 0 | 1 | 1 | 1 | 10 |  |
| **Class III drugs** | Placebo | All-cause mortality | Lafuente-Lafuente 2015 | 1 | 1 | 1 | 1 | 1 | 1 | 1 | 0 | 1 | 1 | 1 | 10 |  |
| **Class III drugs** | Class I drugs | All-cause mortality | Lafuente-Lafuente 2015 | 1 | 1 | 1 | 1 | 1 | 1 | 1 | 0 | 1 | 1 | 1 | 10 |  |
| **Class III drugs (PeAF)** | No treatment, placebo, drugs for rate control (PeAF) | All-cause mortality | Lafuente-Lafuente 2015 | 1 | 1 | 1 | 1 | 1 | 1 | 1 | 0 | 1 | 1 | 1 | 10 |  |
| **Disopyramide** | Other Class I drugs | All-cause mortality | Lafuente-Lafuente 2015 | 1 | 1 | 1 | 1 | 1 | 1 | 1 | 0 | 1 | 1 | 1 | 10 |  |
| **Disopyramide** | Placebo or no treatment | All-cause mortality | Lafuente-Lafuente 2015 | 1 | 1 | 1 | 1 | 1 | 1 | 1 | 0 | 1 | 1 | 1 | 10 |  |
| **Digoxin** | Beta-blockers | All-cause mortality | Trongtorsak 2021 | 0 | 1 | 1 | 0 | 1 | 1 | 0 | 0 | 1 | 1 | 1 | 7 |  |
| **Digoxin** | Calcium channel blockers | All-cause mortality | Trongtorsak 2021 | 0 | 1 | 1 | 0 | 1 | 1 | 0 | 0 | 1 | 1 | 1 | 7 |  |
| **Digoxin** | No digoxin | All-cause mortality  Cardiovascular mortality  Non-Cardiovascular mortality  Sudden cardiac death | Wang 2021 | 1 | 1 | 1 | 0 | 1 | 1 | 1 | 0 | 1 | 1 | 1 | 9 |  |
| **Digoxin (AF without HF)** | No digoxin (AF without HF) | All-cause mortality | Wang 2021 | 1 | 1 | 1 | 0 | 1 | 1 | 1 | 0 | 1 | 1 | 1 | 9 |  |
| **Digoxin (AF with HF)** | No digoxin (AF with HF) | All-cause mortality | Wang 2021 | 1 | 1 | 1 | 0 | 1 | 1 | 1 | 0 | 1 | 1 | 1 | 9 |  |
| **Dofetilide** | Placebo | All-cause mortality | Lafuente-Lafuente 2015 | 1 | 1 | 1 | 1 | 1 | 1 | 1 | 0 | 1 | 1 | 1 | 10 |  |
| **Dofetilide** | Placebo or no treatment | All-cause mortality | Valembois 2019 | 1 | 1 | 1 | 1 | 1 | 1 | 1 | 1 | 1 | 1 | 1 | 11 |  |
| **Dofetilide (PeAF)** | Placebo or no treatment (PeAF) | All-cause mortality | Valembois 2019 | 1 | 1 | 1 | 1 | 1 | 1 | 1 | 1 | 1 | 1 | 1 | 11 |  |
| **Dronedarone with digoxin** | Placebo with digoxin | All-cause mortality  Arrhythmic mortality | Boriani 2019 | 0 | 1 | 1 | 1 | 0 | 1 | 1 | 0 | 1 | 1 | 1 | 8 |  |
| **Dronedarone**  **(No digoxin)** | Placebo  (No digoxin) | All-cause mortality  Arrhythmic mortality | Boriani 2019 | 0 | 1 | 1 | 1 | 0 | 1 | 1 | 0 | 1 | 1 | 1 | 8 |  |
| **Dronedarone**  **(Digoxin at baseline)** | Placebo  (Digoxin at baseline) | All-cause mortality  Arrhythmic mortality | Boriani 2019 | 0 | 1 | 1 | 1 | 0 | 1 | 1 | 0 | 1 | 1 | 1 | 8 |  |
| **Dronedarone** | Placebo or active control | Overall mortality  Cardiac mortality | Diemberger 2017 | 0 | 1 | 1 | 1 | 0 | 1 | 1 | 0 | 1 | 1 | 1 | 8 |  |
| **Dronedarone** | Placebo or no treatment | All-cause mortality | Valembois 2019 | 1 | 1 | 1 | 1 | 1 | 1 | 1 | 1 | 1 | 1 | 1 | 11 |  |
| **Apixaban–dronedarone combination** | Warfarin–dronedarone combination | All-cause mortality | Boriani 2019 | 0 | 1 | 1 | 1 | 0 | 1 | 1 | 0 | 1 | 1 | 1 | 8 |  |
| **Flecanide** | ProPeAFenone | All-cause mortality | Lafuente-Lafuente 2015 | 1 | 1 | 1 | 1 | 1 | 1 | 1 | 0 | 1 | 1 | 1 | 10 |  |
| **Metoprolol** | Placebo or no treatment | All-cause mortality | Valembois 2019 | 1 | 1 | 1 | 1 | 1 | 1 | 1 | 1 | 1 | 1 | 1 | 11 |  |
| **Metoprolol (PeAF)** | Placebo or no treatment (PeAF) | All-cause mortality | Valembois 2019 | 1 | 1 | 1 | 1 | 1 | 1 | 1 | 1 | 1 | 1 | 1 | 11 |  |
| **ProPeAFenone** | Placebo or no treatment | All-cause mortality | Valembois 2019 | 1 | 1 | 1 | 1 | 1 | 1 | 1 | 1 | 1 | 1 | 1 | 11 |  |
| **Quinidine** | Placebo or no treatment | All-cause mortality | Valembois 2019 | 1 | 1 | 1 | 1 | 1 | 1 | 1 | 1 | 1 | 1 | 1 | 11 |  |
| **Quinidine (PeAF)** | Placebo or no treatment (PeAF) | All-cause mortality | Valembois 2019 | 1 | 1 | 1 | 1 | 1 | 1 | 1 | 1 | 1 | 1 | 1 | 11 |  |
| **Quinidine** | Other Class I drugs | All-cause mortality | Lafuente-Lafuente 2015 | 1 | 1 | 1 | 1 | 1 | 1 | 1 | 0 | 1 | 1 | 1 | 10 |  |
| **Quinidine (higher dose)** | Placebo or no treatment | All-cause mortality | Valembois 2019 | 1 | 1 | 1 | 1 | 1 | 1 | 1 | 1 | 1 | 1 | 1 | 11 |  |
| **Quinidine (lower dose)** | Placebo or no treatment | All-cause mortality | Valembois 2019 | 1 | 1 | 1 | 1 | 1 | 1 | 1 | 1 | 1 | 1 | 1 | 11 |  |
| **Ranolazine** | Control AADs (amiodarone, dronedarone, placebo) | Mortality | Guerra 2016 | 1 | 1 | 1 | 0 | 1 | 1 | 1 | 0 | 1 | 1 | 1 | 9 |  |
| **Sotalol** | Placebo | All-cause mortality | Lafuente-Lafuente 2015 | 1 | 1 | 1 | 1 | 1 | 1 | 1 | 0 | 1 | 1 | 1 | 10 |  |
| **Sotalol** | Placebo or no treatment | All-cause mortality | Valembois 2019 | 1 | 1 | 1 | 1 | 1 | 1 | 1 | 1 | 1 | 1 | 1 | 11 |  |
| **Sotalol (PeAF)** | Placebo or no treatment (PeAF) | All-cause mortality | Valembois 2019 | 1 | 1 | 1 | 1 | 1 | 1 | 1 | 1 | 1 | 1 | 1 | 11 |  |
| **Sotalol** | ProPeAFenone | All-cause mortality at 12 months | Sullivan 2013 | 0 | 1 | 1 | 1 | 1 | 0 | 1 | 0 | 1 | 1 | 1 | 9 |  |
| **Quinidine** | Sotalol | All-cause mortality | Lafuente-Lafuente 2015 | 1 | 1 | 1 | 1 | 1 | 1 | 1 | 0 | 1 | 1 | 1 | 10 |  |
| **Sotalol** | Class I drugs other than quinidine | All-cause mortality | Lafuente-Lafuente 2015 | 1 | 1 | 1 | 1 | 1 | 1 | 1 | 0 | 1 | 1 | 1 | 10 |  |
| **Comorbidities** | | | | | | | | | | | | | | | |  |
| **Anemia** | No Anemia | All-cause mortality | Tu 2020 | 1 | 1 | 1 | 0 | 1 | 1 | 1 | 0 | 1 | 1 | 1 | 9 |  |
| **Cancer** | No cancer | All-cause mortality  Cardiovascular mortality  Non-Cardiovascular mortality | Balomenakis 2023 | 1 | 1 | 1 | 0 | 1 | 1 | 1 | 1 | 1 | 1 | 1 | 10 |  |
| **COPD** | No COPD | All-cause mortality  Cardiovascular mortality | Romiti 2021 | 1 | 1 | 1 | 0 | 1 | 1 | 1 | 0 | 1 | 1 | 1 | 9 |  |
| **Diabetes** | No diabetes | All-cause mortality  Cardiovascular mortality | Xu 2022 | 0 | 1 | 1 | 0 | 1 | 1 | 1 | 0 | 1 | 1 | 1 | 8 |  |
| **Frailty** | No frailty | All-cause mortality | He 2022 | 0 | 1 | 1 | 0 | 1 | 1 | 1 | 0 | 1 | 1 | 1 | 8 |  |
| **LVH** | No LVH | All-cause mortality | Xiang 2021 | 0 | 1 | 1 | 0 | 1 | 1 | 1 | 0 | 1 | 1 | 1 | 8 |  |
| **Peripheral artery disease** | No peripheral artery disease | All-cause mortality | Zhu 2021 | 0 | 1 | 1 | 0 | 1 | 1 | 1 | 0 | 1 | 1 | 1 | 8 |  |
| **Demographic characteristics** | | | | | | | | | | | | | | | |  |
| **Latin American (NOAC)** | Non-Latin American (NOAC) | All-cause mortality | Su 2020 | 1 | 1 | 1 | 0 | 1 | 1 | 1 | 0 | 1 | 1 | 1 | 9 |  |
| **Latin American (Warfarin)** | Non-Latin American (Warfarin) | All-cause mortality | Su 2020 | 1 | 1 | 1 | 0 | 1 | 1 | 1 | 0 | 1 | 1 | 1 | 9 |  |
| **Latin American (NOAC+Warfarin)** | Non-Latin American (NOAC+Warfarin)= | All-cause mortality | Su 2020 | 1 | 1 | 1 | 0 | 1 | 1 | 1 | 0 | 1 | 1 | 1 | 9 |  |
| **High BMI** | Low BMI | All-cause mortality  Cardiovascular mortality | Liu 2020 | 1 | 1 | 1 | 0 | 1 | 1 | 1 | 0 | 1 | 1 | 1 | 9 |  |
| **Obesity (BMI ≥ 30)** | Normal BMI | All-cause mortality | Grymonprez 2021 | 0 | 1 | 1 | 0 | 1 | 1 | 1 | 0 | 1 | 1 | 1 | 8 |  |
| **Overweight (BMI 25-30)** | Normal BMI | All-cause mortality | Zhu 2016 | 0 | 1 | 1 | 0 | 1 | 1 | 1 | 0 | 1 | 1 | 1 | 8 |  |
| **Underweight (BMI < 18.5)** | Normal BMI | All-cause mortality | Grymonprez 2021 | 0 | 1 | 1 | 0 | 1 | 1 | 1 | 0 | 1 | 1 | 1 | 8 |  |
| **Smoking** | No smoking | All-cause mortality  Cardiovascular mortality | Zhu 2016 | 0 | 1 | 1 | 0 | 1 | 1 | 1 | 0 | 1 | 0 | 1 | 7 |  |
| **Men** | Women | All-cause mortality | Marzona 2018 | 0 | 1 | 1 | 1 | 1 | 1 | 1 | 0 | 1 | 1 | 1 | 9 |  |
| **Elderly group (＞75 years old)** | Younger group (<75 years old) | All-cause mortality | Li 2022 | 1 | 1 | 1 | 0 | 1 | 1 | 1 | 0 | 1 | 1 | 1 | 9 |  |
| **Surgery** | | | | | | | | | | | | | | | |  |
| **Catheter Ablation** | Non-catheter ablation | All-cause mortality | Shaik 2022 | 0 | 1 | 1 | 0 | 1 | 1 | 1 | 0 | 1 | 1 | 1 | 8 |  |
| **Ablation (RAF)** | Medical therapy (RAF) | Mortality | Kheshti 2023 | 0 | 1 | 1 | 0 | 1 | 1 | 1 | 0 | 1 | 1 | 1 | 8 |  |
| **Ablation (PeAF)** | Medical therapy (PeAF) | Mortality | Kheshti 2023 | 0 | 1 | 1 | 0 | 1 | 1 | 1 | 0 | 1 | 1 | 1 | 8 |  |
| **Ablation** | Drug therapy | All-cause mortality  Cardiac mortality | Zahoor 2023 | 1 | 1 | 1 | 1 | 1 | 1 | 1 | 0 | 1 | 1 | 1 | 10 |  |
| **Pharmacotherapy** | AVNA | Mortality | Chatterjee 2012 | 0 | 1 | 1 | 0 | 1 | 1 | 1 | 0 | 1 | 1 | 1 | 8 |  |
| **Biventricular pacing mode ungergoing AVNA** | Right ventricular pacing mode  undergoing AVNA | All-cause mortality  Cardiac mortality | Chatterjee 2012 | 0 | 1 | 1 | 1 | 1 | 1 | 1 | 0 | 1 | 1 | 1 | 9 |  |
| **Biatrial Maze** | Left Atrial Maze | Early death  Overall death | Zheng 2016 | 0 | 1 | 1 | 0 | 0 | 1 | 0 | 0 | 1 | 1 | 1 | 6 |  |
| **Biatrial ablation** | Isolated left atrial ablation | Mortality within 30 days  Late mortality | Li 2018 | 0 | 1 | 1 | 0 | 1 | 1 | 1 | 0 | 1 | 1 | 1 | 8 |  |
| **Catheter ablation (reduced LVEF)** | Medical therapy (reduced LVEF) | Mortality | Ravi 2022 | 0 | 1 | 1 | 0 | 1 | 1 | 1 | 0 | 1 | 1 | 1 | 8 |  |
| **Catheter ablation (preserved LVEF)** | Medical therapy (preserved LVEF) | Mortality | Ravi 2022 | 0 | 1 | 1 | 0 | 1 | 1 | 1 | 0 | 1 | 1 | 1 | 8 |  |
| **Catheter ablation (no prior AADs use)** | Medical therapy (no prior AADs use) | Mortality | Ravi 2022 | 0 | 1 | 1 | 0 | 1 | 1 | 1 | 0 | 1 | 1 | 1 | 8 |  |
| **Catheter Ablation (HF)** | Medical therapy (HF) | All-cause mortality | Asad 2019 | 0 | 1 | 1 | 0 | 1 | 1 | 1 | 0 | 1 | 1 | 1 | 8 |  |
| **Catheter Ablation (Non-HF)** | Medical therapy (Non-HF) | All-cause mortality | Asad 2019 | 0 | 1 | 1 | 0 | 1 | 1 | 1 | 0 | 1 | 1 | 1 | 8 |  |
| **Catheter Ablation (HF+Non-HF)** | Medical therapy (HF+Non-HF) | All-cause mortality | Asad 2019 | 0 | 1 | 1 | 0 | 1 | 1 | 1 | 0 | 1 | 1 | 1 | 8 |  |
| **Catheter Ablation** | Medical therapy (rate control) | All-cause mortality | Asad 2019 | 0 | 1 | 1 | 0 | 1 | 1 | 1 | 0 | 1 | 1 | 1 | 8 |  |
| **Catheter Ablation** | AAD | All-cause mortality | Asad 2019 | 0 | 1 | 1 | 0 | 1 | 1 | 1 | 0 | 1 | 1 | 1 | 8 |  |
| **LAAC during cardiac surgery** | No LAAC | Postoperative mortality  All-cause mortality > 2 years | Prasad 2022 | 0 | 1 | 1 | 0 | 1 | 1 | 1 | 0 | 1 | 1 | 1 | 8 |  |
| **Left atrial concomitant surgical ablation in cardiac surgery** | Cardiac surgery alone | 30-day mortality   Late mortality | Wang 2018 | 0 | 1 | 1 | 0 | 1 | 1 | 1 | 0 | 1 | 1 | 1 | 8 |  |
| **LAAO** | DOAC | Mortality | Abdelfattah 2023 | 1 | 1 | 1 | 0 | 1 | 0 | 1 | 1 | 1 | 1 | 1 | 9 |  |
| **LAAC** | OAC | All-cause mortality  Cardiovascular mortality | Franchin 2023 | 1 | 1 | 1 | 0 | 1 | 1 | 1 | 0 | 1 | 1 | 1 | 9 |  |
| **LAAO** | Warfarin | Mortality | Abdelfattah 2023 | 1 | 1 | 1 | 0 | 1 | 0 | 1 | 1 | 1 | 1 | 1 | 9 |  |
| **LAAO** | No LAAO | All-cause mortality | Nso 2022 | 0 | 1 | 1 | 0 | 1 | 1 | 1 | 0 | 1 | 1 | 1 | 8 |  |
| **Surgical ablation in cardiac Surgery** | No surgical ablation | All-cause mortality  All-cause ICU mortality during index hospitalization  All-cause hospital mortality during index hospitalization | McClure 2018 | 0 | 1 | 1 | 0 | 1 | 1 | 1 | 1 | 1 | 1 | 1 | 9 |  |
| **Others (other medications, disease characteristics, treatment strategies)** | | | | | | | | | | | | | | | |  |
| **RAAS blockers** | No RAAS blockers | All-cause mortality  Cardiovascular mortality | Chaugai 2016 | 0 | 1 | 1 | 0 | 1 | 1 | 1 | 0 | 1 | 1 | 1 | 8 |  |
| **SGLT2 inhibitors** | Placebo | All-cause mortality  Cardiovascular mortality | Zheng 2024 | 1 | 1 | 1 | 0 | 1 | 1 | 1 | 0 | 1 | 1 | 1 | 9 |  |
| **Statin (low-to-moderate intensity statin)** | No statin | All-cause mortality | Eun 2020 | 0 | 1 | 1 | 1 | 1 | 1 | 1 | 0 | 1 | 1 | 1 | 9 |  |
| **Statin (high intensity statin)** | No statin | All-cause mortality | Eun 2020 | 0 | 1 | 1 | 1 | 1 | 1 | 1 | 0 | 1 | 1 | 1 | 9 |  |
| **Statin (low-to-moderate intensity statin)** | Statin (high intensity statin) | All-cause mortality | Eun 2020 | 0 | 1 | 1 | 1 | 1 | 1 | 1 | 0 | 1 | 1 | 1 | 9 |  |
| **Statin** | No statin | All-cause mortality  Cardiovascular mortality | Pastori 2021 | 0 | 1 | 1 | 0 | 0 | 1 | 1 | 0 | 1 | 1 | 1 | 7 |  |
| **Asymptomatic AF** | Symptomatic AF | All-cause mortality  Cardiovascular mortality | Sgreccia 2021 | 0 | 1 | 1 | 0 | 1 | 1 | 1 | 0 | 1 | 1 | 1 | 8 |  |
| **Non-paroxysmal AF** | Paroxysmal AF | All-cause mortality | Mentel 2020 | 1 | 1 | 1 | 0 | 1 | 1 | 1 | 1 | 1 | 1 | 1 | 10 |  |
| **Paroxysmal atrial fibrillation** | Persistent atrial fibrillation | All-cause mortality | Zhang 2019 | 0 | 1 | 1 | 0 | 1 | 1 | 1 | 0 | 1 | 1 | 1 | 8 |  |
| **Exercise-based cardiac rehabilitation** | Usual care | All-cause mortality | Smart 2018 | 0 | 1 | 1 | 0 | 1 | 1 | 1 | 0 | 1 | 1 | 1 | 8 |  |
| **Early rhythm control** | rate control | All-cause mortality  Cardiovascular mortality | Han 2023 | 1 | 1 | 1 | 1 | 0 | 1 | 1 | 0 | 1 | 1 | 1 | 9 |  |
| **Rate control** | Rhythm control | All-cause mortality  Cardiovascular mortality | Depoorter 2020 | 1 | 1 | 1 | 0 | 1 | 1 | 1 | 0 | 1 | 1 | 1 | 9 |  |
| PeAF, Persistent atrial fibrillation; AF, Atrial fibrillation; RAF, Refractory atrial fibrillation; HF, Heart failure; AAD, Anti-arrhythmic Drugs; DOAC, Direct oral anticoagulant; NOAC, Non-vitamin K antagonist oral anticoagulant; OAC, Oral anticoagulant; CrCl, Creatinine clearance rate; BMI, Body mass index; TTR,Time in therapeutic range; TIA, Transient ischemic attack; VKA, Vitamin K antagonists; INR, International normalized ratio; RAAS, Renin-angiotensin-aldosterone system; SGLT2, Sodium-glucose transporter 2; AVNA, [Atrioventricular nodal ablation](https://pubmed.ncbi.nlm.nih.gov/28345365/); LVEF, Left ventricular ejection fractions; LAAC, Left atrial appendage closure; LAAO, Left atrial appendage occlusion; COPD, Chronic obstructive pulmonary disease; LA, Left atrial; LVH, Left ventricular hypertrophy; LD, low-dose; HD, high-dose. | | | | | | | | | | | | | | | | |
