# Supplementary material for: Factors associated with mortality in patients with atrial fibrillation: an umbrella review of systematic reviews and meta-analyses
Source: Front Cardiovasc Med. 2026 Oct 13;13:1877770. doi: 10.3389/fcvm.2026.1877770 (PMC13710006; doi:10.3389/fcvm.2026.1877770)
Supplement: Supplementary file 4 [file Table4.docx]

Table S4. GRADE classification of quality of evidence.

| Risk factors | Control | Outcomes | Included MA | No. of studies T/R/C/P | Risk of bias | Inconsistency | Indirectness | Imprecision | Publication bias | Plausible confounding | Magnitude of effect | Dose-response gradient | Quality |
| --- | --- | --- | --- | --- | --- | --- | --- | --- | --- | --- | --- | --- | --- |
| ***Significant associations*** | | | | | | | | | | | | | |
| **Anticoagulants or Antiplatelets** | | | | | | | | | | | | | |
| Adjusted-dose warfarin | Other antithrombotic treatment or placebo | All-cause mortality | Hart 2007 | 29/29/0/0 | Serious risk | Serious inconsistency | No serious indirectness | Serious imprecision | NA | Would not reduce effect | Yes | No | Low |
| Apixaban | Rivaroxaban | Mortality | Buckley 2022 | 11/0/9/2 | No serious risk | Serious inconsistency | No serious indirectness | Serious imprecision | NA | Would not reduce effect | No | No | Very low |
| Apixaban | Warfarin | All-cause mortality | Waranugraha 2021 | 6/0/0/6 | No serious risk | Serious inconsistency | No serious indirectness | Serious imprecision | Undetected | Would not reduce effect | No | No | Very low |
| Dabigatran 150mg | Warfarin | All-cause mortality | Escobar 2021 | 11/0/11/0 | No serious risk | Serious inconsistency | No serious indirectness | No serious imprecision | Strongly suspected | Would not reduce effect | No | No | Very low |
| Dabigatran | Warfarin | All-cause mortality | Waranugraha 2021 | 9/0/0/9 | No serious risk | Serious inconsistency | No serious indirectness | No serious imprecision | Undetected | Would not reduce effect | No | No | Very low |
| DOAC (Diabetes) | VKA (Diabetes) | Cardiovascular mortality | Abdool 2020 | 3/3/0/0 | No serious risk | Serious inconsistency | No serious indirectness | Serious imprecision | NA | Would not reduce effect | No | No | Low |
| DOAC (No Diabetes) | VKA (No Diabetes) | All-cause mortality | Abdool 2020 | 2/2/0/0 | No serious risk | Serious inconsistency | No serious indirectness | Serious imprecision | NA | Would not reduce effect | No | No | Low |
| DOAC | Warfarin | Mortality | Xu 2023 | 3/0/0/3 | No serious risk | No serious inconsistency | No serious indirectness | No serious imprecision | Strongly suspected | Would not reduce effect | Yes | No | Low |
| DOAC (BMI 25-30) | Warfarin (BMI 25-30) | All-cause mortality | Patel 2024 | 4/4/0/0 | No serious risk | No serious inconsistency | No serious indirectness | No serious imprecision | NA | Would not reduce effect | No | No | High |
| DOAC with polypharmacy (≥ 5 concomitant drugs) | Warfarin with polypharmacy (≥ 5 concomitant drugs) | Mortality | Kim 2019 | 4/4/0/0 | No serious risk | No serious inconsistency | No serious indirectness | Serious imprecision | Undetected | Would not reduce effect | No | No | Moderate |
| DOAC without amiodarone | Warfarin without amiodarone | Mortality | Kim 2019 | 4/4/0/0 | No serious risk | No serious inconsistency | No serious indirectness | Serious imprecision | Undetected | Would not reduce effect | No | No | Moderate |
| DOAC without P-glycoprotein inhibitor | Warfarin without P-glycoprotein inhibitor | Mortality | Kim 2019 | 4/4/0/0 | No serious risk | No serious inconsistency | No serious indirectness | Serious imprecision | Undetected | Would not reduce effect | No | No | Moderate |
| Edoxaban 30mg | Aspirin | Mortality | Blann 2015 | 8/8/0/0 | No serious risk | Serious inconsistency | No serious indirectness | Serious imprecision | NA | Would not reduce effect | No | No | Low |
| Edoxaban 30mg | Placebo | Mortality | Blann 2015 | 8/8/0/0 | No serious risk | Serious inconsistency | No serious indirectness | Serious imprecision | NA | Would not reduce effect | No | No | Low |
| Edoxaban 30mg | Warfarin | Mortality | Blann 2015 | 8/8/0/0 | No serious risk | Serious inconsistency | No serious indirectness | Serious imprecision | NA | Would not reduce effect | No | No | Low |
| Edoxaban 30mg with concomitant aspirin therapy | Warfarin with concomitant aspirin therapy | Vascular mortality | Bennaghmouch 2018 | 4/4/0/0 | No serious risk | Serious inconsistency | No serious indirectness | Serious imprecision | NA | Would not reduce effect | No | No | Low |
| Edoxaban 60mg | Placebo | Mortality | Blann 2015 | 8/8/0/0 | No serious risk | Serious inconsistency | No serious indirectness | Serious imprecision | NA | Would not reduce effect | No | No | Low |
| Edoxaban 60mg | Warfarin | Cardiovascular mortality | Aronow 2020 | 2/2/0/0 | No serious risk | Serious inconsistency | No serious indirectness | Serious imprecision | NA | Would not reduce effect | No | No | Low |
| Edoxaban | Warfarin | All-cause mortality | Waranugraha 2021 | 3/0/0/3 | No serious risk | Serious inconsistency | No serious indirectness | Serious imprecision | Undetected | Would not reduce effect | No | No | Very low |
| HD-Rivaroxaban | HD-Dabigatran | Mortality | Bai 2017 | 3/0/3/0 | No serious risk | No serious inconsistency | No serious indirectness | Serious imprecision | NA | Would not reduce effect | No | No | Very low |
| LD-Rivaroxaban | LD-Dabigatran | Mortality | Bai 2017 | 3/0/3/0 | No serious risk | No serious inconsistency | No serious indirectness | Serious imprecision | NA | Would not reduce effect | No | No | Very low |
| NOAC (TTR < 65%) | Warfarin (TTR < 65%) | Mortality | Gómez-Outes 2013 | 2/2/0/0 | No serious risk | No serious inconsistency | No serious indirectness | No serious imprecision | NA | Would not reduce effect | No | No | High |
| NOAC with concomitant aspirin therapy | Warfarin with concomitant aspirin therapy | Vascular mortality | Bennaghmouch 2018 | 4/4/0/0 | No serious risk | No serious inconsistency | No serious indirectness | No serious imprecision | NA | Would not reduce effect | No | No | High |
| NOAC (No previous stroke or TIA) | Warfarin (No previous stroke or TIA) | Mortality | Gómez-Outes 2013 | 3/3/0/0 | No serious risk | No serious inconsistency | No serious indirectness | Serious imprecision | NA | Would not reduce effect | No | No | Moderate |
| NOAC (non-Latin American) | Warfarin (non-Latin American) | All-cause mortality | Su 2020 | 3/3/0/0 | No serious risk | No serious inconsistency | No serious indirectness | Serious imprecision | Undetected | Would not reduce effect | No | No | Moderate |
| Non−ximelagatran NOAC | Control drugs | All-cause mortality | Liu 2014 | 12/12/0/0 | No serious risk | No serious inconsistency | No serious indirectness | No serious imprecision | Undetected | Would not reduce effect | No | No | High |
| VKA | Oral factor Xa inhibitors | All-cause mortality | Garg 2016 | 6/6/0/0 | No serious risk | No serious inconsistency | No serious indirectness | No serious imprecision | NA | Would not reduce effect | No | No | High |
| Therapeutic INR (ischemic stroke only) | Other antithrombotic categories (ischemic stroke only) | Early fatality (30 days) | Hannon 2015 | 8/0/8/0 | No serious risk | Serious inconsistency | No serious indirectness | No serious imprecision | Undetected | Would not reduce effect | Yes | No | Low |
| Therapeutic INR (ischemic stroke only) | Other antithrombotic categories (ischemic stroke only) | Late fatality (1 year) | Hannon 2015 | 4/0/4/0 | No serious risk | Serious inconsistency | No serious indirectness | No serious imprecision | Undetected | Would not reduce effect | Yes | No | Low |
| Warfarin | Placebo | All-cause mortality | Guimarães 2017 | 6/6/0/0 | No serious risk | Serious inconsistency | No serious indirectness | Serious imprecision | NA | Would not reduce effect | No | No | Low |
| **Antiarrhythmics** | | | | | | | | | | | | | |
| Class IA drugs | No treatment, placebo, drugs for rate control | All-cause mortality | Lafuente-Lafuente 2015 | 8/8/0/0 | Serious risk | No serious inconsistency | No serious indirectness | Serious imprecision | Strongly suspected | Would not reduce effect | Yes | No | Low |
| Digoxin | Beta-blockers | All-cause mortality | Trongtorsak 2021 | 4/2/2/0 | No serious risk | No serious inconsistency | No serious indirectness | No serious imprecision | NA | Would not reduce effect | No | No | Low |
| Digoxin | Calcium channel blockers | All-cause mortality | Trongtorsak 2021 | 3/2/1/0 | No serious risk | Serious inconsistency | No serious indirectness | Serious imprecision | NA | Would not reduce effect | No | No | Very low |
| Digoxin | No digoxin | All-cause mortality | Wang 2021 | 29/8/21/0 | No serious risk | Serious inconsistency | No serious indirectness | No serious imprecision | Undetected | Would not reduce effect | No | No | Very low |
| Digoxin | No digoxin | Cardiovascular mortality | Wang 2021 | 10/6/4/0 | No serious risk | Serious inconsistency | No serious indirectness | Serious imprecision | Undetected | Would not reduce effect | No | No | Very low |
| Digoxin | No digoxin | Sudden cardiac death | Wang 2021 | 5/3/2/0 | No serious risk | Serious inconsistency | No serious indirectness | No serious imprecision | Undetected | Would not reduce effect | No | No | Very low |
| Digoxin (AF without HF) | No digoxin (AF without HF) | All-cause mortality | Wang 2021 | 10/2/8/0 | No serious risk | Serious inconsistency | No serious indirectness | No serious imprecision | Undetected | Would not reduce effect | No | No | Very low |
| Dronedarone with digoxin | Placebo with digoxin | Arrhythmic mortality | Boriani 2019 | 10/10/0/0 | Serious risk | Serious inconsistency | No serious indirectness | Serious imprecision | NA | Would not reduce effect | Yes | No | Low |
| Dronedarone (Digoxin at baseline) | Placebo (Digoxin at baseline) | All-cause mortality | Boriani 2019 | 10/10/0/0 | Serious risk | Serious inconsistency | No serious indirectness | Serious imprecision | NA | Would not reduce effect | Yes | No | Low |
| Dronedarone (Digoxin at baseline) | Placebo (Digoxin at baseline) | Arrhythmic mortality | Boriani 2019 | 10/10/0/0 | Serious risk | Serious inconsistency | No serious indirectness | Serious imprecision | NA | Would not reduce effect | Yes | No | Low |
| Sotalol | Placebo | All-cause mortality | Lafuente-Lafuente 2015 | 13/13/0/0 | Serious risk | No serious inconsistency | No serious indirectness | Serious imprecision | Strongly suspected | Would not reduce effect | Yes | No | Low |
| Sotalol | Placebo or no treatment | All-cause mortality | Valembois 2019 | 5/5/0/0 | Serious risk | No serious inconsistency | No serious indirectness | Serious imprecision | Undetected | Would not reduce effect | Yes | No | Moderate |
| Sotalol (PeAF) | Placebo or no treatment (PeAF) | All-cause mortality | Valembois 2019 | 3/3/0/0 | Serious risk | No serious inconsistency | No serious indirectness | Serious imprecision | Undetected | Would not reduce effect | Yes | No | Moderate |
| **Comorbidities** | | | | | | | | | | | | | |
| Diabetes | No diabetes | All-cause mortality | Xu 2022 | 19/6/0/13 | No serious risk | Serious inconsistency | No serious indirectness | Serious imprecision | Undetected | Would not reduce effect | No | No | Very low |
| Diabetes | No diabetes | Cardiovascular mortality | Xu 2022 | 10/1/0/9 | No serious risk | No serious inconsistency | No serious indirectness | Serious imprecision | Strongly suspected | Would not reduce effect | No | No | Very low |
| Anemia | No anemia | All-cause mortality | Tu 2020 | 10/1/9/0 | Serious risk | Serious inconsistency | No serious indirectness | No serious imprecision | Strongly suspected | Would not reduce effect | No | No | Very low |
| Cancer | No cancer | All-cause mortality | Balomenakis 2023 | 12/2/9/1 | Serious risk | Serious inconsistency | No serious indirectness | No serious imprecision | Strongly suspected | Would not reduce effect | Yes | No | Very low |
| Cancer | No cancer | Non-Cardiovascular mortality | Balomenakis 2023 | 6/1/4/1 | Serious risk | Serious inconsistency | No serious indirectness | No serious imprecision | Strongly suspected | Would not reduce effect | Yes | No | Very low |
| COPD | No COPD | All-cause mortality | Romiti 2021 | 14/1/13/0 | Serious risk | Serious inconsistency | No serious indirectness | No serious imprecision | Strongly suspected | Would not reduce effect | Yes | No | Very low |
| COPD | No COPD | Cardiovascular mortality | Romiti 2021 | 10/1/9/0 | Serious risk | Serious inconsistency | No serious indirectness | No serious imprecision | Strongly suspected | Would not reduce effect | No | No | Very low |
| Frailty | No frailty | All-cause mortality | He 2022 | 8/0/8/0 | No serious risk | Serious inconsistency | No serious indirectness | No serious imprecision | NA | Would not reduce effect | Yes | No | Low |
| LVH | No LVH | All-cause mortality | Xiang 2021 | 3/2/1/0 | No serious risk | Serious inconsistency | No serious indirectness | No serious imprecision | Undetected | Would not reduce effect | No | No | Very low |
| Peripheral artery disease | No peripheral artery disease | All-cause mortality | Zhu 2021 | 5/0/5/0 | No serious risk | No serious inconsistency | No serious indirectness | No serious imprecision | Undetected | Would not reduce effect | No | No | Low |
| **Demographic characteristics** | | | | | | | | | | | | | |
| High BMI | Low BMI | All-cause mortality | Liu 2020 | 13/8/5/0 | No serious risk | Serious inconsistency | No serious indirectness | No serious imprecision | Undetected | Would not reduce effect | No | No | Very low |
| High BMI | Low BMI | Cardiovascular mortality | Liu 2020 | 7/3/4/0 | No serious risk | Serious inconsistency | No serious indirectness | Serious imprecision | Undetected | Would not reduce effect | No | No | Very low |
| Latin American (Warfarin) | Non-Latin American (Warfarin) | All-cause mortality | Su 2020 | 2/2/0/0 | No serious risk | No serious inconsistency | No serious indirectness | No serious imprecision | Undetected | Would not reduce effect | No | No | High |
| Latin American (NOAC) | Non-Latin American (NOAC) | All-cause mortality | Su 2020 | 4/4/0/0 | No serious risk | No serious inconsistency | No serious indirectness | No serious imprecision | Undetected | Would not reduce effect | No | No | High |
| Latin American (NOAC+Warfarin) | Non-Latin American (NOAC+Warfarin)) | All-cause mortality | Su 2020 | 6/6/0/0 | No serious risk | No serious inconsistency | No serious indirectness | No serious imprecision | Undetected | Would not reduce effect | No | No | High |
| Overweight (BMI 25-30) | Normal BMI | All-cause mortality | Zhu 2016 | 7/3/4/0 | No serious risk | Serious inconsistency | No serious indirectness | Serious imprecision | Undetected | Would not reduce effect | No | No | Very low |
| Obesity (BMI ≥ 30) | Normal BMI | All-cause mortality | Grymonprez 2021 | 5/2/3/0 | No serious risk | Serious inconsistency | No serious indirectness | No serious imprecision | Undetected | Would not reduce effect | No | No | Very low |
| Smoking | No smoking | All-cause mortality | Zhu 2016 | 6/0/6/0 | No serious risk | Serious inconsistency | No serious indirectness | No serious imprecision | NA | Would not reduce effect | No | No | Very low |
| Smoking | No smoking | Cardiovascular mortality | Zhu 2016 | 4/0/4/0 | No serious risk | No serious inconsistency | No serious indirectness | No serious imprecision | NA | Would not reduce effect | No | No | Very low |
| Underweight (BMI < 18.5) | Normal BMI | All-cause mortality | Grymonprez 2021 | 4/1/3/0 | No serious risk | Serious inconsistency | No serious indirectness | No serious imprecision | Undetected | Would not reduce effect | Yes | No | Low |
| Elderly group (<75 years old) | Younger group (＞75 years old) | All-cause mortality | Li 2022 | 14/0/14/0 | No serious risk | Serious inconsistency | No serious indirectness | Serious imprecision | Undetected | Would not reduce effect | Yes | No | Very low |
| **Surgery** | | | | | | | | | | | | | |
| Ablation | Drug therapy | Cardiac mortality | Zahoor 2023 | 4/0/4/0 | No serious risk | No serious inconsistency | No serious indirectness | No serious imprecision | Strongly suspected | Would not reduce effect | Yes | No | Low |
| Ablation | Drug therapy | All-cause mortality | Zahoor 2023 | 6/1/5/0 | No serious risk | Serious inconsistency | No serious indirectness | Serious imprecision | Strongly suspected | Would not reduce effect | No | No | Very low |
| Catheter ablation | Non-catheter ablation | All-cause mortality | Shaik 2022 | 8/1/7/0 | No serious risk | Serious inconsistency | No serious indirectness | No serious imprecision | Undetected | Would not reduce effect | No | No | Very low |
| Catheter ablation (HF) | Medical therapy (HF) | All-cause mortality | Asad 2019 | 4/4/0/0 | No serious risk | No serious inconsistency | No serious indirectness | No serious imprecision | NA | Would not reduce effect | No | No | High |
| Catheter ablation (HF+non-HF) | Medical therapy (HF+non-HF) | All-cause mortality | Asad 2019 | 9/9/0/0 | No serious risk | No serious inconsistency | No serious indirectness | Serious imprecision | NA | Would not reduce effect | No | No | Moderate |
| Catheter ablation | AAD | All-cause mortality | Asad 2019 | 4/4/0/0 | No serious risk | No serious inconsistency | No serious indirectness | Serious imprecision | NA | Would not reduce effect | Yes | No | High |
| Catheter ablation (reduced LVEF) | Medical therapy (reduced LVEF) | Mortality | Ravi 2022 | 5/5/0/0 | Serious risk | No serious inconsistency | No serious indirectness | Serious imprecision | Undetected | Would not reduce effect | No | No | Low |
| LAAC | OAC | All-cause mortality | Franchin 2023 | 8/2/6/0 | No serious risk | Serious inconsistency | No serious indirectness | No serious imprecision | NA | Would not reduce effect | No | No | Very low |
| LAAC | OAC | Cardiovascular mortality | Franchin 2023 | 5/2/3/0 | No serious risk | No serious inconsistency | No serious indirectness | No serious imprecision | NA | Would not reduce effect | No | No | Low |
| LAAC during cardiac surgery | No LAAC | All-cause mortality > 2 years | Prasad 2022 | 8/2/6/0 | Serious risk | Serious inconsistency | No serious indirectness | Serious imprecision | Strongly suspected | Would not reduce effect | No | No | Very low |
| LAAO | DOAC | Mortality | Abdelfattah 2023 | 37/37/0/0 | Serious risk | Serious inconsistency | No serious indirectness | Serious imprecision | NA | Would not reduce effect | No | No | Very low |
| LAAO | Warfarin | Mortality | Abdelfattah 2023 | 37/37/0/0 | No serious risk | Serious inconsistency | No serious indirectness | Serious imprecision | NA | Would not reduce effect | No | No | Low |
| **Others (other medications, disease characteristics, treatment strategies)** | | | | | | | | | | | | | |
| SGLT2 inhibitors | Placebo | Cardiovascular mortality | Zheng 2024 | 5/5/0/0 | No serious risk | No serious inconsistency | No serious indirectness | Serious imprecision | NA | Would not reduce effect | No | No | Moderate |
| Statin (low-to-moderate intensity statin) | No statin | All-cause mortality | Eun 2020 | 2/0/2/0 | Serious risk | No serious inconsistency | No serious indirectness | Serious imprecision | NA | Would not reduce effect | No | No | Very low |
| Statin (high intensity statin) | No statin | All-cause mortality | Eun 2020 | 2/0/2/0 | Serious risk | Serious inconsistency | No serious indirectness | No serious imprecision | NA | Would not reduce effect | Yes | No | Very low |
| Statin (high intensity statin) | Statin (low-to-moderate intensity statin) | All-cause mortality | Eun 2020 | 2/0/2/0 | Serious risk | Serious inconsistency | No serious indirectness | Serious imprecision | NA | Would not reduce effect | No | No | Very low |
| Statin | No statin | All-cause mortality | Pastori 2021 | 12/1/11/0 | Serious risk | Serious inconsistency | No serious indirectness | No serious imprecision | NA | Would not reduce effect | No | No | Very low |
| Statin | No statin | Cardiovascular mortality | Pastori 2021 | 3/1/2/0 | Serious risk | Serious inconsistency | No serious indirectness | Serious imprecision | NA | Would not reduce effect | No | No | Very low |
| Paroxysmal AF | Persistent AF | All-cause mortality | Zhang 2019 | 4/4/0/0 | No serious risk | No serious inconsistency | No serious indirectness | No serious imprecision | Undetected | Would not reduce effect | No | No | High |
| Non-paroxysmal AF | Paroxysmal AF | All-cause mortality | Mentel 2020 | 13/0/13/0 | Serious risk | Serious inconsistency | No serious indirectness | No serious imprecision | Undetected | Would not reduce effect | No | No | Very low |
| Early rhythm control | Rate control | All-cause mortality | Han 2023 | 6/2/4/0 | Serious risk | Serious inconsistency | No serious indirectness | No serious imprecision | NA | Would not reduce effect | No | No | Very low |
| Early rhythm control | Rate control | Cardiovascular mortality | Han 2023 | 3/1/2/0 | Serious risk | No serious inconsistency | No serious indirectness | No serious imprecision | NA | Would not reduce effect | No | No | Very low |
| ***Non-significant associations*** | | | | | | | | | | | | | |
| **Anticoagulants or Antiplatelets** | | | | | | | | | | | | | |
| Adjusted-dose warfarin | Fixed low-dose warfarin | Mortality | Lip 2006 | 4/4/0/0 | No serious risk | Serious inconsistency | No serious indirectness | Serious imprecision | Undetected | Would not reduce effect | No | No | Low |
| Apixaban | Dabigatran | Mortality | Buckley 2022 | 9/0/7/2 | Serious risk | Serious inconsistency | No serious indirectness | Serious imprecision | NA | Would not reduce effect | No | No | Very low |
| Apixaban | Dabigatran 110mg | All-cause mortality | Mitchell 2013 | 3/3/0/0 | Serious risk | Serious inconsistency | No serious indirectness | Serious imprecision | NA | Would not reduce effect | No | No | Very low |
| Apixaban | Dabigatran 150mg | All-cause mortality | Mitchell 2013 | 3/3/0/0 | Serious risk | Serious inconsistency | No serious indirectness | Serious imprecision | NA | Would not reduce effect | No | No | Very low |
| Apixaban with concomitant aspirin therapy | Warfarin with concomitant aspirin therapy | Vascular mortality | Bennaghmouch 2018 | 4/4/0/0 | No serious risk | Serious inconsistency | No serious indirectness | Serious imprecision | NA | Would not reduce effect | No | No | Low |
| Apixaban–dronedarone combination | Warfarin–dronedarone combination | All-cause mortality | Boriani 2019 | 10/10/0/0 | Serious risk | Serious inconsistency | No serious indirectness | Serious imprecision | NA | Would not reduce effect | No | No | Very low |
| Aspirin or Clopidogrel | Warfarin | All-cause mortality | Melkonian 2017 | 7/5/2/0 | Serious risk | Serious inconsistency | No serious indirectness | Serious imprecision | Undetected | Would not reduce effect | No | No | Very low |
| Dabigatran 110mg | Warfarin | All-cause mortality | Escobar 2021 | 7/0/7/0 | No serious risk | Serious inconsistency | No serious indirectness | Serious imprecision | Strongly suspected | Would not reduce effect | No | No | Very low |
| Dabigatran 110mg with concomitant aspirin therapy | Warfarin with concomitant aspirin therapy | Vascular mortality | Bennaghmouch 2018 | 4/4/0/0 | No serious risk | Serious inconsistency | No serious indirectness | Serious imprecision | NA | Would not reduce effect | No | No | Low |
| Dabigatran 150mg | Dabigatran 110mg | All-cause mortality | Mitchell 2013 | 3/3/0/0 | Serious risk | Serious inconsistency | No serious indirectness | Serious imprecision | NA | Would not reduce effect | No | No | Very low |
| Dabigatran 150mg with concomitant aspirin therapy | Warfarin with concomitant aspirin therapy | Vascular mortality | Bennaghmouch 2018 | 4/4/0/0 | No serious risk | Serious inconsistency | No serious indirectness | Serious imprecision | NA | Would not reduce effect | No | No | Low |
| Dabigatran (unespecified dose but not 75mg ) | Warfarin | All-cause mortality | Escobar 2021 | 4/0/4/0 | No serious risk | Serious inconsistency | No serious indirectness | Serious imprecision | Strongly suspected | Would not reduce effect | No | No | Very low |
| DOAC (BMI 18.5-25) | Warfarin (BMI 18.5-25) | All-cause mortality | Patel 2024 | 4/4/0/0 | No serious risk | No serious inconsistency | No serious indirectness | Serious imprecision | NA | Would not reduce effect | No | No | Moderate |
| DOAC (BMI 30-35) | Warfarin (BMI 30-35) | All-cause mortality | Patel 2024 | 4/4/0/0 | No serious risk | No serious inconsistency | No serious indirectness | Serious imprecision | NA | Would not reduce effect | No | No | Moderate |
| DOAC (BMI 35-40) | Warfarin (BMI 35-40) | All-cause mortality | Patel 2024 | 4/4/0/0 | No serious risk | No serious inconsistency | No serious indirectness | Serious imprecision | NA | Would not reduce effect | No | No | Moderate |
| DOAC (BMI > 30) | Warfarin (BMI > 30) | All-cause mortality | Malik 2020 | 5/5/0/0 | Serious risk | No serious inconsistency | No serious indirectness | Serious imprecision |  | Would not reduce effect | No | No | Low |
| DOAC (BMI ≥ 40) | Warfarin (BMI ≥ 40) | All-cause mortality | Patel 2024 | 4/4/0/0 | No serious risk | No serious inconsistency | No serious indirectness | Serious imprecision | NA | Would not reduce effect | No | No | Moderate |
| DOAC (Diabetes) | VKA (No Diabetes) | All-cause mortality | Abdool 2020 | 2/2/0/0 | No serious risk | Serious inconsistency | No serious indirectness | Serious imprecision | NA | Would not reduce effect | No | No | Low |
| DOAC (No Diabetes) | VKA (No Diabetes) | Cardiovascular mortality | Abdool 2020 | 3/3/0/0 | No serious risk | Serious inconsistency | No serious indirectness | Serious imprecision | NA | Would not reduce effect | No | No | Low |
| DOAC with fewer concomitant drugs (< 5 concomitant drugs) | Warfarin with fewer concomitant drugs (< 5 concomitant drugs) | Mortality | Kim 2019 | 4/4/0/0 | No serious risk | No serious inconsistency | No serious indirectness | Serious imprecision | Undetected | Would not reduce effect | No | No | Moderate |
| DOAC with amiodarone | Warfarin with amiodarone | Mortality | Kim 2019 | 4/4/0/0 | No serious risk | No serious inconsistency | No serious indirectness | Serious imprecision | Undetected | Would not reduce effect | No | No | Moderate |
| DOAC with P-glycoprotein inhibitor | Warfarin with P-glycoprotein inhibitor | Mortality | Kim 2019 | 4/4/0/0 | No serious risk | No serious inconsistency | No serious indirectness | Serious imprecision | Undetected | Would not reduce effect | No | No | Moderate |
| Edoxaban 30mg (Crcl 30-50) | Warfarin (Crcl 30-50) | Mortality | Wang 2023 | 2/0/2/0 | No serious risk | Serious inconsistency | No serious indirectness | Serious imprecision | NA | Would not reduce effect | No | No | Very low |
| Edoxaban 60mg (Crcl 50-95) | Warfarin (Crcl 50-95) | Mortality | Wang 2023 | 3/0/3/0 | No serious risk | Serious inconsistency | No serious indirectness | Serious imprecision | NA | Would not reduce effect | No | No | Very low |
| Edoxaban 60mg (Crcl > 95) | Warfarin (Crcl > 95) | Mortality | Wang 2023 | 2/0/2/0 | No serious risk | No serious inconsistency | No serious indirectness | Serious imprecision | NA | Would not reduce effect | No | No | Very low |
| Edoxaban 30mg | Edoxaban 60mg | All-cause mortality | Chen 2015 | 4/4/0/0 | No serious risk | No serious inconsistency | No serious indirectness | Serious imprecision | Undetected | Would not reduce effect | No | No | Moderate |
| Edoxaban 30mg | Aspirin and Clopidogrel | Mortality | Blann 2015 | 8/8/0/0 | No serious risk | Serious inconsistency | No serious indirectness | Serious imprecision | NA | Would not reduce effect | No | No | Low |
| Edoxaban 60mg | Apixaban | All-cause mortality | Aronow 2020 | 23/23/0/0 | No serious risk | Serious inconsistency | No serious indirectness | Serious imprecision | NA | Would not reduce effect | No | No | Low |
| Edoxaban 60mg | Aspirin | Mortality | Blann 2015 | 8/8/0/0 | No serious risk | Serious inconsistency | No serious indirectness | Serious imprecision | NA | Would not reduce effect | No | No | Low |
| Edoxaban 60mg | Aspirin and Clopidogrel | Mortality | Blann 2015 | 8/8/0/0 | No serious risk | Serious inconsistency | No serious indirectness | Serious imprecision | NA | Would not reduce effect | No | No | Low |
| Edoxaban 60mg | Dabigatran 150mg | All-cause mortality | Aronow 2020 | 23/23/0/0 | No serious risk | Serious inconsistency | No serious indirectness | Serious imprecision | NA | Would not reduce effect | No | No | Low |
| Edoxaban 60mg | Warfarin | All-cause mortality | Chen 2015 | 4/4/0/0 | No serious risk | No serious inconsistency | No serious indirectness | Serious imprecision | Undetected | Would not reduce effect | No | No | Moderate |
| Edoxaban 60mg | Rivaroxaban | All-cause mortality | Aronow 2020 | 23/23/0/0 | No serious risk | Serious inconsistency | No serious indirectness | Serious imprecision | NA | Would not reduce effect | No | No | Low |
| Edoxaban 60mg with concomitant aspirin therapy | Warfarin with concomitant aspirin therapy | Vascular mortality | Bennaghmouch 2018 | 4/4/0/0 | No serious risk | Serious inconsistency | No serious indirectness | Serious imprecision | NA | Would not reduce effect | No | No | Low |
| NOAC (TTR > 65%) | Warfarin (TTR > 65%) | Mortality | Gómez-Outes 2013 | 2/2/0/0 | No serious risk | No serious inconsistency | No serious indirectness | Serious imprecision | NA | Would not reduce effect | No | No | Moderate |
| NOAC (Latin American) | Warfarin (Latin American) | All-cause mortality | Su 2020 | 3/3/0/0 | No serious risk | No serious inconsistency | No serious indirectness | Serious imprecision | Undetected | Would not reduce effect | No | No | Moderate |
| NOAC (Previous stroke or TIA) | Warfarin (Previous stroke or TIA) | Mortality | Gómez-Outes 2013 | 3/3/0/0 | No serious risk | No serious inconsistency | No serious indirectness | Serious imprecision | NA | Would not reduce effect | No | No | Moderate |
| OAC | Aspirin | Mortality | Van Walraven 2002 | 6/6/0/0 | Serious risk | Serious inconsistency | No serious indirectness | Serious imprecision | NA | Would not reduce effect | No | No | Very low |
| OAC | Aspirin | Vascular mortality | Van Walraven 2002 | 6/6/0/0 | Serious risk | Serious inconsistency | No serious indirectness | Serious imprecision | NA | Would not reduce effect | No | No | Very low |
| OAC | Antiplatelet, placebo, or no antithrombotic treatment | All-cause mortality | Reddin 2021 | 14/14/0/0 | Serious risk | No serious inconsistency | No serious indirectness | Serious imprecision | Undetected | Would not reduce effect | No | No | Low |
| ProPeAFenone | Placebo or no treatment | All-cause mortality | Valembois 2019 | 2/2/0/0 | Serious risk | No serious inconsistency | No serious indirectness | Serious imprecision | Undetected | Would not reduce effect | No | No | Low |
| Rivaroxaban | Dabigatran | All-cause mortality | Menichelli 2021 | 6/0/6/0 | No serious risk | Serious inconsistency | No serious indirectness | Serious imprecision | Strongly suspected | Would not reduce effect | No | No | Very low |
| Rivaroxaban | Warfarin | All-cause mortality | Waranugraha 2021 | 7/0/0/7 | No serious risk | Serious inconsistency | No serious indirectness | Serious imprecision | Undetected | Would not reduce effect | No | No | Very low |
| Rivaroxaban new-users/ switchers | Warfarin | Mortality | Bai 2017 | 2/0/2/0 | No serious risk | Serious inconsistency | No serious indirectness | Serious imprecision | NA | Would not reduce effect | No | No | Very low |
| Rivaroxaban with concomitant aspirin therapy | Warfarin with concomitant aspirin therapy | Vascular mortality | Bennaghmouch 2018 | 4/4/0/0 | No serious risk | Serious inconsistency | No serious indirectness | Serious imprecision | NA | Would not reduce effect | No | No | Very low |
| Warfarin | Antiplatelet therapy | All-cause mortality | Shi 2015 | 9/9/0/0 | No serious risk | No serious inconsistency | No serious indirectness | Serious imprecision | Undetected | Would not reduce effect | No | No | Moderate |
| Warfarin | Aspirin | All-cause mortality | Lip 2006 | 5/5/0/0 | No serious risk | Serious inconsistency | No serious indirectness | Serious imprecision | Undetected | Would not reduce effect | No | No | Low |
| Ximelagatran | Control drugs | All-cause mortality | Liu 2014 | 4/4/0/0 | No serious risk | No serious inconsistency | No serious indirectness | Serious imprecision | Undetected | Would not reduce effect | No | No | Moderate |
| **Antiarrhythmics** | | | | | | | | | | | | | |
| Amiodarone | Class I antiarrhythmics | All-cause mortality | Lafuente-Lafuente 2015 | 5/5/0/0 | Serious risk | Serious inconsistency | No serious indirectness | Serious imprecision | Strongly suspected | Would not reduce effect | No | No | Very low |
| Amiodarone | Placebo | All-cause mortality | Lafuente-Lafuente 2015 | 5/5/0/0 | Serious risk | No serious inconsistency | No serious indirectness | Serious imprecision | Strongly suspected | Would not reduce effect | No | No | Very low |
| Amiodarone | Placebo or no treatment | All-cause mortality | Valembois 2019 | 2/2/0/0 | Serious risk | No serious inconsistency | No serious indirectness | Serious imprecision | Undetected | Would not reduce effect | No | No | Low |
| Amiodarone (PeAF) | Placebo or no treatment (PeAF) | All-cause mortality | Valembois 2019 | 2/2/0/0 | Serious risk | No serious inconsistency | No serious indirectness | Serious imprecision | Undetected | Would not reduce effect | No | No | Low |
| Amiodarone | Placebo or rate control drug | All-cause mortality | Doyle 2009 | 12/12/0/0 | Serious risk | No serious inconsistency | No serious indirectness | Serious imprecision | Strongly suspected | Would not reduce effect | No | No | Very low |
| Amiodarone | Dronedarone | All-cause mortality at 12 months | Sullivan 2013 | 35/35/0/0 | Serious risk | Serious inconsistency | No serious indirectness | Serious imprecision | Strongly suspected | Would not reduce effect | No | No | Very low |
| Amiodarone | Sotalol | All-cause mortality | Lafuente-Lafuente 2015 | 6/6/0/0 | Serious risk | Serious inconsistency | No serious indirectness | Serious imprecision | Strongly suspected | Would not reduce effect | No | No | Very low |
| Azimilide | Placebo | All-cause mortality | Lafuente-Lafuente 2015 | 5/5/0/0 | Serious risk | No serious inconsistency | No serious indirectness | Serious imprecision | Strongly suspected | Would not reduce effect | No | No | Very low |
| Azimilide | No treatment, placebo, drugs for rate control | All-cause mortality | Lafuente-Lafuente 2015 | 5/5/0/0 | Serious risk | No serious inconsistency | No serious indirectness | Serious imprecision | Strongly suspected | Would not reduce effect | No | No | Very low |
| Beta-blockers | No antiarrhythmic treatment | All-cause mortality | Lafuente-Lafuente 2012 | 2/2/0/0 | Serious risk | Serious inconsistency | No serious indirectness | Serious imprecision | Strongly suspected | Would not reduce effect | No | No | Very low |
| Beta-blockers | Calcium channel blockers | All-cause mortality | Trongtorsak 2021 | 4/0/4/0 | Serious risk | Serious inconsistency | No serious indirectness | Serious imprecision | NA | Would not reduce effect | No | No | Very low |
| Beta-blockers using (COPD) | No beta-blockers using (COPD) | All-cause mortality | Romiti 2021 | 9/1/8/0 | Serious risk | Serious inconsistency | No serious indirectness | Serious imprecision | Strongly suspected | Would not reduce effect | No | No | Very low |
| Beta-blockers using (COPD) | No beta-blockers using (COPD) | Cardiovascular mortality | Romiti 2021 | 8/1/7/0 | Serious risk | Serious inconsistency | No serious indirectness | Serious imprecision | Strongly suspected | Would not reduce effect | No | No | Very low |
| Beta-blockers using (No COPD) | No beta-blockers using (No COPD) | All-cause mortality | Romiti 2021 | 9/1/8/0 | Serious risk | Serious inconsistency | No serious indirectness | Serious imprecision | Strongly suspected | Would not reduce effect | No | No | Very low |
| Beta-blockers using (No COPD) | No beta-blockers using (No COPD) | Cardiovascular mortality | Romiti 2021 | 8/1/7/0 | Serious risk | Serious inconsistency | No serious indirectness | Serious imprecision | Strongly suspected | Would not reduce effect | No | No | Very low |
| Class IA drugs (PeAF) | No treatment, placebo, drugs for rate control (PeAF) | All-cause mortality | Lafuente-Lafuente 2015 | 6/6/0/0 | Serious risk | No serious inconsistency | No serious indirectness | Serious imprecision | Strongly suspected | Would not reduce effect | No | No | Very low |
| Class IB drugs | No treatment, placebo, drugs for rate control | All-cause mortality | Lafuente-Lafuente 2015 | 2/2/0/0 | Serious risk | Serious inconsistency | No serious indirectness | Serious imprecision | Strongly suspected | Would not reduce effect | No | No | Very low |
| Class IC drugs | No treatment, placebo, drugs for rate control | All-cause mortality | Lafuente-Lafuente 2015 | 10/10/0/0 | Serious risk | No serious inconsistency | No serious indirectness | Serious imprecision | Strongly suspected | Would not reduce effect | No | No | Very low |
| Class I drugs (PeAF) | No treatment, placebo, drugs for rate control (PeAF) | All-cause mortality | Lafuente-Lafuente 2015 | 7/7/0/0 | Serious risk | No serious inconsistency | No serious indirectness | Serious imprecision | Strongly suspected | Would not reduce effect | No | No | Very low |
| Class I drugs | No treatment, placebo, drugs for rate control | All-cause mortality | Lafuente-Lafuente 2015 | 11/11/0/0 | Serious risk | No serious inconsistency | No serious indirectness | Serious imprecision | Strongly suspected | Would not reduce effect | No | No | Very low |
| Class I other drugs (aprindine, bidisomide, pilsicainide) | No treatment, placebo, drugs for rate control | All-cause mortality | Lafuente-Lafuente 2015 | 2/2/0/0 | Serious risk | No serious inconsistency | No serious indirectness | Serious imprecision | Strongly suspected | Would not reduce effect | No | No | Very low |
| Class II drugs (PeAF) | No treatment, placebo, drugs for rate control (PeAF) | All-cause mortality | Lafuente-Lafuente 2015 | 2/2/0/0 | Serious risk | Serious inconsistency | No serious indirectness | Serious imprecision | Strongly suspected | Would not reduce effect | No | No | Very low |
| Class III drugs | Placebo | All-cause mortality | Lafuente-Lafuente 2015 | 17/17/0/0 | Serious risk | No serious inconsistency | No serious indirectness | Serious imprecision | Strongly suspected | Would not reduce effect | No | No | Very low |
| Class III drugs | class I drugs | All-cause mortality | Lafuente-Lafuente 2015 | 8/8/0/0 | Serious risk | No serious inconsistency | No serious indirectness | Serious imprecision | Strongly suspected | Would not reduce effect | No | No | Very low |
| Class III drugs (PeAF) | No treatment, placebo, drugs for rate control (PeAF) | All-cause mortality | Lafuente-Lafuente 2015 | 13/13/0/0 | Serious risk | No serious inconsistency | No serious indirectness | Serious imprecision | Strongly suspected | Would not reduce effect | No | No | Very low |
| Digoxin | No digoxin | Non-Cardiovascular mortality | Wang 2021 | 6/5/1/0 | No serious risk | Serious inconsistency | No serious indirectness | Serious imprecision | Undetected | Would not reduce effect | No | No | Very low |
| Digoxin (AF with HF) | No digoxin (AF with HF) | All-cause mortality | Wang 2021 | 14/3/11/0 | No serious risk | Serious inconsistency | No serious indirectness | Serious imprecision | Undetected | Would not reduce effect | No | No | Very low |
| Disopyramide | Placebo or no treatment | All-cause mortality | Lafuente-Lafuente 2015 | 2/2/0/0 | Serious risk | Serious inconsistency | No serious indirectness | Serious imprecision | Strongly suspected | Would not reduce effect | No | No | Very low |
| Disopyramide | Other class I drugs | All-cause mortality | Lafuente-Lafuente 2015 | 2/2/0/0 | Serious risk | Serious inconsistency | No serious indirectness | Serious imprecision | Strongly suspected | Would not reduce effect | No | No | Very low |
| Dronedarone | Placebo | All-cause mortality | Boriani 2019 | 10/10/0/0 | Serious risk | Serious inconsistency | No serious indirectness | Serious imprecision | NA | Would not reduce effect | No | No | Very low |
| Dronedarone | Placebo (No digoxin) | All-cause mortality | Boriani 2019 | 10/10/0/0 | Serious risk | Serious inconsistency | No serious indirectness | Serious imprecision | NA | Would not reduce effect | No | No | Very low |
| Dronedarone | Placebo (No digoxin) | Arrhythmic mortality | Boriani 2019 | 10/10/0/0 | Serious risk | Serious inconsistency | No serious indirectness | Serious imprecision | NA | Would not reduce effect | No | No | Very low |
| Dronedarone | Placebo or active control | All-cause mortality | Diemberger 2017 | 11/11/0/0 | No serious risk | Serious inconsistency | No serious indirectness | Serious imprecision | NA | Would not reduce effect | No | No | Low |
| Dronedarone | Placebo or active control | Cardiac mortality | Diemberger 2017 | 11/11/0/0 | No serious risk | Serious inconsistency | No serious indirectness | Serious imprecision | NA | Would not reduce effect | No | No | Low |
| Dronedarone | Placebo or no treatment | All-cause mortality | Valembois 2019 | 3/3/0/0 | Serious risk | No serious inconsistency | No serious indirectness | Serious imprecision | Undetected | Would not reduce effect | No | No | Low |
| Dofetilide | Placebo | All-cause mortality | Lafuente-Lafuente 2015 | 3/3/0/0 | Serious risk | No serious inconsistency | No serious indirectness | Serious imprecision | Strongly suspected | Would not reduce effect | No | No | Very low |
| Dofetilide | Placebo or no treatment | All-cause mortality | Valembois 2019 | 3/3/0/0 | Serious risk | No serious inconsistency | No serious indirectness | Serious imprecision | Undetected | Would not reduce effect | No | No | Low |
| Dofetilide (PeAF) | Placebo or no treatment (PeAF) | All-cause mortality | Valembois 2019 | 3/3/0/0 | Serious risk | No serious inconsistency | No serious indirectness | Serious imprecision | Undetected | Would not reduce effect | No | No | Low |
| Flecanide | ProPeAFenone | All-cause mortality | Lafuente-Lafuente 2015 | 2/2/0/0 | Serious risk | Serious inconsistency | No serious indirectness | Serious imprecision | Strongly suspected | Would not reduce effect | No | No | Very low |
| Metoprolol | Placebo or no treatment | All-cause mortality | Valembois 2019 | 2/2/0/0 | Serious risk | Serious inconsistency | No serious indirectness | Serious imprecision | Undetected | Would not reduce effect | No | No | Very low |
| Metoprolol (PeAF) | Placebo or no treatment (PeAF) | All-cause mortality | Valembois 2019 | 2/2/0/0 | Serious risk | Serious inconsistency | No serious indirectness | Serious imprecision | Undetected | Would not reduce effect | No | No | Very low |
| Ranolazine | Control AADs (amiodarone, dronedarone, placebo) | Mortality | Guerra 2016 | 5/4/1/0 | Serious risk | Serious inconsistency | No serious indirectness | Serious imprecision | Undetected | Would not reduce effect | No | No | Very low |
| Sotalol | Class I drugs other than quinidine | All-cause mortality | Lafuente-Lafuente 2015 | 4/4/0/0 | Serious risk | Serious inconsistency | No serious indirectness | Serious imprecision | Strongly suspected | Would not reduce effect | No | No | Very low |
| Sotalol | ProPeAFenone | All-cause mortality at 12 months | Sullivan 2013 | 39/39/0/0 | Serious risk | Serious inconsistency | No serious indirectness | Serious imprecision | NA | Would not reduce effect | No | No | Very low |
| Quinidine | Placebo or no treatment | All-cause mortality | Valembois 2019 | 6/6/0/0 | Serious risk | No serious inconsistency | No serious indirectness | Serious imprecision | Undetected | Would not reduce effect | No | No | Low |
| Quinidine (PeAF) | Placebo or no treatment (PeAF) | All-cause mortality | Valembois 2019 | 5/5/0/0 | Serious risk | No serious inconsistency | No serious indirectness | Serious imprecision | Undetected | Would not reduce effect | No | No | Low |
| Quinidine | Other class I drugs | All-cause mortality | Lafuente-Lafuente 2015 | 2/2/0/0 | Serious risk | Serious inconsistency | No serious indirectness | Serious imprecision | Strongly suspected | Would not reduce effect | No | No | Very low |
| Quinidine (higher dose) | Placebo or no treatment | All-cause mortality | Valembois 2019 | 4/4/0/0 | Serious risk | No serious inconsistency | No serious indirectness | Serious imprecision | Undetected | Would not reduce effect | No | No | Low |
| Quinidine (lower dose) | Placebo or no treatment | All-cause mortality | Valembois 2019 | 2/2/0/0 | Serious risk | No serious inconsistency | No serious indirectness | Serious imprecision | Undetected | Would not reduce effect | No | No | Low |
| Quinidine | Sotalol | All-cause mortality | Lafuente-Lafuente 2015 | 6/6/0/0 | Serious risk | No serious inconsistency | No serious indirectness | Serious imprecision | Strongly suspected | Would not reduce effect | No | No | Very low |
| **Comorbidities** | | | | | | | | | | | | | |
| Cancer | No cancer | Cardiovascular mortality | Balomenakis 2023 | 6/2/4/0 | Serious risk | Serious inconsistency | No serious indirectness | Serious imprecision | Strongly suspected | Would not reduce effect | No | No | Very low |
| **Demographic characteristics** | | | | | | | | | | | | | |
| LVH | No LVH | All-cause mortality | Xiang 2021 | 3/2/1/0 | No serious risk | Serious inconsistency | No serious indirectness | Serious imprecision | NA | Would not reduce effect | No | No | Very low |
| Women | Men | Cardiovascular mortality | Marzona 2018 | 7/3/4/0 | Serious risk | No serious inconsistency | No serious indirectness | Serious imprecision | Undetected | Would not reduce effect | No | No | Very low |
| Women | Men | All-cause mortality | Marzona 2018 | 13/2/10/1 | Serious risk | Serious inconsistency | No serious indirectness | Serious imprecision | Undetected | Would not reduce effect | No | No | Very low |
| **Surgery** | | | | | | | | | | | | | |
| Ablation (RAF) | Medical therapy (RAF) | Mortality | Kheshti 2023 | 3/3/0/0 | Serious risk | Serious inconsistency | No serious indirectness | Serious imprecision | Undetected | Would not reduce effect | No | No | Very low |
| Ablation (PeAF) | Medical therapy (PeAF) | Mortality | Kheshti 2023 | 5/5/0/0 | Serious risk | Serious inconsistency | No serious indirectness | Serious imprecision | Undetected | Would not reduce effect | No | No | Very low |
| Biatrial maze | Left atrial maze | Early death | Zheng 2016 | 9/2/7/0 | Serious risk | No serious inconsistency | No serious indirectness | Serious imprecision | Undetected | Would not reduce effect | No | No | Very low |
| Biatrial maze | Left atrial maze | Overall death | Zheng 2016 | 9/2/7/0 | Serious risk | No serious inconsistency | No serious indirectness | Serious imprecision | Undetected | Would not reduce effect | No | No | Very low |
| Biatrial ablation | Isolated left atrial ablation | Mortality within 30 days | Li 2018 | 7/1/6/0 | Serious risk | No serious inconsistency | No serious indirectness | Serious imprecision | Undetected | Would not reduce effect | No | No | Very low |
| Biatrial ablation | Isolated left atrial ablation | Late mortality | Li 2018 | 4/1/3/0 | Serious risk | No serious inconsistency | No serious indirectness | Serious imprecision | Undetected | Would not reduce effect | No | No | Very low |
| Biventricular pacing mode ungergoing AVNA | Right ventricular pacing mode undergoing AVNA | All-cause mortality | Chatterjee 2012 | 4/4/0/0 | Serious risk | No serious inconsistency | No serious indirectness | Serious imprecision | NA | Would not reduce effect | No | No | Low |
| Biventricular pacing mode ungergoing AVNA | Right ventricular pacing mode undergoing AVNA | Cardiac mortality | Chatterjee 2012 | 3/3/0/0 | Serious risk | No serious inconsistency | No serious indirectness | Serious imprecision | NA | Would not reduce effect | No | No | Low |
| Catheter ablation | Medical therapy (rate control) | All-cause mortality | Asad 2019 | 2/2/0/0 | No serious risk | No serious inconsistency | No serious indirectness | Serious imprecision | NA | Would not reduce effect | No | No | Moderate |
| Catheter ablation (preserved LVEF) | Medical therapy (preserved LVEF) | Mortality | Ravi 2022 | 13/13/0/0 | Serious risk | No serious inconsistency | No serious indirectness | Serious imprecision | Undetected | Would not reduce effect | No | No | Low |
| Catheter ablation (no prior AADs use) | Medical therapy (no prior AADs use) | Mortality | Ravi 2022 | 6/6/0/0 | Serious risk | No serious inconsistency | No serious indirectness | Serious imprecision | Undetected | Would not reduce effect | No | No | Low |
| Catheter ablation (non-HF) | Medical therapy (non-HF) | All-cause mortality | Asad 2019 | 4/4/0/0 | No serious risk | No serious inconsistency | No serious indirectness | Serious imprecision | NA | Would not reduce effect | No | No | Moderate |
| LAAC during cardiac surgery | No LAAC | Postoperative mortality | Prasad 2022 | 14/2/12/0 | Serious risk | Serious inconsistency | No serious indirectness | Serious imprecision | Strongly suspected | Would not reduce effect | No | No | Very low |
| LAAO | No LAAO | All-cause mortality | Nso 2022 | 11/2/8/1 | Serious risk | Serious inconsistency | No serious indirectness | Serious imprecision | NA | Would not reduce effect | No | No | Very low |
| Left atrial concomitant surgical ablation in cardiac surgery | Cardiac surgery alone | 30-day mortality | Wang 2018 | 11/11/0/0 | Serious risk | No serious inconsistency | No serious indirectness | Serious imprecision | Undetected | Would not reduce effect | No | No | Low |
| Left atrial concomitant surgical ablation in cardiac surgery | Cardiac surgery alone | Late mortality | Wang 2018 | 7/7/0/0 | Serious risk | No serious inconsistency | No serious indirectness | Serious imprecision | NA | Would not reduce effect | No | No | Low |
| Pharmacotherapy | AVNA | Mortality | Chatterjee 2012 | 5/4/1/0 | Serious risk | Serious inconsistency | No serious indirectness | Serious imprecision | NA | Would not reduce effect | No | No | Very low |
| Surgical ablation in cardiac surgery | No surgical ablation | All-cause mortality | McClure 2018 | 23/23/0/0 | No serious risk | No serious inconsistency | No serious indirectness | Serious imprecision | Undetected | Would not reduce effect | No | No | Moderate |
| Surgical ablation in cardiac surgery | No surgical ablation | All-cause ICU mortality during index hospitalization | McClure 2018 | 7/7/0/0 | No serious risk | No serious inconsistency | No serious indirectness | Serious imprecision | Undetected | Would not reduce effect | No | No | Moderate |
| Surgical ablation in cardiac surgery | No surgical ablation | All-cause hospital mortality during index hospitalization | McClure 2018 | 15/15/0/0 | No serious risk | No serious inconsistency | No serious indirectness | Serious imprecision | Undetected | Would not reduce effect | No | No | Moderate |
| **Others (other medications, disease characteristics, treatment strategies)** | | | | | | | | | | | | | |
| RAAS blockers | No RAAS blockers | All-cause mortality | Chaugai 2016 | 6/6/0/0 | No serious risk | No serious inconsistency | No serious indirectness | Serious imprecision | Undetected | Would not reduce effect | No | No | Moderate |
| RAAS blockers | No RAAS blockers | Cardiovascular mortality | Chaugai 2016 | 5/5/0/0 | No serious risk | Serious inconsistency | No serious indirectness | Serious imprecision | Strongly suspected | Would not reduce effect | No | No | Very low |
| SGLT2 inhibitors | Placebo | All-cause mortality | Zheng 2024 | 5/5/0/0 | No serious risk | No serious inconsistency | No serious indirectness | Serious imprecision | NA | Would not reduce effect | No | No | Moderate |
| Asymptomatic AF | Symptomatic AF | All-cause mortality | Sgreccia 2021 | 6/1/5/0 | No serious risk | Serious inconsistency | No serious indirectness | Serious imprecision | Undetected | Would not reduce effect | No | No | Very low |
| Asymptomatic AF | Symptomatic AF | Cardiovascular mortality | Sgreccia 2021 | 4/1/3/0 | No serious risk | No serious inconsistency | No serious indirectness | Serious imprecision | Undetected | Would not reduce effect | No | No | Low |
| Exercise-based cardiac rehabilitation | Usual care | All-cause mortality | Smart 2018 | 8/8/0/0 | Serious risk | No serious inconsistency | No serious indirectness | Serious imprecision | Undetected | Would not reduce effect | No | No | Low |
| Rate control | Rhythm control | All-cause mortality | Depoorter 2020 | 4/0/4/0 | Serious risk | Serious inconsistency | No serious indirectness | Serious imprecision | NA | Would not reduce effect | No | No | Very low |
| Rate control | Rhythm control | Cardiovascular mortality | Depoorter 2020 | 2/0/2/0 | Serious risk | No serious inconsistency | No serious indirectness | Serious imprecision | NA | Would not reduce effect | No | No | Very low |

AF, Atrial fibrillation; PeAF, Persistent atrial fibrillation; RAF, Refractory atrial fibrillation; HF, Heart failure; AAD, Anti-arrhythmic Drugs; DOAC, Direct oral anticoagulant; NOAC, Non-vitamin K antagonist oral anticoagulant; OAC, Oral anticoagulant; CrCl, Creatinine clearance rate; BMI, Body mass index; TTR,Time in therapeutic range; TIA, Transient ischemic attack; VKA, Vitamin K antagonists; INR, International normalized ratio; RAAS, Renin-angiotensin-aldosterone system; SGLT2, Sodium-glucose transporter 2; AVNA, Atrioventricular nodal ablation; LVEF, Left ventricular ejection fractions; LAAC, Left atrial appendage closure; LAAO, Left atrial appendage occlusion; COPD, Chronic obstructive pulmonary disease; LA, Left atrial; LVH, Left ventricular hypertrophy; LD, low-dose; HD, high-dose; CI, confidence interval; T, total No. of studies; R, randomized controlled trials; C, cohort studies; P, population-based case-control and/or cross-sectional studies; AMSTAR, a measurement tool to assess systematic reviews; GRADE, Grading of Recommendations Assessment, Development and Evaluation; NA, not available.
