# Supplementary material for: Factors associated with mortality in patients with atrial fibrillation: an umbrella review of systematic reviews and meta-analyses
Source: Front Cardiovasc Med. 2026 Oct 13;13:1877770. doi: 10.3389/fcvm.2026.1877770 (PMC13710006; doi:10.3389/fcvm.2026.1877770)
Supplement: Supplementary file 6 [file Table6.docx]

Table S6. Summary of risk factors on mortality of AF patients.

| Outcome | Intervention | Comparator | Illustrative comparative risks (95% CI) | | Relative effect (95% CI) | Number of participants (studies) | Certainty of the evidence (GRADE) | Comments |
| --- | --- | --- | --- | --- | --- | --- | --- | --- |
|  |  |  | Assumed risk | Corresponding risk |  |  |  |  |
|  |  |  | With comparator | With intervention |  |  |  |  |
| All-cause mortality | Adjusted-dose warfarin | Other antithrombotic treatment or placebo | 973/2900 | 253/2900 | 0.26 [0.03 to 0.43] | 2900 | Low |  |
| Mortality | Apixaban | Rivaroxaban | 36512/878520 | 30305/878520 | 0.83 [0.71 to 0.96] | 878520 | Very low |  |
| All-cause mortality | Apixaban | Warfarin | NA | NA | 0.69 [0.49 to 0.98] | 404910 | Very low |  |
| All-cause mortality | Dabigatran 150mg | Warfarin | NA | NA | 0.65 [0.58 to 0.73] | NA | Very low |  |
| All-cause mortality | Dabigatran | Warfarin | NA | NA | 0.67 [0.57 to 0.80] | 606353 | Very low |  |
| Cardiovascular mortality | DOAC (Diabetes) | VKA (Diabetes) | 851/13054 | 715/13054 | 0.84 [0.72 to 0.97] | 13054 | Low |  |
| All-cause mortality | DOAC (No Diabetes) | VKA (No Diabetes) | 1737/22940 | 1546/22940 | 0.89 [0.81 to 0.99] | 22940 | Low |  |
| Mortality | DOAC | Warfarin | 3098/23241 | 1518/23241 | 0.49 [0.43 to 0.56] | 23241 | Low |  |
| All-cause mortality | DOAC (BMI 25-30) | Warfarin (BMI 25-30) | 1990/57866 | 1672/57866 | 0.84 [0.76 to 0.92] | 57866 | High |  |
| Mortality | DOAC with polypharmacy  (≥ 5 concomitant drugs) | Warfarin with polypharmacy  (≥ 5 concomitant drugs) | NA | NA | 0.91 [0.83 to 0.99] | 20371 | Moderate |  |
| Mortality | DOAC without amiodarone | Warfarin without amiodarone | NA | NA | 0.92 [0.86 to 0.98] | 52137 | Moderate |  |
| Mortality | DOAC without P-glycoprotein inhibitor | Warfarin without P-glycoprotein inhibitor | NA | NA | 0.91 [0.84 to 0.98] | 46117 | Moderate |  |
| Mortality | Edoxaban 30mg | Aspirin | 2268/14777 | 1860/14777 | 0.82 [0.69 to 0.98] | 14777 | Low |  |
| Mortality | Edoxaban 30mg | Placebo | 1308/8459 | 837/8459 | 0.64 [0.46 to 0.87] | 8459 | Low |  |
| Mortality | Edoxaban 30mg | Warfarin | 4065/47119 | 3577/47119 | 0.88 [0.80 to 0.97] | 47119 | Low |  |
| Vascular mortality | Edoxaban 30mg with concomitant aspirin therapy | Warfarin with concomitant aspirin therapy | 524/10261 | 393/10261 | 0.75 [0.59 to 0.96] | 10261 | Low |  |
| Mortality | Edoxaban 60mg | Placebo | 1303/8460 | 873/8460 | 0.67 [0.49 to 0.92] | 8460 | Low |  |
| Cardiovascular mortality | Edoxaban 60mg | Warfarin | NA | NA | 0.87 [0.78 to 0.97] | 14799 | Low |  |
| All-cause mortality | Edoxaban | Warfarin | NA | NA | 0.52 [0.31 to 0.85] | 32995 | Very low |  |
| Mortality | HD-Rivaroxaban | HD-Dabigatran | NA | NA | 1.27 [1.09 to 1.46] | 147261 | Very low |  |
| Mortality | LD-Rivaroxaban | LD-Dabigatran | NA | NA | 1.17 [1.05 to 1.29] | 147261 | Very low |  |
| Mortality | NOAC (TTR < 65%) | Warfarin (TTR < 65%) | 1695/18114 | 1441/18114 | 0.85 [0.76 to 0.93] | 18114 | High |  |
| Vascular mortality | NOAC with concomitant aspirin therapy | Warfarin with concomitant aspirin therapy | 1026/21335 | 872/21335 | 0.85 [0.76 to 0.93] | 21335 | High |  |
| Mortality | NOAC (No previous stroke or TIA) | Warfarin (No previous stroke or TIA) | 3004/36050 | 2704/36050 | 0.90 [0.84 to 0.97] | 36050 | Moderate |  |
| All-cause mortality | NOAC (non-Latin American) | Warfarin (non-Latin American) | NA | NA | 0.90 [0.84 to 0.97] | 62627 | Moderate |  |
| All-cause mortality | Non−Ximelagatran NOAC | Control drugs | 4648/61088 | 4137/61088 | 0.89 [0.84 to 0.95] | 61088 | High |  |
| All-cause mortality | Oral Factor Xa inhibitors | VKA | 4660/56438 | 4101/56438 | 0.88 [0.83 to 0.94] | 56438 | High |  |
| Early fatality (30 days) | Therapeutic INR (ischemic stroke only) | Other antithrombotic categories (ischemic stroke only) | NA | NA | 0.35 [0.23 to 0.52] | 3429 | Low |  |
| Late fatality (1 year) | Therapeutic INR (ischemic stroke only) | Other antithrombotic categories (ischemic stroke only) | NA | NA | 0.40 [0.25 to 0.64] | 1411 | Low |  |
| All-cause mortality | Warfarin | Placebo | 342/2900 | 253/2900 | 0.74 [0.57 to 0.97] | 2900 | Low |  |
| All-cause mortality | Class IA drugs | No treatment, placebo, drugs for rate control | 11/1797 | 27/1797 | 2.39 [1.03 to 5.59] | 1797 | Low |  |
| All-cause mortality | Digoxin | Beta-blockers | 13246/85729 | 24107/85729 | 1.82 [1.65 to 2.01] | 85729 | Low |  |
| All-cause mortality | Digoxin | Calcium channel blockers | 15255/59312 | 20899/59312 | 1.37 [1.06 to 1.78] | 59312 | Very low |  |
| All-cause mortality | Digoxin | No digoxin | NA | NA | 1.17 [1.13 to 1.22] | 621478 | Very low |  |
| Cardiovascular mortality | Digoxin | No digoxin | NA | NA | 1.27 [1.08 to 1.50] | 63975 | Very low |  |
| Sudden cardiac death | Digoxin | No digoxin | NA | NA | 1.40 [1.23 to 1.60] | 48948 | Very low |  |
| All-cause mortality | Digoxin  (AF without HF) | No digoxin  (AF without HF) | NA | NA | 1.28 [1.11 to 1.47] | NA | Very low |  |
| Arrhythmic mortality | Dronedarone with digoxin | Placebo with digoxin | 5/3236 | 17/3236 | 3.26 [1.06 to 10.00] | 3236 | Low |  |
| All-cause mortality | Dronedarone  (Digoxin at baseline) | Placebo  (Digoxin at baseline) | 4/1070 | 20/1070 | 5.47 [1.60 to 18.66] | 1070 | Low |  |
| Arrhythmic mortality | Dronedarone  (Digoxin at baseline) | Placebo  (Digoxin at baseline) | 0/1070 | 11/1070 | 22.7 [1.33 to 386.17] | 1070 | Low |  |
| All-cause mortality | Sotalol | Placebo | 18/3058 | 40/3058 | 2.23 [1.10 to 4.50] | 3058 | Low |  |
| All-cause mortality | Sotalol | Placebo or no treatment | 18/1882 | 40/1882 | 2.23 [1.03 to 4.81] | 1882 | Moderate |  |
| All-cause mortality | Sotalol (PeAF) | Placebo or no treatment (PeAF) | 15/1311 | 37/1311 | 2.51 [1.06 to 5.98] | 1311 | Moderate |  |
| All-cause mortality | Diabetes | No diabetes | NA | NA | 1.37 [1.23 to 1.53] | 518965 | Very low |  |
| Cardiovascular mortality | Diabetes | No diabetes | NA | NA | 1.46 [1.34 to 1.58] | 50092 | Very low |  |
| All-cause mortality | Anemia | No Anemia | NA | NA | 1.78 [1.44 to 2.20] | 75990 | Very low |  |
| All-cause mortality | Cancer | No cancer | 312515/3000762 | 678158/3000762 | 2.17 [1.83 to 2.56] | 3000762 | Very low |  |
| Non-Cardiovascular mortality | Cancer | No cancer | NA | NA | 6.30 [2.32 to 17.33] | 89082 | Very low |  |
| All-cause mortality | COPD | No COPD | 42312/220953 | 93933/220953 | 2.22 [1.93 to 2.55] | 93933/220953 | Very low |  |
| Cardiovascular mortality | COPD | No COPD | 1783/73956 | 3280/73956 | 1.84 [1.39 to 2.43] | 3280/73956 | Very low |  |
| All-cause mortality | Frailty | No frailty | NA | NA | 2.77 [1.68 to 4.57] | 97413 | Low |  |
| All-cause mortality | LVH | No LVH | NA | NA | 1.60 [1.42 to 1.79] | 13230 | Very low |  |
| All-cause mortality | Peripheral artery disease | No peripheral artery disease | NA | NA | 1.42 [1.25 to 1.62] | 30286 | Low |  |
| All-cause mortality | High BMI | Low BMI | 10438/109478 | 8977/109478 | 0.86 [0.80 to 0.92] | 8977/109478 | Very low |  |
| Cardiovascular mortality | High BMI | Low BMI | 2656/63875 | 2178/63875 | 0.82 [0.71 to 0.95] | 2178/63875 | Very low |  |
| All-cause mortality | Latin American  (Warfarin) | Non-Latin American (Warfarin) | NA | NA | 1.45 [1.20 to 1.76] | 13058 | High |  |
| All-cause mortality | Latin American  (NOAC) | Non-Latin American  (NOAC) | NA | NA | 1.46 [1.27 to 1.68] | 26160 | High |  |
| All-cause mortality | Latin American (NOAC+Warfarin) | Non-Latin American (NOAC+Warfarin) | NA | NA | 1.46 [1.30 to 1.63] | 39218 | High |  |
| All-cause mortality | Overweight (BMI 25-30) | Normal BMI | NA | NA | 0.78 [0.62 to 0.96] | 44441 | Very low |  |
| All-cause mortality | Obesity (BMI ≥ 30) | Normal BMI | 5407/55351 | 3298/55351 | 0.61 [0.52 to 0.71] | 3298/55351 | Very low |  |
| All-cause mortality | Smoking | No smoking | NA | NA | 1.82 [1.33 to 2.49] | 17472 | Very low |  |
| Cardiovascular mortality | Smoking | No smoking | NA | NA | 1.54 [1.31 to 1.81] | 4420 | Very low |  |
| All-cause mortality | Underweight (BMI < 18.5) | Normal BMI | 493/34096 | 1759/34096 | 3.57 [2.50 to 5.11] | 34096 | Low |  |
| All-cause mortality | Elderly group  (＞75 years old) | Younger group  (< 75 years old) | 166/101062 | 433/101062 | 2.61 [1.38 to 4.93] | 101062 | Very low |  |
| Cardiovascular mortality | Ablation | Drug therapy | NA | NA | 0.35 [0.25 to 0.50] | 19260 | Low |  |
| All-cause mortality | Ablation | Drug therapy | NA | NA | 0.64 [0.45 to 0.93] | 5804 | Very low |  |
| All-cause mortality | Catheter Ablation | Non-catheter ablation | NA | NA | 0.60 [0.51 to 0.71] | 270123 | Very low |  |
| All-cause mortality | Catheter Ablation (HF) | Medical therapy (HF) | 188/668 | 98/668 | 0.52 [0.35 to 0.76] | 668 | High |  |
| All-cause mortality | Catheter Ablation (HF+Non-HF) | Medical therapy (HF+Non-HF) | 342/3576 | 236/3576 | 0.69 [0.54 to 0.88] | 3576 | Moderate |  |
| All-cause mortality | Catheter Ablation | AAD | 70/619 | 32/619 | 0.46 [0.23 to 0.93] | 619 | High |  |
| Mortality | Catheter ablation  (Reduced LVEF) | Medical therapy (Reduced LVEF) | 202/877 | 113/877 | 0.56 [0.39 to 0.79] | 1877 | Low |  |
| All-cause mortality | LAAC | OAC | 3869/25548 | 2592/25548 | 0.67 [0.57 to 0.78] | 25548 | Very low |  |
| Cardiovascular mortality | LAAC | OAC | 750/5358 | 465/5358 | 0.62 [0.51 to 0.74] | 465/5358 | Low |  |
| All-cause mortality > 2 years | LAAC during cardiac surgery | No LAAC | 12573/307232 | 10184/307232 | 0.81 [0.68 to 0.97] | 10184/307232 | Very low |  |
| Mortality | LAAO | DOAC | NA | NA | 0.75 [0.55 to 0.99] | 94756 | Very low |  |
| Mortality | LAAO | Warfarin | NA | NA | 0.68 [0.50 to 0.90] | 94756 | Low |  |
| Cardiovascular mortality | SGLT2 inhibitors | Placebo | 1020/9872 | 887/9872 | 0.87 [0.76 to 0.98] | 9872 | Moderate |  |
| All-cause mortality | Statin (low-to-moderate intensity statin) | No statin | NA | NA | 0.53 [0.30 to 0.94] | NA | Very low |  |
| All-cause mortality | Statin (high intensity statin) | No statin | NA | NA | 0.47 [0.35 to 0.63] | NA | Very low |  |
| All-cause mortality | Statin  (high intensity statin) | Statin  (low-to-moderate intensity statin) | NA | NA | 0.70 [0.53 to 0.92] | NA | Very low |  |
| All-cause mortality | Statin | No statin | 22666/86356 | 13373/86356 | 0.59 [0.54 to 0.65] | 86356 | Very low |  |
| Cardiovascular mortality | Statin | No statin | 627/15102 | 470/15102 | 0.75 [0.58 to 0.96] | 15102 | Very low |  |
| All-cause mortality | Paroxysmal AF | Persistent AF | 5461/48973 | 3932/48973 | 0.72 [0.66 to 0.79] | 48973 | High |  |
| All-cause mortality | Non-paroxysmal AF | Paroxysmal AF | 3104/7834 | 5897/7834 | 1.90 [1.43 to 2.52] | 7834 | Very low |  |
| All-cause mortality | Early rhythm control | Rate control | 163662/370677 | 124383/370677 | 0.76 [0.69 to 0.83] | 370677 | Very low |  |
| Cardiovascular mortality | Early rhythm control | Rate control | 37466/320176 | 25477/320176 | 0.68 [0.63 to 0.74] | 320176 | Very low |  |

PeAF, Persistent atrial fibrillation; AF, Atrial fibrillation; RAF, Refractory atrial fibrillation; HF, Heart failure; AAD, Anti-arrhythmic Drugs; DOAC, Direct oral anticoagulant; NOAC, Non-vitamin K antagonist oral anticoagulant; OAC, Oral anticoagulant; CrCl, Creatinine clearance rate; BMI, Body mass index; TTR,Time in therapeutic range; TIA, Transient ischemic attack; VKA, Vitamin K antagonists; INR, International normalized ratio; RAAS, Renin-angiotensin-aldosterone system; SGLT2, Sodium-glucose transporter 2; AVNA, Atrioventricular nodal ablation; LVEF, Left ventricular ejection fractions; LAAC, Left atrial appendage closure; LAAO, Left atrial appendage occlusion; COPD, Chronic obstructive pulmonary disease; LA, Left atrial; LVH, Left ventricular hypertrophy; LD, low-dose; HD, high-dose; AMSTAR, a measurement tool to assess systematic reviews; GRADE, Grading of Recommendations Assessment, Development and Evaluation; NA, not available; NS, not significant; HR, hazard ratio; OR, odds ratio; RR, risk ratio; MA, meta analysis; CI, confidence interval.
